# Formation and Reactivity of an Elusive Monomeric Mn(IV)-Oxo Species Inside a Cavitand Pore

**DOI:** 10.1021/jacs.5c02637

**Published:** 2025-08-07

**Authors:** Galon Green, Kamal Uddin Ansari, Thejasree Munikrishna, Sagi Ezov, Donia Shamali, Laxmi-Narayan Nanda, Vitaly Gutkin, Orit Cohen, Daphna Shimon, Yuri Tulchinsky

**Affiliations:** Institute of Chemistry, 208979 the Hebrew University of Jerusalem, Jerusalem 9190401, Israel

## Abstract

Metal-functionalized cavitands are promising platforms for mimicking the chemical environments of hydrophobic pockets in natural metalloenzymes. However, successfully combining the unique supramolecular capabilities of cavitand scaffolds with the high reactivities of transition metal complexes still remains a major challenge. In this study, we present an original cavitand architecture featuring a coordinatively unsaturated Mn­(II) center embedded deep within its pore. This metallocavitand was employed to generate a Mn­(IV)-oxo species inside a molecular cavity. This elusive intermediate was fully characterized spectroscopically (UV–vis, EPR, X-ray photoelectric spectroscopy (XPS), and HRMS) and, for the first time for a pseudo-octahedral Mn­(IV)-oxo species, also by XRD. The experimental data was corroborated by detailed *ab initio*/time-dependent density functional theory (TDDFT) and natural bond orbital (NBO) calculations, confirming the Mn­(IV)-oxo (rather than Mn­(III)-oxyl) electronic character of this species. Reactivity and mechanistic studies, including monitoring the decay of this complex in various chlorinated solvents and its reactions with representative substrates, revealed that, despite the steric protection provided by the cavitand scaffold, its Mn­(IV)-oxo core remains highly reactive in both H atom abstraction (HAA) and O atom transfer (OAT) reactions. Moreover, this reactivity is subject to a high degree of steric control imposed by the cavitand framework capable of discriminating between potential substrate molecules based on their size and shape. This was further demonstrated by the regioselective oxidation of a bisphosphine substrate, emulating the regioselectivity of natural metalloenzymes.

## Graphical abstract

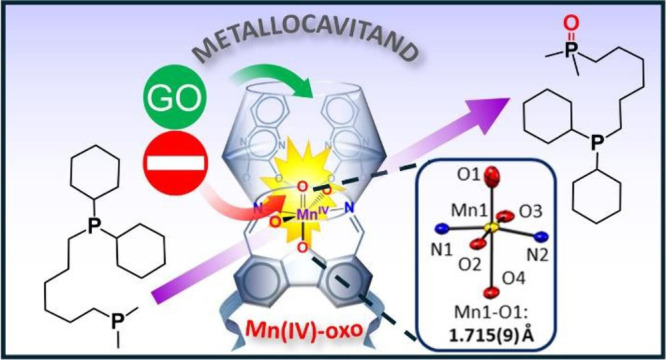

## Introduction

Terminal high-valent metal oxo species have long been identified as key intermediates involved in the operation of the ubiquitous Cytochrome P450 ^1^ and Photosystem II^2,3^ metalloenzyme families. Over the past decades, a plethora of synthetic analogs of these systems have been developed - some of them demonstrating impressive efficiency in C–H bond activation and oxygen atom transfer (OAT) reactions.^4^ Perhaps the most studied metal-oxo compounds are those of iron, exhibiting catalytic abilities unrivaled by other metal-oxos. In contrast, high-valent manganese-oxo systems are less explored, primarily due to their poor stability and complicated reactivity, often significantly deviating from the established “rebound” mechanism which dominates the Fe-oxo chemistry.^5–7^ Of the several oxidation states reported for terminal Mn-oxo complexes (III, IV, and V), Mn­(IV)-oxo is by far the most elusive, making its study beyond spectroscopic techniques and computational methods highly challenging.^4^ To better understand the structure–function relationship of this important intermediate, a significant body of work on such systems was carried out, first by Suslick^8^ with the “picket-fence” porphyrins^9,10^ (complex **I**, Figure 1a) and later by Fukuzumi and Nam,^11^ along with various other poly pyridyl ligands first reported by the same authors^12–16^ and later modified and extensively studied by Jackson^17,18^ and others^19,20^ (complexes **II** and **III**, Figure 1a).

**Figure 1:**
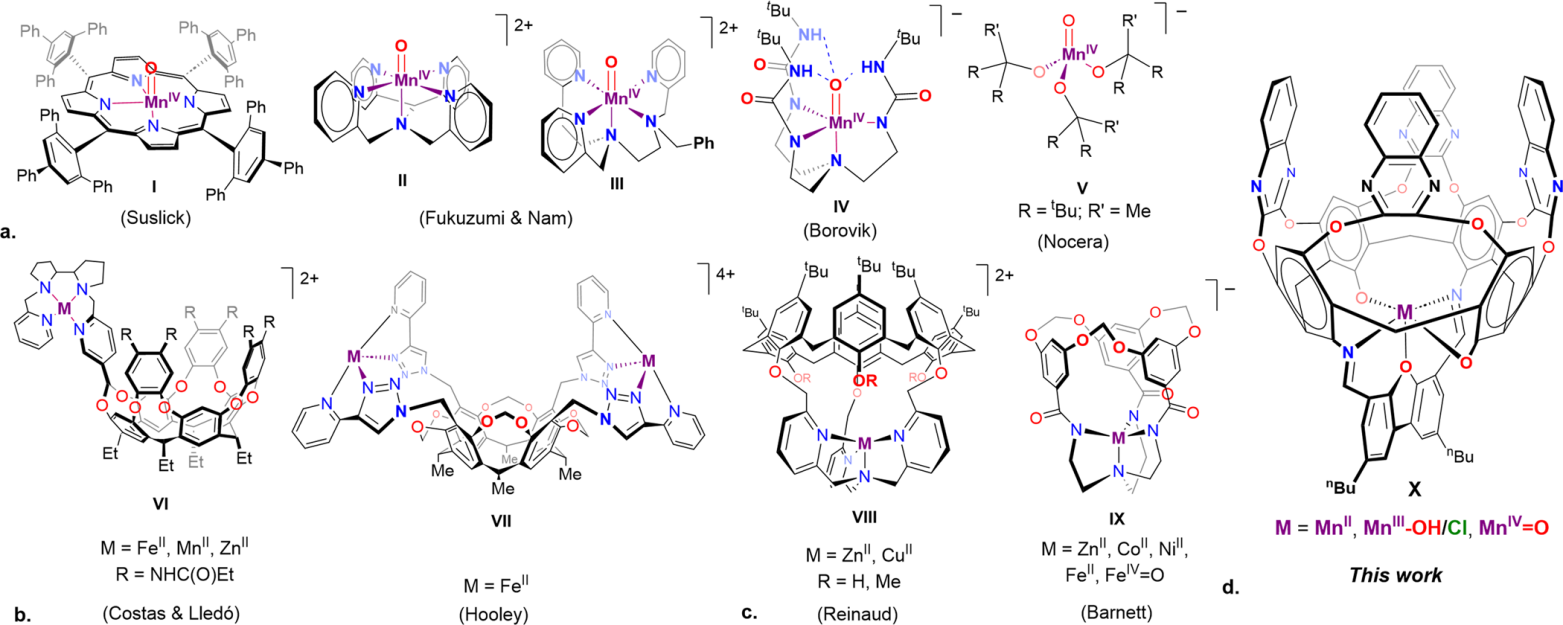
(a) Reported Mn­(IV)-oxo systems: picket-fence porphyrins (**I**), polypyridyl NPy4 and Bn-TPEN complexes (**II** and **III**), trigonal TREN-based complex stabilized by intramolecular H-bonding (**IV**), and the pseudotetrahedral tris-alkoxide complex (**V**); (b) resorcin[4]­arene-based metallocavitands representing the “dangling arm” approach (**VI** and **VII**); (c) tripod-based cavitands with an embedded metal center (**VIII** and **IX**); (d) metallocavitand system reported in this work (**X**).

These studies highlighted the dramatic reactivity enhancement that could be achieved by varying the primary and the secondary coordination spheres around the metal-oxo center. Indeed, some Mn-oxo systems have demonstrated exceptional chemical (and in some cases even catalytic) reactivities and selectivities, rivaling natural metalloenzymes in both OAT and hydrogen atom abstraction (HAA) reactions.^12,13,16,20–23^ Due to their highly reactive nature, only a handful of XRD structures of monomeric Mn-oxo complexes have been obtained. Most of them feature Mn­(V)-oxo species, which are sufficiently stabilized by means of strongly electron donating ligands, such as macrocyclic porphyrins^24^ and corrolazines,^25^ or chelating dialkoxy-diamido^26–28^ and tetra-amido^29–31^ ligands. In addition, several crystal structures of Mn­(III)-oxo species supported by tripodal ligands were reported by Borovik^32–34^ and others.^35^ In addition to the electronic stabilization, gained due to shifting from a tetragonal to a trigonal ligand field (an effect well-known for *d* ^4^ and *d* ^3^ metal-oxos),^36^ these *tris*-urea containing ligands also stabilized the Mn­(III)-oxo moiety through three intramolecular hydrogen bonds. However, the corresponding Mn­(IV)-oxo species (complex **IV**, Figure 1a) could only be obtained in solution at −80 °C and was too reactive for isolation in the solid state.^3^ In fact, the only known example of a crystallographically characterized Mn­(IV)-oxo complex has been reported by Nocera in 2018 (complex **V**, Figure 1a).^37^ In this case, the necessary stabilization was achieved by the combination of a pseudotetrahedral C_3v_ symmetry with the bulky and highly electron-donating *ditox* ligands.

The strategies mentioned above, employed to stabilize Mn-oxo species for enabling higher resolution of structural characterization, have mostly focused on modifying their primary coordination sphere. Thus, these methods inevitably suppress their intrinsic reactivity. An alternative approach would involve introducing steric protection by secondary-sphere interactions; yet, no crystal structures of Mn­(IV)-oxo systems utilizing this concept have been reported so far.

Since their first preparation by Cram,^38^ resorcinarene-based cavitands have been extensively studied as possible platforms for constructing molecular systems with distinct enzyme-mimicking properties, i.e., substrate recognition,^39,40^ stabilization of reactive intermediates,^41^ and reaction rate acceleration.^42–44^ The enzyme-like reactivity in these cavitands was often achieved by means of introducing inward-oriented reactive functional groups (such as carboxylic acids,^39^ aldehydes,^44^ or esters^45^) within their cavities. Building on the same concept, the idea of mimicking natural metalloenzymes by functionalizing the cavitands with catalytically active transition metals was first raised by Rebek^46,47^ and further explored by him and others in a series of subsequent works.^48–55^ In almost all such systems, the metal center was introduced via the *“dangling arm”* approach, i.e., by appending the metal coordination site to the aromatic walls at the upper rim of the cavitand (complexes **VI** and **VII**, Figure 1b).^50,56^ While this architecture is easier to achieve synthetically, it inevitably exposes the metal center not only to the interior of the cavitand but also to the bulk solution. As a result, such cavitand systems were often found to have little to no effect on the reactivity of the appended catalyst, compared to a native one.^56^

Conversely, cavitand-based systems where the metal center is buried deep within their molecular cavity are expected to better mimic the active sites of metalloenzymes, often located inside hydrophobic pockets. However, only a limited number of such cavitand architectures have been reported to date. These frameworks have typically been constructed either by functionalizing cyclodextrins,^57^ which exhibit shallow and ill-defined cavities, or by elaborating upon chelating podant-type scaffolds.^58^ A noteworthy example of the latter approach was demonstrated by Reinaud, who assembled a biomimetic cavitand system by capping a tetradentate tripodal ligand with a calix[6]-arene macrocycle (complex **VIII**, Figure 1c).^59,60^ Most recently, a structurally related cavitand design was utilized by Barnett for generating a unique thermally robust high-spin Fe­(IV)-oxo species (complex **IX**, Figure 1c).^61^ While efficiently providing steric protection to the embedded metal centers, the congested architecture of the podant-based cavitands imposes severe limitations on guest accessibility. In the case of system **IX**, this suppresses any HAA and OAT reactivity, even toward small substrate molecules.

In contrast, the larger and more flexible resorcin[4]­arene-based systems offer more promise for constructing efficient metalloenzyme mimics. Yet, the skeletons of traditional resorcinarene-based cavitands are poorly suited for this purpose, as they lack modifiable functional groups necessary for incorporating metal-binding motifs at their lower rim. This limitation prompted us to introduce a new macrocyclic precursor, allowing for sequential modification of both its upper and lower rims, thereby granting access to previously unattainable cavitand architectures.

Herein, we report the design and synthesis of a conceptually novel metallocavitand system (complex **X**, Figure 1d), featuring a rigid pentacoordinate metal-binding cage located at its lower-rim. Such an architecture effectively shields the coordinated metal center from the bulk of the solution and ensures that the former is accessible only from within the molecular pore. This design enabled the isolation and full characterization (including by XRD) of a highly reactive Mn­(IV)-oxo species in a pseudo-octahedral ligand field. Moreover, in this study, we explore the reactivity of this unique system and demonstrate that the cavitand framework imposes size and shape selectivity on the HAA and OAT reactions it undergoes.

## Results and Discussion

### Synthesis of the Cavitand Ligand Framework

To achieve the envisioned metallocavitand architecture, a suitable macrocyclic precursor with appropriately positioned modifiable groups was required. Such a compound was prepared by the acid-catalyzed condensation of 3,5-dimethoxyphenol and paraformaldehyde (Scheme 1), similarly to the reported calix[4]- and calix[5]­tetrolarenes.^62^ The resulting macrocycle **1**, which we suggest naming *triol­[4]­arene*, can be considered a hybrid between a calix[4]­arene and a resorcin[4]­arene, as it features oxygen-based functionalities on both its lower and upper rims. While the lower rim contains free hydroxyl groups, those located at the upper rim are protected as methyl ethers - enabling an orthogonal functionalization of each rim. In the crystal phase, this macrocycle adopts a *pinched-cone* conformation (Figure 2a), which is stabilized by four intramolecular H-bonds between the lower-rim hydroxyls, as is typical for calix[4]­arenes.^63^ Next, following a method originally developed for the 1,3-derivatization of *p*-^t^Bu-calix­[4]­arene,^64^ the two distal hydroxyl groups at the lower rim of **1** were converted into the primary amino functionalities via intermediates **2** and **3** (the latter being a mixture of two atropisomers A and B; see SI) (Scheme 1 and Figure 2b,c). The subsequent annulation of the resulting diamino derivative **4** to form a rigid pentacoordinate cage required a bridging unit of an appropriate size and bite angle. Previous reports of analogous trans-annular bridges in calix[4]- and calix[6]­arenes were limited to long-chain polyethers, which form large flexible loops unsuitable for transition metal binding.^65,66^ On the contrary, employing shorter and more rigid linkers (e.g., 1,1′-di­(acyl)­metallocenes) resulted in only dimeric and trimeric products.^67^

**Scheme 1:**
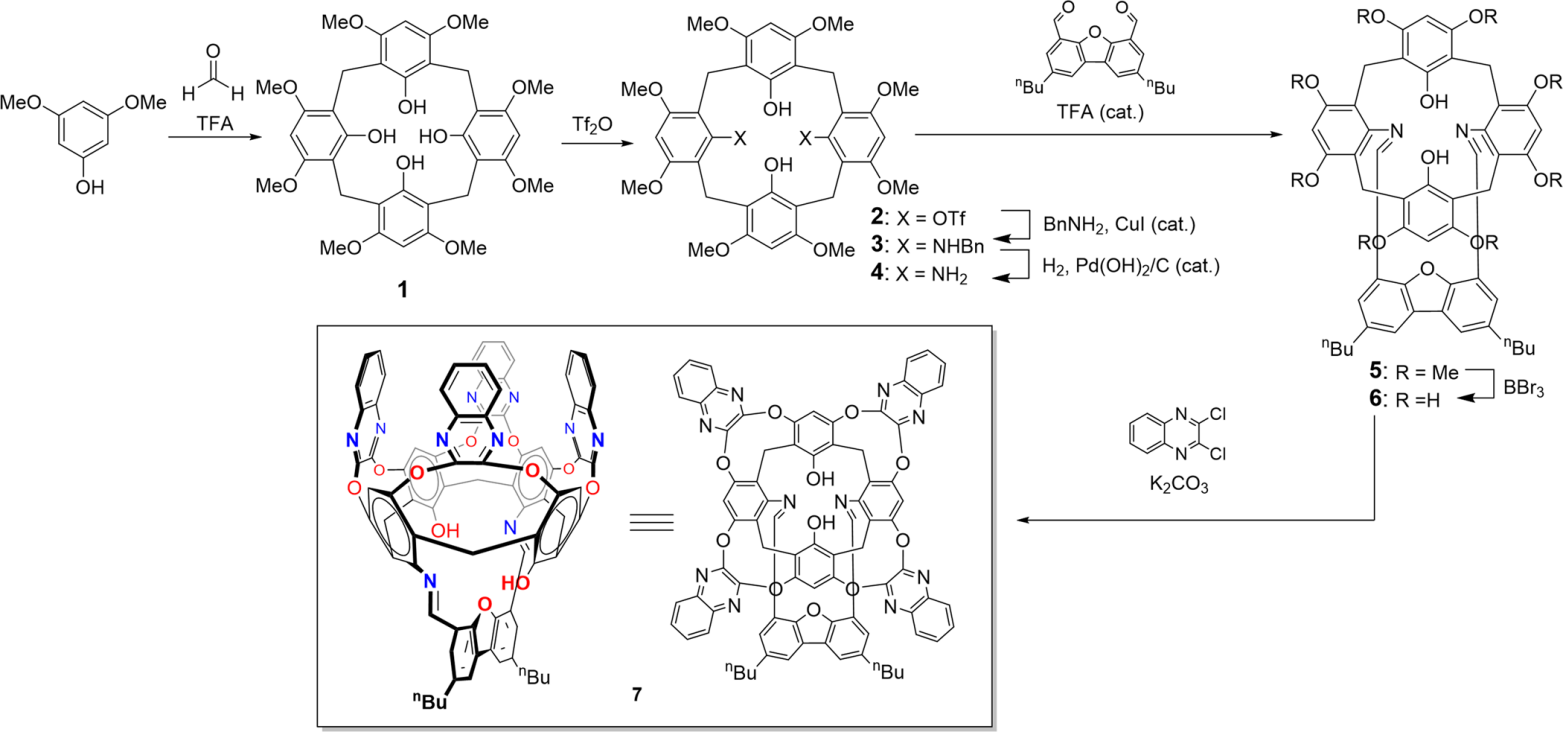
Synthesis of the Cavitand Ligand Framework

**Figure 2:**
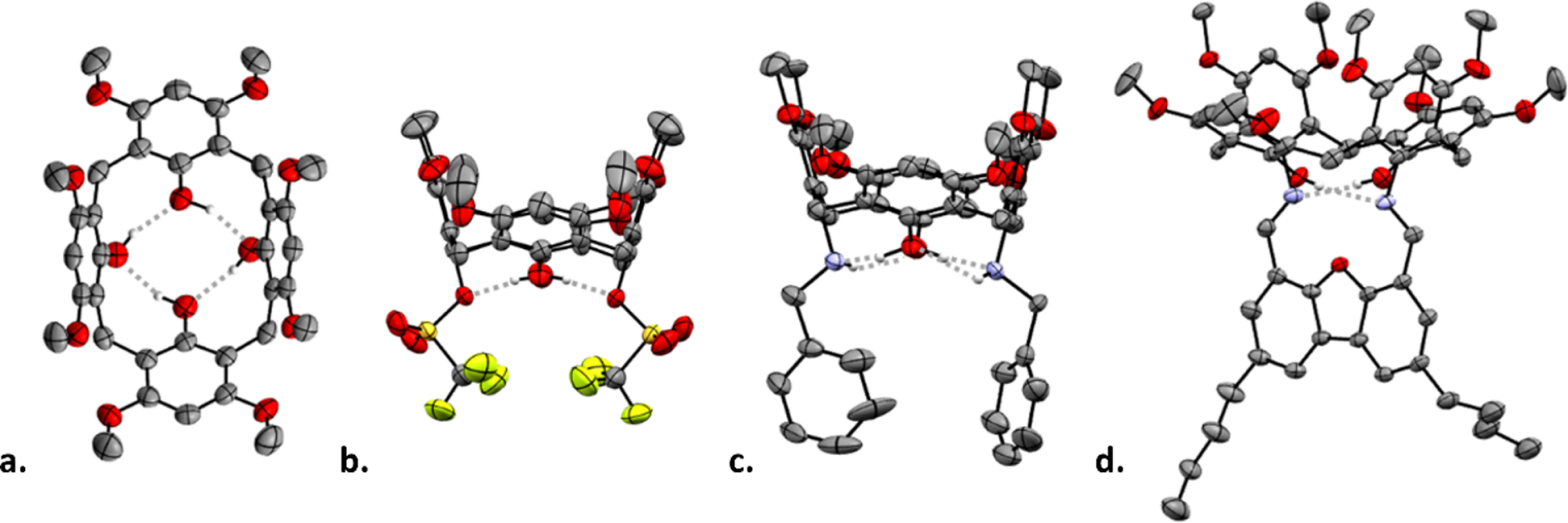
XRD structures of macrocyclic precursor **1** (a) and intermediates **2** (b), **3** (atropisomer A) (c), and **5** (d). All hydrogen atoms except those engaged in intramolecular H-bonds (represented by gray dashed lines) were omitted for clarity.

To identify the best candidate for the bridging unit, we performed a set of preliminary density functional theory (DFT) calculations, considering possible condensation products of **4** with three representative *bis*-aldehyde-functionalized heterocycles. The candidates chosen vary widely in their bite angles (Scheme S1 and the explanations therein). While these hypothetical reactions were predicted to be endothermic, our calculations revealed a significant preference of this system for the dibenzofuran derivative 5*_DBF_, which provides a bite angle of ∼90° (Δ*E* = +16.0 kcal/mol) over the pyridine and xanthene counterparts, 5*_Pyr_ and 5*_Xant_, with bite angles of ∼0° and ∼120° (Δ*E* = +31.2 and 31.6 kcal/mol), respectively. Based on these findings, we reacted compound **4** with a previously reported 2,8-dibutyl- functionalized dibenzofuran-4,6-dicarbaldehyde^68^ - resulting in the formation of a monomeric diimine **5** as the sole product (Scheme 1). Presence of the *n*-butyl tails in the former bis aldehyde was crucial for ensuring an adequate solubility of the diimine product. Having thus completed the construction of a metal-binding cage at the lower rim of the designed cavitand, we proceeded to build its aromatic walls at the upper rim. This was achieved by a complete demethylation of **5** to obtain the deca-hydroxy derivative **6**, followed by an etherification with four equivalents of 2,3-dichloroquinoxaline, each reacting with a pair of adjacent hydroxyl groups in accordance with established protocols^38,69,70^ (Scheme 1).

The final cavitand product **7** was obtained in moderate yield (45% from **6**) as an amorphous white powder. In the solid state, this compound adopts a *vase* conformation (Figure 3), similar to that previously observed by Cram and Rebek,^48,70^ with disordered DCM molecules (not shown) occupying its molecular cavity. This semirigid structure, reinforced by two intramolecular H-bonds (between the imine nitrogens and the phenolic protons), effectively preorganizes the coordination cage for metal complexation.

**Figure 3:**
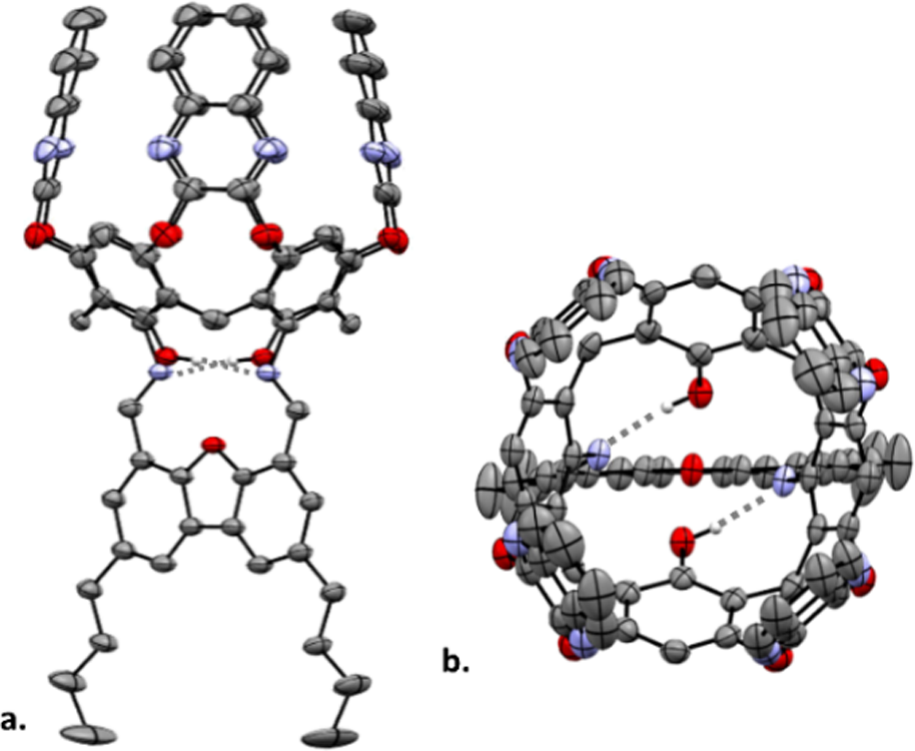
XRD structure of cavitand ligand **7**: (a) side view and (b) top view. All hydrogens, except those engaged in intramolecular H-bonds (represented by dashed gray lines), were omitted for clarity.

### Synthesis and Characterization of a Mn­(II)-Metallocavitand

Metalation of the cavitand with Mn­(II) was carried out by reacting the former with two equiv of LiHMDS in anhydrous THF, followed by the addition of a small excess of Mn­(OTf)_2_(MeCN)_2_ (Scheme 2). Soon after the addition, a very fine light purple powder began to precipitate from the reaction solution. This product was identified as the Mn­(II)-metallocavitand **8**, based on the molecular ion mass of [M + H]^+^ = 1408.3250 amu, consistent with its empirical formula, detected by ESI-HRMS. This compound was nearly insoluble in THF or MeCN but could be easily dissolved in chlorinated solvents, such as DCM, which allowed for the measurement of its effective magnetic moment using the Evans method.^71^ The obtained value of μ_eff_ = 5.58 μ_B_ was somewhat lower than the theoretical spin only moment for an *S* = 5/2 system (5.91 μ_B_) but still within the typical range observed in high-spin Mn­(II) complexes (5.5–6.2 μ_B_).^72,73^

**Scheme 2:**
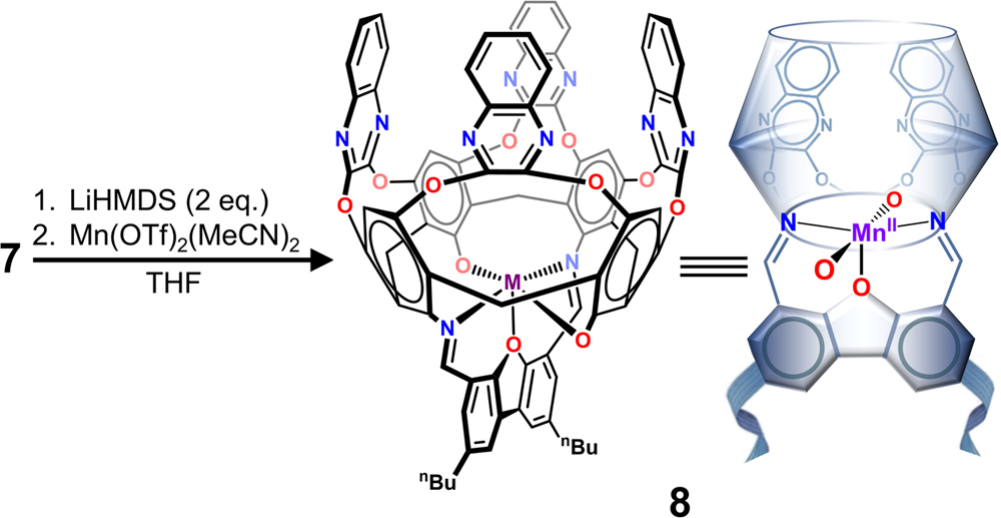
Metallation of Ligand **7** with Mn­(II)

This spin state assignment of **8** was further confirmed by an X-band CW-EPR measurement performed on a ground microcrystalline sample at 5 K (Figure S27), showing a broad highly anisotropic signal in the 500–10000 Oe magnetic field range. Simulation of the experimental spectrum resulted in axial and rhombic ZFS parameters and **g**-tensor values in reasonable agreement with the values reported for penta-coordinate Mn­(II) complexes.^74,75^ These parameters were also corroborated by the CASSCF/NEVPT2 calculations performed on a model complex 8*, a simplified version of **8**, lacking the quinoxaline walls and with the butyl tails truncated (Table S6).

XRD quality crystals of metallocavitand **8** were obtained as a water adduct (**8**·H_2_O) upon crystallization of this complex from DCM under an ambient atmosphere. The refined structure shows a single Mn­(II) ion rigidly fixed at the bottom of the molecular cavity with a water molecule completing the octahedral configuration around the metal center (Figure 4a). The Mn–N and Mn–O bond lengths of 2.185(3) Å and 2.071(4) Å (in av.), respectively, are typical of high-spin Mn­(II) systems. In fact, the unintentional occurrence of the water molecule in this structure proved quite fortunate, as the Mn–O_H2O_ bond length of 2.191(4) Å in this compound provided us with an excellent point of reference for comparison with the Mn–O bonds in Mn­(IV)=O and Mn­(III)–OH complexes **9** and **10**, respectively (*vide infra*).

**Figure 4:**
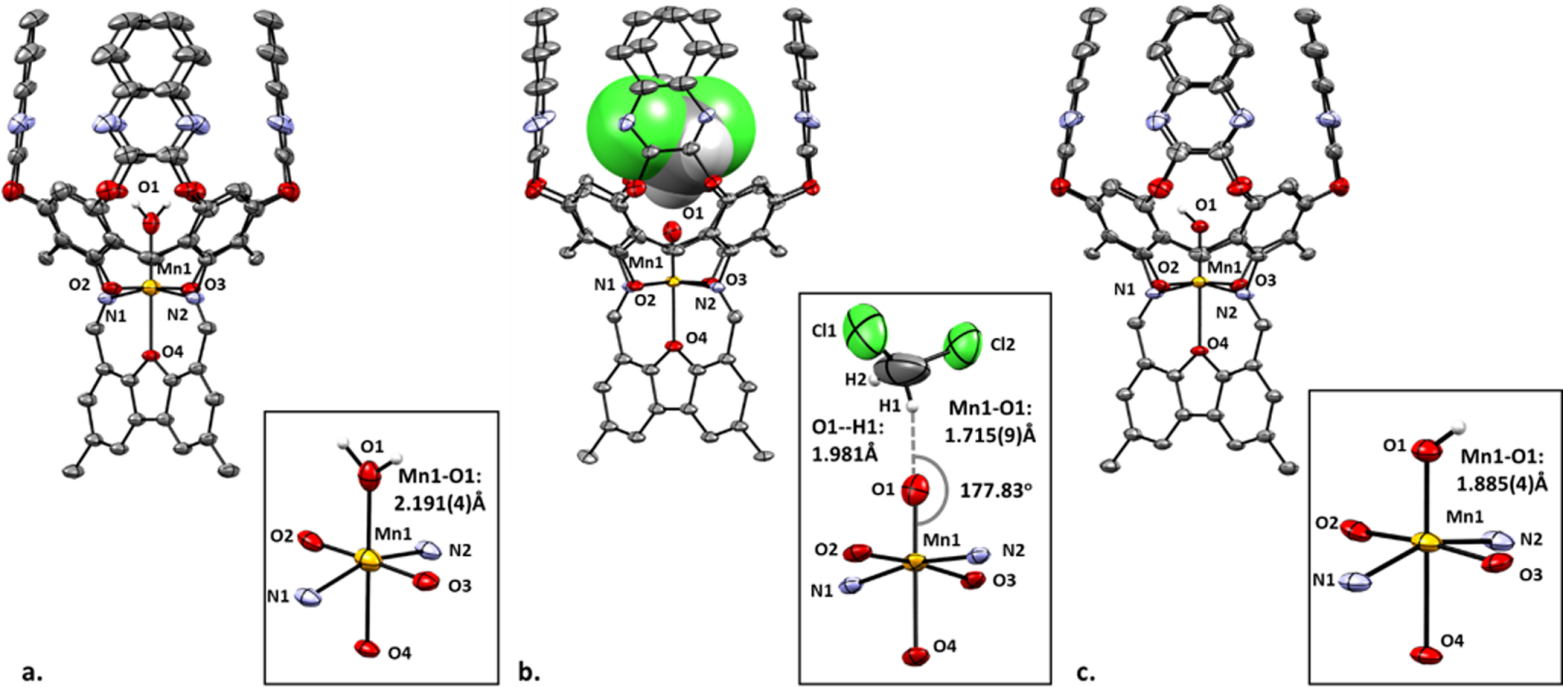
XRD structures of **8**·H_2_O (a), **9** (b), and **10** (c). In the case of **9**, the DCM molecule trapped within the cavitand is shown in the spacefill mode. The bottom image in each case depicts a close-up of the metal’s primary coordination sphere. All hydrogen atoms belonging to the cavitand structure, along with the two *n*-butyl groups at the lower rim, were omitted for clarity.

### Formation and Decay of a Mn­(IV)-Oxo Species within the Metallocavitand Framework

With fully characterized Mn­(II)-metallocavitand **8** in hand, we set out to explore the potential of this new ligand platform in stabilizing a monomeric Mn­(IV)-oxo species.

Adding a DCM solution of excess 1-(*tert*-butylsulfonyl)-2-iodosylbenzene (sPhIO)^76^ to a solution of **8** in the same solvent resulted in an immediate color change from pinkish orange to wine-red (Figure 5a, top left and top right, respectively). A similar, albeit slower, color change occurred upon stirring the DCM solution of **8** with solid iodosyl benzene (PhIO). In both cases, ESI-HRMS detected a molecular ion mass of [M]^+^ = 1423.3147 amu, matching the empirical formula of **8** plus an O atom, suggesting the likely formation of a monomeric Mn-oxo species. Furthermore, the Evans method measurements of the reaction solution, taken before and after the addition of sPhIO, revealed a sharp decrease of the effective magnetic moment from 5.58 μ_B_ to 3.88 μ_B_, the latter exactly matching the theoretical value for an *S* = 3/2 system. This finding was consistent with a quantitative transformation of the high-spin *S* = 5/2 Mn­(II) complex into the corresponding Mn­(IV)-oxo species.

**Figure 5:**
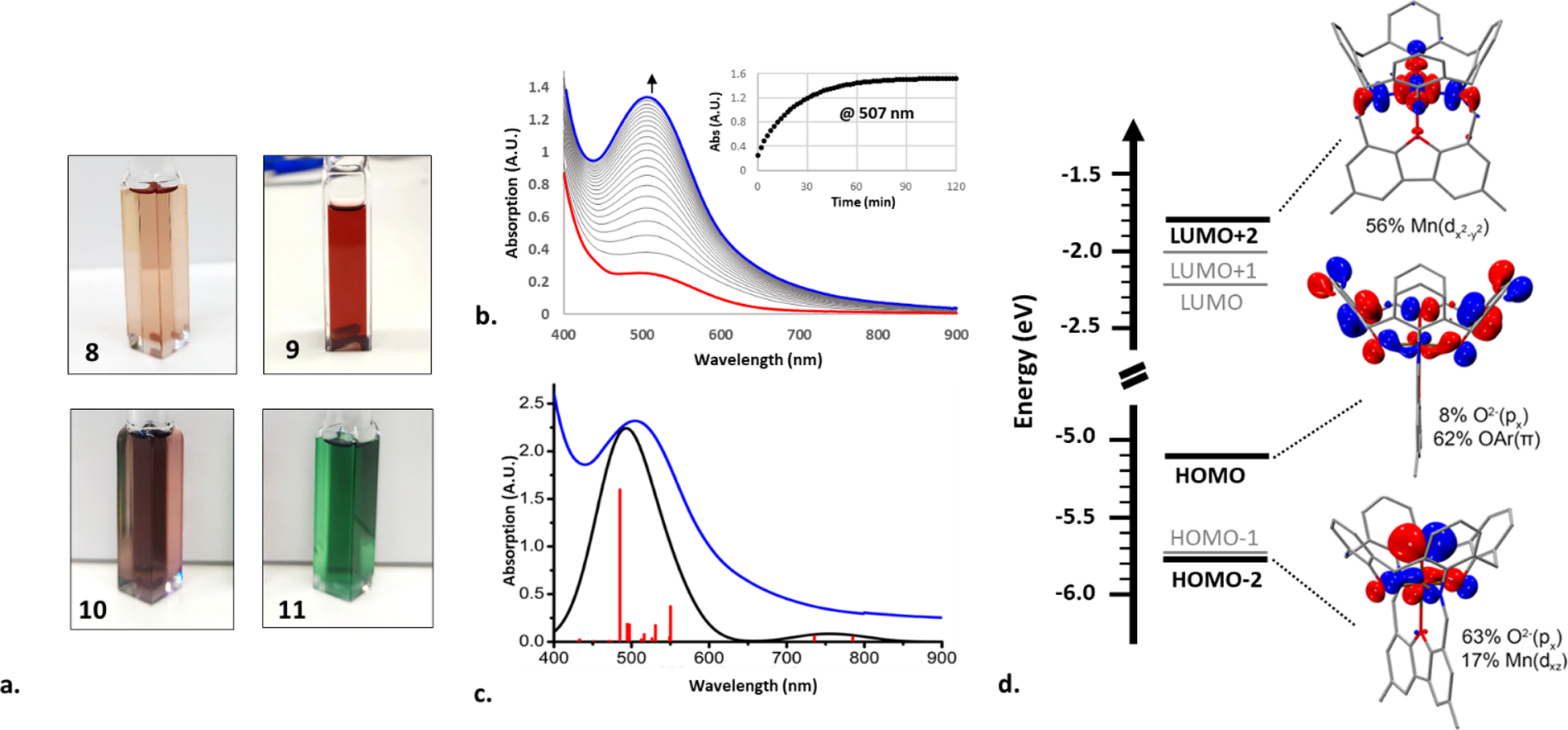
(a) 0.6 mM solutions of complexes **8** and **9** in anhydrous DCM and complexes **10** and **11** in *o*-difluorobenzene (DFB). (b) Spectral changes in the UV–vis absorption of **8** upon the addition of 1.5 equiv of sPhIO in DCM at −20 °C. Inset: the change over time at λ_max_ = 507 nm. (c) The absorption spectrum of the model complex 9* predicted by TDDFT (red bars: relative oscillator strengths; black trace: calculated spectrum with peak-broadening; blue trace: the experimental spectrum). (d) Molecular orbitals involved in the predicted absorption band at 485 nm.

Yet, such an oxidation state assignment of complex **9** required further scrutiny, as the observed total spin of *S* = 3/2 is not unique to a Mn­(*d* ^3^) system with 3 metal-based unpaired electrons (Mn­(IV)-oxo), but could also arise from a Mn­(*d* ^4^)-O­(*p* ^1^) system (Mn­(III)-oxyl), where the oxygen radical is antiferromagnetically coupled to one of the Mn­(*d*) electrons.

To rule out the latter possibility, the oxidation state of the metal center in **9** was probed by X-ray photoelectric spectroscopy (XPS) and compared to parent Mn­(II) complex **8**, as well as to Mn­(III) complexes **10** and **11**, described later in the text. The obtained Mn 2p spectra exhibited two main peaks, with a characteristic 3:1 ratio, corresponding to the 2p 3/2 and 2p 1/2 primary photoelectron lines (Table S1 and Figure S28a–d). For complex **9**, the observed peaks at 642.20 and 654.20 eV were in excellent agreement with the Mn 2p electron binding energies reported for other Mn­(IV) systems.^77,78^ These values were positively shifted not only relative to those observed for complex **8** (Δ*E*_av_ = 1.35 eV) but also, more importantly, relative to the two Mn­(III) complexes (Δ*E*_av_ = 0.69 eV), strongly supporting the Mn­(IV)-oxo rather the Mn­(III)-oxyl assignment for complex **9**.

Monitoring the formation of **9** by UV–vis spectroscopy at −20 °C (Figure 5b) showed a gradual increase in absorption at a prominent band centered at λ_max_ = 507 nm. This finding was somewhat surprising, as characteristic absorption bands of the reported Mn­(IV)-oxo species typically occur at significantly longer wavelengths (700–1000 nm).^12,14,15,21,22^ Therefore, to gain insight into the origin of this unexpected absorption band, we performed a time-dependent DFT (TDDFT) study on model Mn­(IV)-oxo complex 9*, simplified similarly to 8* (see SI for details). The computational results indeed predicted an electronic transition with the biggest oscillatory strength to occur at 485 nm (Figure 5c), in good agreement with the observed spectrum. A detailed examination of the electronic states involved in this transition identified its two major constituents as HOMO­(α) → LUMO+2­(α) (60%) and HOMO–2­(α) → LUMO+2­(α) (24%). Inspection of the corresponding molecular orbitals (Figure 5d) revealed that, while the LUMO+2­(α) acceptor orbital is an antibonding σ* orbital, with a predominantly metal character (56% Mn *d*_*x* ^2^–*y* ^2^_), only the HOMO–2­(α) donor orbital has a significant contribution from the metal-oxo moiety (17% Mn *d*_*xz*_ and 63% O_oxo_ *p*_*z*_).

The other donor orbital, HOMO­(α), which is the major contributor to the transition, is predominantly localized on the phenolate rings of the cavitand (exhibiting a π* antibonding character) and has only a minor contribution from the metal-oxo moiety. Thus, the electronic transition that gives rise to the absorption at 507 nm can best be described as a ligand-to-metal charge transfer (LMCT). This assignment is also supported by the high intensity of this absorption band (ε = 2,600 L·mol^–1^cm^–1^) in accordance with other metal-phenolate complexes.^79,80^ In fact, two lower energy Laporte- forbidden *d*–*d* transitions (at 735 and 785 nm), similar to the reported Mn­(IV)-oxos,^11,12,81^ were also predicted by our TDDFT calculations. However, due to their significantly lower intensities compared to the LMCT transition, the corresponding absorption bands could not be resolved in the spectrum.

The absence of high intensity absorption bands in the near-infrared (NIR) region in the spectrum of **9** provides yet another bit of crucial information as to its electronic structure. Previous spectroscopic studies on metal-coordinated phenoxy radicals have established that these complexes exhibit an intense intraligand charge transfer (ILCT) band in this region of the spectrum.^80,82,83^ The fact that no such band was observed rules out spin density delocalization over the phenolate moieties of the cavitand ligand, validating the formal description of **9** as a Mn­(IV) complex, rather than a Mn­(III)-phenoxy radical species. Indeed, the Mulliken population analysis showed the spin density in 9* to be localized on the metal-oxo unit (primarily on the Mn center, with a minor contribution from the O_oxo_ atom) and to have a negligible spin delocalization over the phenolic oxygens (Figure 6a). Thus, remarkably, despite containing the easily oxidizable phenol groups, the cavitand behaves as a redox-innocent ligand framework.

**Figure 6:**
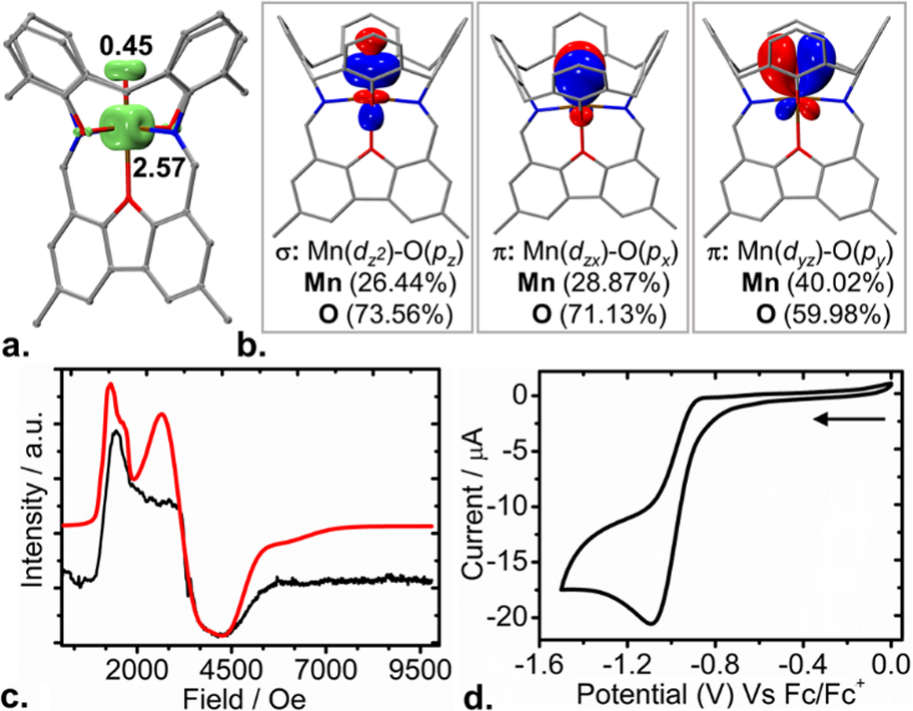
(a) Spin density distribution and (b) natural bond orbitals (NBOs) localized on the MnO fragment, calculated for model complex 9*; (c) frozen DCM solution X-band CW-EPR spectrum of **9** at 5 K (black trace), overlaid with the simulated spectrum (red trace); (d) cyclic voltammogram of **9** (0.25 mM) under a N_2_ atmosphere in DCM at 0.1 V/s at rt.

Complex **9** was further characterized by an X-BAND EPR measurement carried out on its freshly prepared DCM solution frozen to 5 K. Similarly to several other *S* = 3/2 Mn­(IV) complexes,^81,84,85^ the recorded EPR spectrum of **9** featured a narrow peak in the low field region (1400 Oe) and a very broad one in the high field region (4200 Oe) (Figure 6c, black trace). Simulation of the experimental data for an *S* = 3/2 system with the spin Hamiltonian parameters given in Table 1 (2nd row) resulted in reasonable agreement with the recorded spectrum (Figure 6c, red trace). In particular, the simulated axial ZFS value (*D* = −0.914 cm^–1^) occurs within the range typical for pseudo-octahedral N,O-coordinated Mn­(IV) species (*D* = |0.67| – |2.5| cm^–1^).^84,86^ Due to the large broadening of the EPR signal, well-resolved hyperfine splitting with the ^55^Mn nucleus could not be observed (a well-known phenomenon for Mn­(IV) species).^87^ Nonetheless, to simulate the experimental spectrum, we considered nearly axial hyperfine coupling constants (Table 1, second row, last column). The corresponding **A**_**iso**_ = 248 MHz value is in good agreement with the literature.^84^

**Table 1:** Simulated and Calculated (CASSCF/NEVPT2) Spin Hamiltonian Parameters for Complexes **9** and 9*, Respectively

		*D* (cm^–1^) (*D* Strain)	*E*/*D*	g_1_, g_2_, g_3_ (A1, A2, A3) (MHz)
Simulated from the EPR spectrum		–0.914 (0.15, 0.067)	0.33	2.00, 1.98, 1.70 (200, 300, 245)
Calculated (CASSCF/NEVPT2)	CAS(3,5)	–1.04	0.07	1.94, 1.96, 1.97
	CAS(11,9)	–1.41	0.22	1.97, 1.99, 1.99

To verify the correspondence of the recorded EPR spectrum with the structure of **9**, the simulated spin Hamiltonian parameters were corroborated by ab initio/CASSCF/NEVPT2 calculations on the model complex 9*. Since spin Hamiltonian parameters are well-known to be highly sensitive to the size of the active space and the number of electronic states used in such calculations,^88^ several different active space/electronic state combinations were employed (see Table S7 for detailed results). The minimal CAS­(3,5) active space, considered to be only the Mn 3*d* orbitals (Figure S70), yielded a close axial ZFS value (*D*) but failed to correctly predict the observed rhombicity value (*E*/*D*, Table 1, third row). A better agreement with both axial and rhombic experimental ZFS parameters (Table 1, fourth row) was obtained with a larger CAS­(11,9) active space which takes into account all metal- and ligand-based orbitals contributing to the metal’s first coordination sphere (Figure S71).

In addition to providing a theoretical justification for the simulated EPR spectrum of **9**, the above CASSCF calculations also further validated the formal description of its core as a Mn­(IV)-oxo species. In full agreement with our XPS studies, the Mn­(IV)-oxo form was found to be the predominating ground state electronic configuration (72%), while the relative contribution of a Mn­(III)-oxyl radical was negligibly small (4%) (Figure S72). A similar situation occurs also in the pseudo-octahedral Mn­(IV)-oxo complexes of the poly pyridyl ligands (NPy4), extensively studied by Jackson.^89^

An XRD-quality single crystal of complex **9** was grown at −30 °C from an anhydrous DCM solution under rigorously air-free conditions (nitrogen glovebox). The obtained structure (Figure 4b) clearly shows the presence of a metal-coordinated oxo ligand located within the interior of the cavity and facing an entrapped DCM molecule. However, unlike in complex **8**·H_2_O, here, the DCM molecule in the cavity acquires a single well-defined spatial orientation due to a strong H-bond between the oxo-moiety and one of its methylene protons (CH--O: 1.981 Å, Figure 4b inset). Accordingly, the Mn–O bond length of 1.715(9) Å was slightly longer than that of the pseudotetrahedral Mn­(IV)-oxo species reported by Nocera (1.673 Å)^37^ but in an excellent agreement with the corresponding bond lengths of other pseudo-octahedral Mn­(IV)-oxos, stabilized by H-bonding or Brønsted/Lewis acids, as inferred from EXAFS measurements and predicted computationally.^12,14,22,90^

Despite the Mn–O bond in **9** being slightly more elongated than expected, a strong indication of a bond order of two within this metal-oxo unit was obtained from natural bond orbital (NBO) calculations, performed on the aforementioned model complex 9*. These clearly revealed three singly occupied bonding orbitals, corresponding to one σ- and two π-bonds, localized on the MnO fragment (Figure 6b). The calculated Wiberg bond index of 1.57 was consistent with a double bond character and identical with that previously calculated by Rajaraman for Borovik’s hydrogen-bonded Mn­(IV)-oxo system **IV** (Figure 1a).^91^

In addition, cyclic voltammetry (CV) of complex **9**, measured in anhydrous DCM, showed an irreversible reduction wave at −1.0 V (Fc/Fc^+^). This value exactly matches the Mn­(IV)-oxo/Mn­(III)-oxo redox couple reported for Borovik’s system (complex **IV**, Figure 1a)^92^ but is shifted anodically relative to another non-hydrogen-bonded system (complex **II**, Figure 1a, −0.38 V).^22^ Comparable redox potential shifts between H-bonded and non-H-bonded were also reported for Fe­(IV)-oxo systems.^93^ This suggests that, similar to complex **IV**, the low redox potential of complex **9** may arise from stabilizing H-bonding interactions, which in this case are provided by DCM molecules (as evident from the corresponding XRD structure).

The fact that XRD quality crystals of **9** could be grown from its DCM solution was quite surprising, given that this highly reactive species decays rapidly in that solvent at rt, even when kept under strictly anhydrous and air-free conditions (Figure 7a). This observation is in line with other Mn­(IV)-oxo systems found in the literature, as most reported pseudo-octahedral Mn­(IV)-oxo intermediates have short lifetimes in solution, ranging from several minutes to several hours.^12,90^ However, while in other metal-oxo systems this decay occurs via the formation of μ-oxo-bridged dimers,^12,15,94^ the bulky cavitand framework of complex **9** renders such a dimerization impossible, suggesting a different process to take place here.

**Figure 7:**
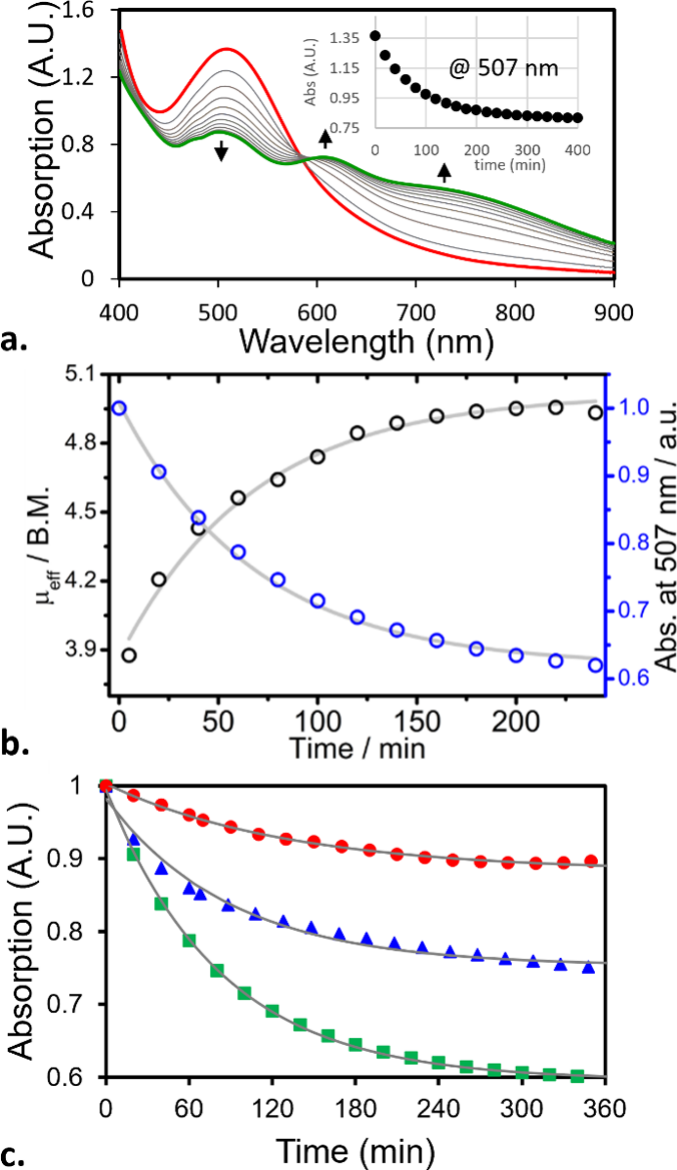
(a) Change in electronic absorption of **9** in anhydrous DCM at rt over the course of 7.5 h (black arrows indicate change in direction). Inset: change in absorption at λ_max_ = 507 nm vs time. (b) Change in absorption at λ_max_ (blue circles) and effective magnetic moment (black circles) due to the HAA reaction of **9** with DCM. (c) Decay of **9** (0.6 mM) observed via change of absorption at 507 nm in DCE (red circles), CHCl_3_ (blue triangles), and DCM (green squares). Gray traces indicate best 1st order rate fits.

The insight into the nature of the decay of **9** in solution came serendipitously when a single crystal of the Mn-containing product **10** was isolated from the solution of complex **9** used for tracing its decay by UV–vis spectroscopy. The XRD structure of this complex (Figure 4c) is very similar to those of **8**·H_2_O and **9**. Yet, the Mn–O bond length of 1.885(4) Å in it lies between those of the Mn­(II)-(OH_2_) and the Mn­(IV)-oxo species and was consistent with the values reported for typical monomeric Mn­(III)–OH complexes (1.80–1.90 Å).^35,95^ The (+3) oxidation state assignment of this EPR-silent complex was further supported by the Evans method, which yielded μ_eff_ = 4.74 μ_B_. This value is very close to the theoretical spin-only magnetic moment of 4.89 μ_B_ for a high spin *d* ^4^ system and falls within the range reported for other Mn­(III) complexes (4.7–5.2 μ_B_).^72^ Accordingly, the Mn 2*p* photoelectron peaks at 641.55 and 653.65 eV observed for this species (Figure S28c) lie in between the values measured for the Mn­(II) and Mn­(IV) centers in complexes **8** and **9**, respectively (Table S1), and are consistent with the typical 2p_3/2_ and 2p_1/2_ values reported for Mn­(III) species.^78^

Thus, it appeared that the Mn­(IV)-oxo core of **9** decays via a HAA reaction with the solvent molecules (DCM) (small enough to fit in the cavity) being the most likely HA donors. Indeed, the close approach of the DCM molecule, observed in the XRD structure of **9**, might suggest a similar geometry to that of the corresponding transition state (*vide infra*).

To confirm that the Mn­(IV)-oxo species **9** decays predominantly through the HAA mechanism, we measured its decay kinetics by tracking the total effective magnetic moment of the solution in anhydrous DCM and correlated it with the corresponding changes observed in the electronic absorption (Figure 7b). The effective magnetic moment was found to increase during the decay from 3.88 μ_B_ (immediately after the addition of sPhIO to complex **8**) to 4.91 μ_B_ over the course of 4 h, fully consistent with the transformation of a *d* ^3^ into a *d* ^4^ system.^37,96^ This one-electron reduction appears to follow first order kinetics with a half-life of *t*_1/2_ = 47 ± 2 min. A very similar kinetic profile and half-life (*t*_1/2_ = 50 ± 1 min) was also obtained by tracing the decrease of the absorption band centered at λ_max_ = 507 nm, strongly suggesting that both measurements reflected the same process, i.e., the HAA from DCM by complex **9**.

Finally, we attempted to obtain complex **10** directly, by reacting complex **8** with an excess of ^t^BuOOH, a hydroxyl radical source (Figure 8a). Surprisingly, upon its addition, the solution of **8** immediately turned emerald-green (Figure 5a, bottom right), displaying a distinct absorption pattern different from the one we obtained for the decay product(s) of complex **9** (Figure 8b, plots B vs A).

**Figure 8:**
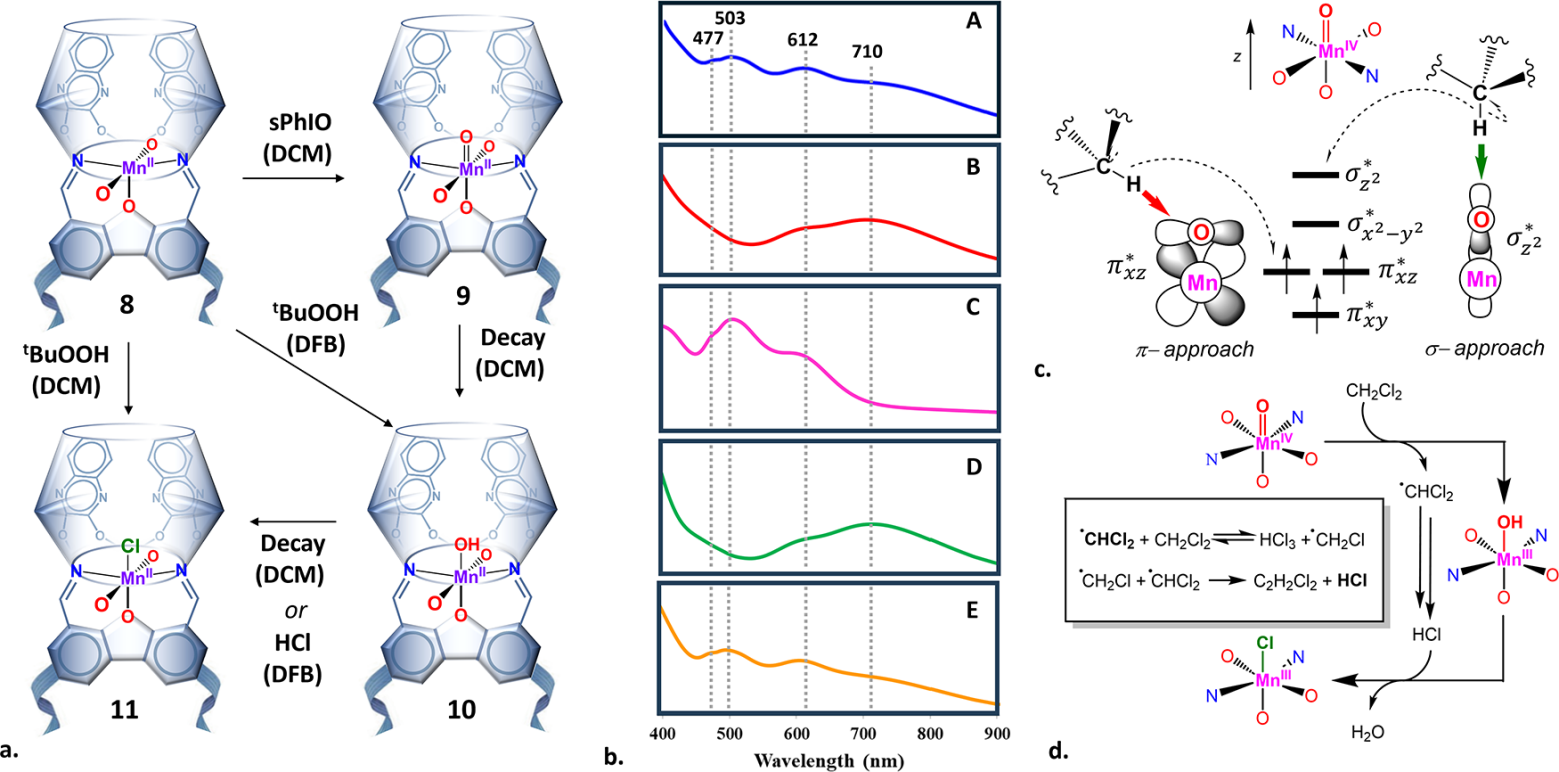
(a) The oxidation of **8** upon reaction with either sPhIO or ^t^BuOOH in DCM or DFB and the suggested pathway for the complete decay of **9** in DCM. (b) Electronic visible absorption spectra of the mixture of decay products of **9** in DCM (plot A), the product of reacting **8** with ^t^BuOOH in DCM (plot B), the product of the same reaction in DFB (plot C), the same product shown in plot C after addition of excess anhydrous HCl (plot D), and a 1:1 superposition of plots C and D (plot E). (c) Suggested mechanism for the complete decay of **9** in DCM. Inset: the pathway suggested by Hassler^97^ for the formation of HCl upon radical degradation of DCM. (d) Schematic representation of the HAA reaction between a Mn­(IV)-oxo moiety and a C–H bond, proceding via π- or σ-approach pathways.

A molecular ion of mass of [M]^+^ = 1442.2886 amu, consistent with the empirical formula and isotope pattern of a Mn­(III)-Cl complex (**11**), was detected by ESI-HRMS (Figure S82), while the trivalent state of the Mn center in this complex could be deduced both from its magnetic moment (μ_eff_ = 4.61 μ_B_) and from the Mn 2p electron binding energies, close to those of **10** (Table S1 and Figure S28d). In fact, the same compound also gradually formed when the decayed samples of **9** in DCM were kept in airtight cuvettes for several days, as their color gradually changed from purple-brown to green.

Since the only possible chloride source in this system could have been DCM, we repeated the reaction of **8** with ^t^BuOOH in *o*-difluorobenzene (DFB), a nonchlorinated solvent. This time, the color of the obtained solution visually resembled that produced by the decay of **9** in DCM (Figure 5a, bottom left). Yet, while sharing with the latter the absorption peaks at 477, 503, and 612 nm, its spectrum lacked the broad shoulder in the 700–900 nm region (Figure 8b, plots C vs A). Treating the same DFB solution with an excess of anhydrous HCl resulted in an immediate change in color from brown to green. The corresponding spectrum exhibited a very broad peak centered at λ_max_ = 710 nm, as observed for **11**, while the characteristic peaks of **10** at 477 and 503 nm disappeared (Figure 8b, plots D vs B). Furthermore, a 1:1 superposition of the absorption spectra of both DFB solutions (i.e., before and after the addition of HCl) yielded a spectrum nearly identical with that obtained in the initial decay of **9** in DCM (Figure 8b, plots E vs A). Thus, the observed decay of the Mn­(IV)-oxo species in DCM produces two different Mn­(III) species, **10** and **11**, as shown in Figure 8a.

In fact, the formation of complex **11** provides an additional indication of the HAA taking place between the Mn­(IV)-oxo species and DCM, as it explains what happens to the dichloromethyl radical formed in this reaction. Previous studies on mono- and dichloromethyl radicals generated *in situ* from DCM in the gas phase have shown that these highly reactive species tend to couple, especially at high pressures, with the elimination of HCl (Figure 8c, inset).^97^ If a similar process occurs in solution as well, one would expect the decay of **9** by HAA to result in the accumulation of HCl in the reaction medium. This in turn would result in the protonation of the terminal hydroxy ligand of **10**, followed by the chloride ion displacing the resulting water molecule, gradually converting **10** into **11** (Figure 8c). Presumably, the same process occurs even more rapidly in the presence of excess ^t^BuOOH.

It is generally accepted that the HAA reaction represents the first and rate-determining step in the C–H activation by Mn­(IV)-oxos,^5^ which is also true for Fe­(IV)-oxo species (operating via the rebound mechanism).^6^ However, whereas in the case of Fe-oxos this step has been shown to progress via either σ or π pathways (with the latter being more favored kinetically), no experimental evidence for the feasibility of a σ-approach in the case of Mn­(IV)-oxo has been presented to date. In fact, it has been postulated that in a Mn­(IV)-oxo system such a pathway is inaccessible due to the high energy of its *d*_z^2^_ orbital compared to an Fe-based congener (Figure 8d).^4^ In light of the above, the XRD structure of complex **9** appears even more intriguing, as it shows the DCM molecule at a suitable distance (O1–H1: 1.981 Å) and angle (Mn1–O1–H1: 177.83°) for the HAA to proceed via the σ-pathway (Figure 4b, inset). This raises the possibility that, despite being energetically unfavorable for typical Mn­(IV)-oxo species, this reaction pathway may still occur in the cavitand system, being driven by the interaction of the metal-oxo center with the secondary coordination sphere (provided by the macrocyclic ligand).

Further insight into the HAA reactivity of complex **9** was gained by monitoring its decay in different chlorinated solvents (Figures S29–S31 and S36–S38). While all observed decomposition profiles followed first order kinetics (Figure 7c), the decay half-lives (Table 2, column 6) did not show any clear correlation with either the number of C–H bonds per solvent molecule or the corresponding homolytic bond dissociation energy (BDE) values (Table 2, columns 3 and 4). In fact, complex **9** was found to be significantly more stable in DCE, compared to both DCM and CHCl_3_, despite having the highest number of C–H bonds per molecule and a C–H BDE between (and very similar to) the corresponding values of the other two solvents.

**Table 2:** Half-Lives of Mn­(IV)-Oxo Species **9** in Chlorinated Solvents Compared to Their Corresponding C–H BDEs, Number of Molecular C–H Bonds, and Approximate VDW Volumes

Entry	Solvent	C–H BDE (kcal/mol)^98^	Reactive C–H Bonds per Molecule	VDW Volume (Å^3^)	*t*_1/2_ (min)
1	DCM	97.3 ± 1.0	2	56.6^b^	50 ± 1
2	CHCl_3_	93.8 ± 0.6	1	70.7^b^	62 ± 5
3	1,2- DCE	95.3 ± 2.3^a^	4	73.6^b^	186 ± 6

a The value shown for the C–H BDE of 1,2-DCE is lacking in the literature and has thus been estimated using the literary BDE values for chloroethane and 1,1-DCE as lower and upper bounds, correspondingly.

b The approximate VDW molecular volumes were calculated as suggested by Bondi.^99^

On the other hand, an interesting trend between the decay half-lives and the spatial characteristics of the solvent molecules was noted. The *t*_1/2_ values measured in DCM and CHCl_3_ seem proportional to their approximate VDW molecular volumes (Table 2, column 5). In DCE, however, the decay rate is ca. 3 times slower than what might be expected based on its molecular volume, being only slightly larger than that of CHCl_3_.

Yet, while DCM and CHCl_3_ are roughly spherical molecules, the elongated shape of the DCE molecule imposes an additional limitation on its HAA reaction with the Mn­(IV)-oxo core of **9**. When in the most common *anti* conformation, the longest dimension of the DCE molecule (*d*_VDW_ = 7.9 Å) is nearly twice as long as the average diameter of the cavity aperture defined by its aromatic walls (*d*_VDW_ = 3.9 Å). As a result, to enter the cavity for the reaction with the Mn­(IV)-oxo moiety, the DCE molecule must be aligned along its longest axis. This limits its possible spatial orientations to just one out of three (i.e., along the *x*, *y*, and *z* axes), which might explain why the HAA reaction with DCE occurs approximately 3 times slower than could be expected based on its molecular volume alone.

### Reactivity of Complex **9** with HA Donors and OA Acceptors

Following the decay studies in chlorinated solvents, we wondered whether a similar size/shape selectivity of complex **9** can also be observed in reactions with hydrocarbon substrates, which are known to undergo HAA by Mn­(IV)-oxo complexes. Specifically, the following series of compounds, differing in their steric bulk and C–H BDE, was tested: toluene (Tol), ethylbenzene (EB), *p*-cymene (pCy), diphenylmethane (DPM), cyclohexene (CH), 1,4-cyclohexadiene (CHD), and 9,10-dihydroanthracene (DHA). An effective rate constant for each of these substrates was measured under pseudo first order conditions and normalized according to the number of reactive C–H bonds in the molecule (Table 3). Since complex **9** was found to be most stable in DCE (Figure 7c), this solvent was chosen as the reaction medium for these studies.

**Table 3:** Normalized HAA Rate Constants by Compound **9** for a Series of Hydrocarbon Substrates, Compared to Their C–H BDEs and the Number of Reactive C–H Bonds

Entry	Substrate	C–H/D BDE (kcal/mol)^98^	Reactive C–H/D Bonds per Molecule	*k*_norm_ (s^–1^)
1	CHD	76.0	4	(2.17 ± 0.01) × 10^–1^
2	DHA	76.3	4	(1.68 ± 0.01) × 10^–2^
3	DHA-*d*_4_	77.45^a^	4	(7.25 ± 0.01) × 10^–3^
4	pCy	83.5	1	(1.18 ± 0.01) × 10^–2^
5	DPM	84.5	2	(2.85 ± 0.04) × 10^–6^
6	EB	87.0	2	(1.35 ± 0.05) × 10^–2^
7	CH	87.0	4	(4.75 ± 0.25) × 10^–4^
8	Tol	89.7	3	(1.23 ± 0.04) × 10^–6^

a The BDE_C‑D_ value for DHA-*d*_4_ was approximated from an average vibration frequency difference between C–H and C–D bonds (Δν = 800 cm^–1^), which yields BDE_C‑D_ = BDE_C–H_ + 1.15 kcal/mol.^100^

As evident from the diagram in Figure 9, the HAA reactivity of complex **9** varies dramatically with substrates that have similar C–H bond BDEs but markedly different molecular sizes. For example, the smaller CHD reacts an order of magnitude faster than the larger DHA, despite their nearly identical C–H BDEs (Table 3, entries 1 and 2). Moreover, while the benzylic C–H bond in pCy is only 1 kcal/mol weaker than those in DPM, owing to its smaller and more compact shape, pCy reacts with **9** 4 orders of magnitude faster (Table 3, entries 4 and 5). On the other hand, EB, which shares a similar molecular shape with pCy, exhibits a comparable rate constant despite a larger difference between the secondary and tertiary benzylic C–H bond(s) in these molecules (Table 3, entries 4 and 6).

**Figure 9:**
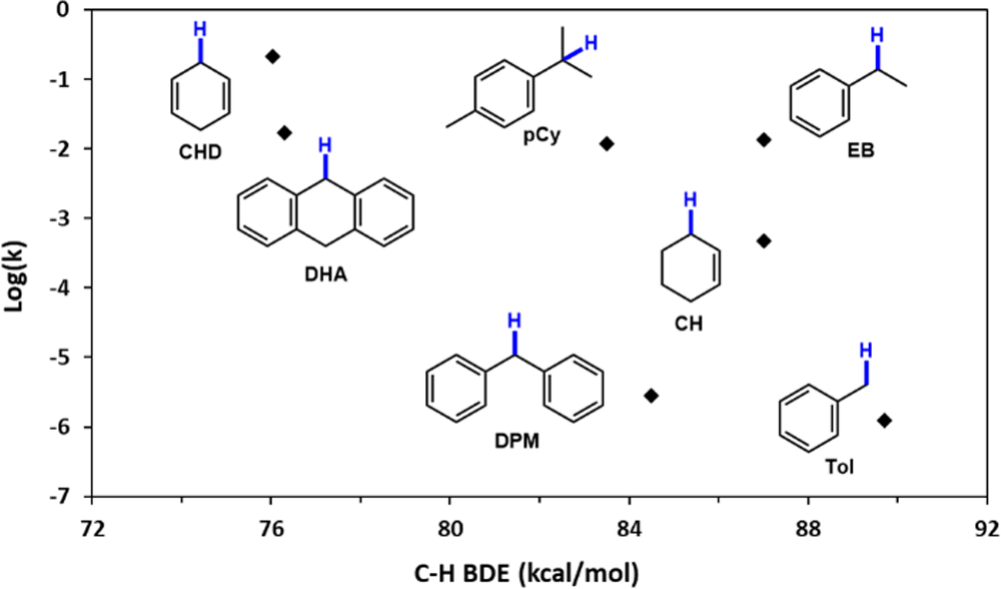
Normalized HAA rate constants under pseudo 1st order conditions vs BDE values of the corresponding C–H bonds.

Naturally, the variation in HAA reactivity of **9** is not merely due to the steric factors but also affected by the C–H BDE of the substrates. Thus, CH and CHD molecules are nearly identical in shape, yet the ∼10 kcal/mol difference in the BDE between allylic vs bis-allylic C–H bonds in these substrates results in a 3 orders of magnitude difference in their reactivity (Table 3, entries 1 and 7). Similarly, the strong primary benzylic C–H bonds in Tol yield the smallest rate constant despite its compact size (Table 3, entry 8).

Finally, to confirm the HAA mechanism, we determined the kinetic isotope effect (KIE) for the reaction with DHA, using its 9,9,10,10-tetradedeuterated isotopolog (DHA-*d*_4_). The observed KIE of 2.3(2) supports that homolytic C–H bond cleavage constitutes the rate limiting step in this process and is consistent with a “classical” HAA mechanism, rather than with one involving quantum tunneling (which typically results in significantly higher KIE values).^17^ Similar KIE values have previously been reported for the oxidation of DHA (KIE = 3.78)^101^ and xanthene (KIE = 3.4)^81^ with monomeric Mn­(IV)-oxo systems.

The steric substrate control of complex **9** was not limited to HAA, but was also observed for the reactivity of the OAT with different tertiary phosphines. Specifically, when reacted with a 1:1 mixture of the small trimethylphosphine (PMe_3_) and the bulkier tricyclohexylphosphine (PCy_3_), the oxo-moiety of **9** was fully transferred to the former within minutes, while leaving the latter unaffected, as confirmed by a quantitative ^31^P NMR measurement (Figure 10a). Interestingly, while the ^31^P NMR signal of PCy_3_ remained sharp, the signals of both PMe_3_ and the oxide Me_3_P­(O) showed a significant broadening, presumably due to the labile interaction of these small molecules with the coordinatively unsaturated Mn­(II) center in the resulting complex **8**, the formation of which was evident by UV–vis spectroscopy (Figure S35).

**Figure 10:**
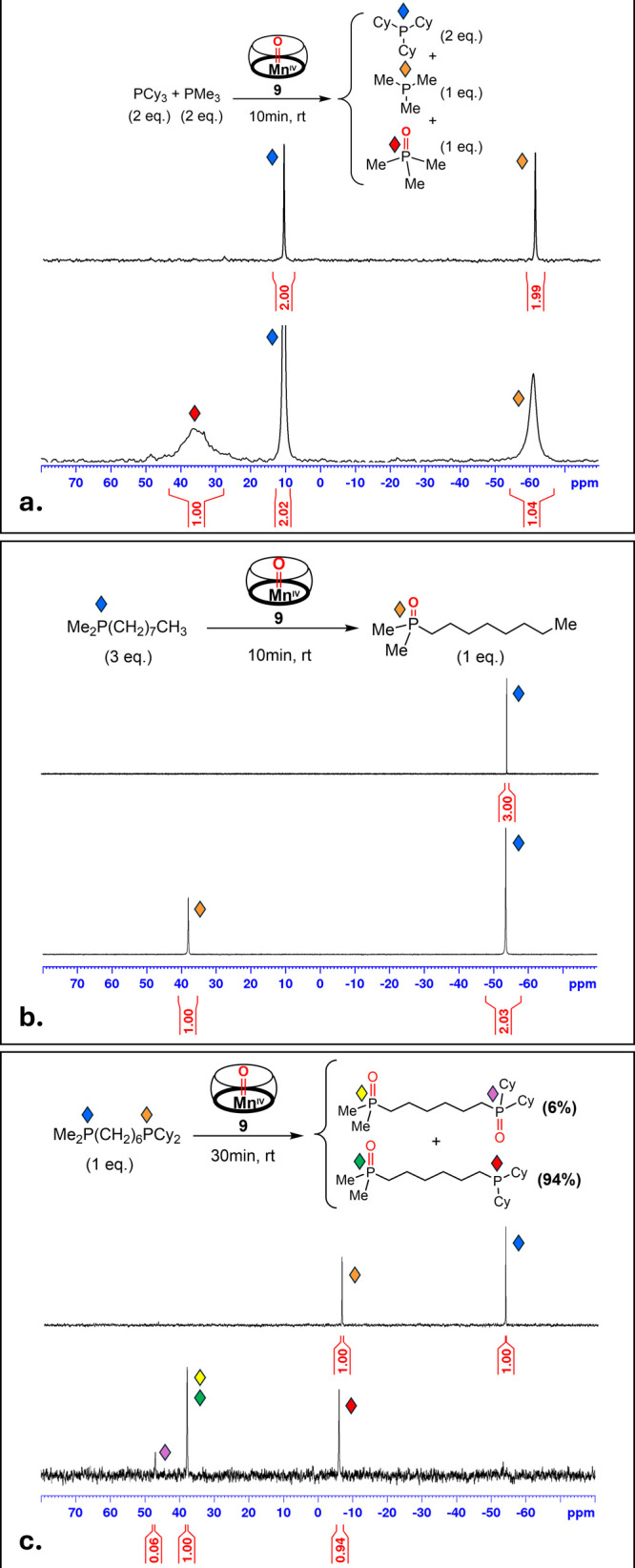
^31^P NMR spectra in DCE of (a) a 1:1 mixture of PMe_3_ and PCy_3_, (b) dimethyloctylphosphine (**12**), and (c) asymmetric bisphosphine **13** before (top) and after (bottom) the addition of the Mn­(IV)-oxo species (**9**). The molar equivalents specified are with respect to compound **9**.

Unlike tricyclohexylphosphine, a long-chain dimethyloctylphosphine (**12**)^102^ did undergo full conversion into the corresponding phosphine oxide upon reaction with **9**, as confirmed by another quantitative ^31^P NMR measurement (Figure 10b). This proved that large substrate molecules are still able to react with **9**, provided that their shape allows access to the reactive metal-oxo core within its cavity.

Lastly, we were able to demonstrate that this cavitand-imposed shape selectivity can be translated into a highly regioselective OAT process. For this purpose, **9** was treated with an equimolar amount of asymmetric linear bisphosphine **13**, featuring two phosphine groups of significantly differing steric bulk (PMe_2_ vs PCy_2_) at its termini.

In this case, the dimethylphosphine terminus, small enough to enter the cavity, underwent oxidation, whereas the bulky dicyclohexylphosphine end remained largely untouched, with only 6% of the doubly oxidized product observed (Figure 10c). Complex **9** seems to be quite unique in this regioselectivity for OAT, as both MnO_2_ and sPhIO failed to show any discrimination between the two termini of **13** in control experiments (see Figures S25 and S26). Such a regioselective oxidation of asymmetric bisphosphines by conventional synthetic methods is highly challenging^103^ and is quite reminiscent of enzymatic systems, capable of performing organic transformations with high degrees of regioselectivity.^104^

## Summary and Conclusions

In this study, we reported the design and synthesis of a conceptually novel ligand architecture, exhibiting a rigid pentacoordinate metal-binding cage integrated within a cavitand scaffold. The corresponding Mn­(II) metallocavitand reacted with oxo-transfer reagents, generating a highly reactive terminal Mn­(IV)-oxo species deeply buried within its molecular cavity. The steric protection provided by the cavitand enabled a full characterization of this elusive species not only through various spectroscopic techniques (UV–vis, EPR, XPS, and HRMS) but also, for the first time for such a pseudo-octahedral Mn­(IV)-oxo complex, by means of XRD.

Remarkably, the obtained crystal structure also features an entrapped DCM molecule hydrogen-bonded to the metal-oxo moiety, positioned and orientated in a manner reminiscent of a putative transition state of an HAA reaction taking place via the σ-approach (that has so far been largely considered inaccessible to Mn­(IV)-oxo systems). Kinetic studies performed also support this assertion. Regardless of the exact mechanism, the HAA reaction was found to be the primary pathway for the decomposition of this Mn­(IV)-oxo complex in chlorinated solvents, which exhibited a notable rate dependence on the molecular dimensions of the solvent used. Accordingly, the cavitand framework was found to impose a high degree of size and shape selectivity on the Mn­(IV)-oxo center in its HAA and OAT reactions with weak C–H bond hydrocarbons and tertiary phosphines, respectively. This steric control could also be harnessed for a regioselective oxidation of an asymmetric bisphosphine substrate, a transformation that is considered highly challenging to accomplish in nonenzymatic systems.

Thus, the metallocavitand system presented herein marks a significant advancement toward supramolecular catalysts capable of mimicking natural metalloenzymes in terms of their high activity and precise substrate selectivity. Furthermore, we believe that this molecular platform holds great potential for the isolation, characterization, and detailed reactivity studies of other elusive metal-oxo and metal-nitrido species, an endeavor currently underway in our lab.

## Supplementary Material





## Notes

All experimental procedures, NMR, EPR, UV–vis, XPS, and HRMS spectra, crystallographic tables, and computational details (PDF)
XYZ coordinates of the optimized model compounds: 5*_DBF_, 5*_Xant_, 5*_Pyr_, 5*_NH2_, DBF-_CHO_, Xant-_CHO_, Pyr-_CHO_, 8*, and 9* (XYZ)
