# Supplementary material for: Formation and Reactivity of an Elusive Monomeric Mn(IV)-Oxo Species Inside a Cavitand Pore
Source: J Am Chem Soc. 2025 Aug 7;147(34):30647–60. doi: 10.1021/jacs.5c02637 (PMC12395487; doi:10.1021/jacs.5c02637)
Supplement: Supplementary file 1 [file ja5c02637_si_001.pdf]

Supporting Information

Formation and Reactivity of an Elusive Monomeric Mn(IV)-Oxo Species Inside a Cavitand Pore

Galon Green,[a] Kamal Uddin Ansari,[a] Thejasree Munikrishna,[a] Sagi Ezov,[a] Donia Shamali,[a] Laxmi Narayan Nanda,[a] Vitaly Gutkin,[a] Orit Cohen,[a], Daphna Shimon [a,*], and Yuri Tulchinsky [a,*]

[a]Institute of Chemistry, The Hebrew University of Jerusalem, Jerusalem, 9190401, Israel.

[*]Corresponding author

Table of Contents

1. General Information **S1**

2. Computational details **S2**

3. Synthetic procedures **S4**

3.1. Preparation of the cavitand **S4**

Triol[4]arene (1): **S4**

Bis(trifluoromethanesulfonate)octamethoxytriol[4]arene (2): **S5**

(Dibenzylamino)octamethoxytriol[4]arene (3): **S6**

(Diamino)octamethoxytriol[4]arene (4):........ S7

Bicyclic octamethoxytriol[4]arene 5: **S8**

Bicyclic decahydroxytriol[4]arene 6: **S9**

Cavitand 7: **S10**

3.2. Preparation of the metallocavitand complexes **S11**

Mn(II) metalocavitand (8) **S11**

Mn(III)-OH metallocavitand (10) **S12**

Mn(III)-Cl metallocavitand (11) **S13**

In-situ generation of the Mn(IV)=O intermediate (9) **S13**

3.3. Preparation of an asymmetric bisphosphine 13. .... **S14**

6-(Dimethylphosphoryl)-1-chlorohexane (14) **S14**

1-(Dicyclohexylphosphoryl)-6-(dimethylphosphoryl)-hexane (15) **S15**

1-(Dicyclohexylphosphoryl)-6-(dimethylphosphoryl)- hexane (15) **S16**

3.4. Selective oxidation of phosphine substrates **S16**

4. NMR spectra of compounds **1–7**, **13** & **14** and phosphine oxidation reactions ... **S18**

5. EPR spectrum of complex **8** **S31**

6. XPS spectra of complexes **8-11**. **S32**

7. UV-vis spectra and their kinetic analysis **S33**

8. Crystallographic information **S49**

9. Ab-initio calculations and orbital analysis **S52**

10. HRMS spectra of compounds **1-11** **S55**

11. References. **S59**

1. General Information

Unless specified otherwise, all reagents were purchased from either Merck, Acros Organics, Alfa Aesar, or Strem Chemicals and used without further purification. All air-sensitive reactions were carried out under an atmosphere of purified nitrogen in a glovebox equipped with an inert gas purifier. Tetrahydrofuran (THF), acetonitrile (MeCN), diethyl ether (Et_2O), dichloromethane (DCM), toluene, N,N-dimethylformamide (DMF), and methanol (MeOH) were purified by passing through a column of activated alumina under an inert atmosphere. Anhydrous 1,2-dichloroethane (DCE) and benzene were purchased over molecular sieves from Acros Organics, and used without further purification. All chromatographic purifications were performed on a CombiFlash EZ-Prep instrument and SiO_2 columns.

NMR spectra were recorded on Bruker AVANCE 400 or Bruker Avance 500 spectrometers at 296 K. Residual solvent peaks were used as internal standards for ^{1}H and ^{13}C NMR spectra, respectively ($CDCl_3$: δ = 7.26/77.1 ppm, DCM-d_2: δ = 5.32/53.8 ppm, DMSO-d_6: δ = 2.50/40.0 ppm, CD_3OD: δ = 3.31, 4.85/47.7 ppm). NMR data are reported as follows: chemical shift, multiplicity (s = singlet, d = doublet, t = triplet, q = quartet, m = multiplet, br = broad), coupling constant(s), and integration.

Continuous-wave electron paramagnetic resonance (CW-EPR) spectra were recorded on a Bruker EMXplus instrument, operating at X-band (9.5 GHz), equipped with a high-sensitivity probehead. All measurements were performed at 5 K. Sample cooling was done using a Bruker cryostat, cooled with He gas, and employing a ColdEdge system. Frozen solutions were measured in standard 4mm EPR quartz tubes.

UV-vis absorption spectra, at various temperatures under N_2, were recorded on an Agilent Cary-5000 spectrophotometer. Temperature was controlled using a Unisoku CoolSpek cryostat. Kinetic measurements of reactions under N_2 were done in a 1cm quartz cuvette with a silicone septum screw cap attachment.

High-resolution mass spectrometry (HRMS) was performed on an HR QTOF−MS mass instrument using electrospray ionization (ESI+) or electroionization (EI) techniques. Non high resolution mass spectrometry was done using a MALDI TOF-MS (Bruker Autoflex II) instrument, with HCCA as matrix.

X-ray structures were solved by direct methods and refined on F^2 by a full-matrix least-squares procedure with SHELXL 2018/3 program [1] as implemented in Olex 2. [2] All the non-hydrogen atoms were refined anisotropically, and the hydrogen atoms were

refined as a riding model. Highly disordered atoms were squeezed by solvent mask option as implemented in the Olex 2.

X-ray photoelectron spectroscopy (XPS) spectra for complexes **8-11** were measured using a Kratos AXIS Supra spectrometer with Al Kα monochromatic radiation X-ray source (1486.6 eV) as the excitation source (Kratos, UK).

Electrochemical experiments were performed at room temperature under N_2 atmosphere using μAutolab type II potentiometer. The electrochemical cell used consisted of a low-volume three electrodes cell setup with glassy carbon working electrode (3 mm diameter), platinum counter/auxiliary electrode and Ag/$AgNO_3$ (0.01M in 0.1 M tetra-butyl ammonium hexafluorophosphate in acetonitrile) reference electrode (Pine research instrumentation). 0.1 M tetrabutylammonium hexafluorophosphate has been used as a supporting electrolyte.

2. Computational details

Calculations regarding the theoretical double-imination reaction between model macrocycle $\mathbf{5^{*}_{2NH2}}$ and the three linker candidate molecules dibenzofuran-4,6-dicarbaldehyde (DBF-CHO), xanthene-4,5-dicarbaldehyde (XANT-CHO), and pyridine-2,6-dicarbaldehyde (PYR-CHO) were performed using the ORCA 5 program package[3], with the B3LYP functional and a double zeta basis set (def2-SVP) with an auxiliary def2/J basis set for the addition of an RIJCOSX approximation to speed up SCF convergence. In all cases, the Becke-Johnson damping dispersion correction[4] was added in order to increase the accuracy of the SPE values calculated. The final ΔE values, obtained for the comparison between the three linker candidates, were calculated according to **Scheme S1**:

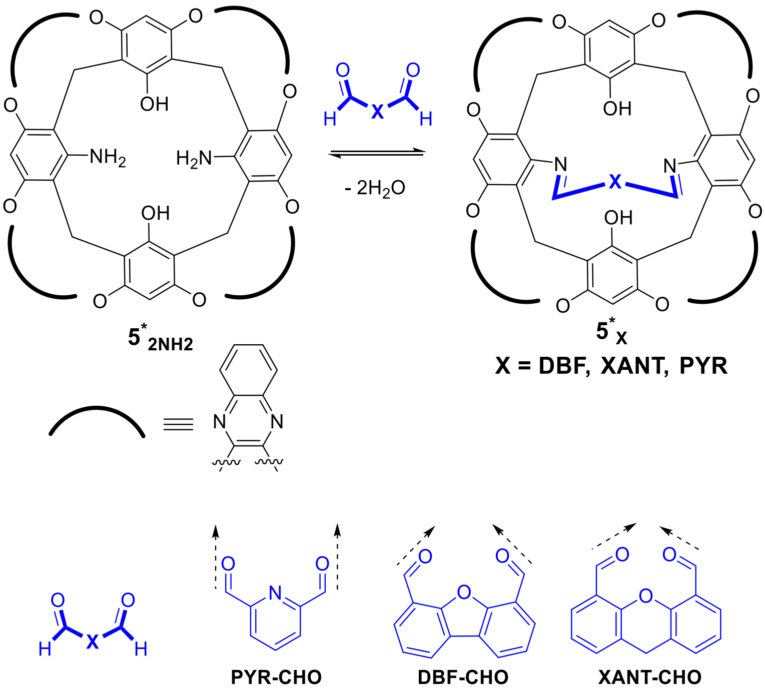

	PYR-CHO	DBF-CHO	XANT-CHO
bite angle:	~0°	~90°	~120°
**Δ*E*** (kcal/mol):	31.2	16.0	31.6

$$\Delta E = \text{SPE}(\mathbf{5}^{*}_{\mathbf{X}}) + 2\text{SPE}(H_2O) - \text{SPE}(\mathbf{X\text{-}CHO}) - \text{SPE}(\mathbf{5}^{*}_{\mathbf{2NH2}})$$

Scheme S1. The method used for the SPE calculations on the theoretical condensation reactions between $\mathbf{5}^{*}_{\mathbf{2NH2}}$ and the linkers **DBF-CHO**, **XANT-CHO**, and **PYR-CHO**.

The single point energy (SPE) calculations on model complexes **8*** and **9*** were performed using the Gaussian 16 suite of programs[5] employing the unrestricted density functional theory (UDFT). Hybrid functional B3LYP was used in all said calculations.[6-7] To reduce the computational cost, model compounds **8*** and **9*** were simplified by replacing the n-butyl groups with methyl groups, and the quinoxaline units with hydrogen atoms, in the solved crystal structures of **8** and **9**. No optimization was performed on these modified structures. All single point calculations on the modified crystal structures were performed using a triple zeta basis set (def2-TZVP)[8-9] for Mn, N, and O atoms and a double zeta basis set (6-31G*) for C and H.[10] Time-dependent density functional (TD-DFT) calculations were performed using the same functional and basis sets previously mentioned. Solvent effects were considered by employing the Conductor-Like Polarizable Continuum Model (CPCM) for DCM.[11-13]

Multi-reference ab-initio calculations were performed employing the CASSCF/NEVPT2 methodology, as implemented in the ORCA 5 program package. The starting orbitals were generated from the restricted Kohn-Sham (RKS) DFT

calculations with a BP86 functional.[14-16] To take into account the scalar relativistic effect, the DKH (Douglas-Kroll-Hess) Hamiltonian with the triple zeta DKH-def2-TZVP basis set for Mn, O, and N atoms, and the double zeta DKH-def2-SVP basis set for C and H were used.[17] For the CASSCF calculations on complex **9***, initial calculations were performed considering 3 unpaired electrons occupying the 3*d* orbitals of the Mn center (CAS (3,5)) and subsequently with a larger active space containing 11 electrons in the Mn-3*d* based molecular orbitals (MOs), the three oxo *p* orbitals and the bonding d_{x2-y2} orbital between the Mn center and the four equatorial ligands (CAS(11, 9)). The CASSCF calculations were performed on 10 quartet and 9 doublet states. To include the dynamic correlation, N-electron valence perturbation theory (NEVPT2) calculations were performed on top of the SA-CASSCF wave functions.[18] Spin-Hamiltonian properties were computed with the universal effective Hamiltonian (EHA) approach.[19] Compositions of molecular orbitals were determined employing the Multiwfn software.[20-21] NBO analysis was performed using NBO 7 program.[22]

All EPR simulations of the CW-EPR spectra were performed using EasySpin software.[23]

3. Synthetic procedures

2,8-Dibutyldibenzofuran-4,6-dicarbaldehyde, 2-(tertbutylsulfonyl)iodosylbenzene (sPhIO), and Dicyclohexyl(3-(dimethylphosphoryl)hexyl)phosphine oxide were synthesized following literature procedures.[24-26]

3.1. Preparation of the cavitand

Triol[4]arene (1):

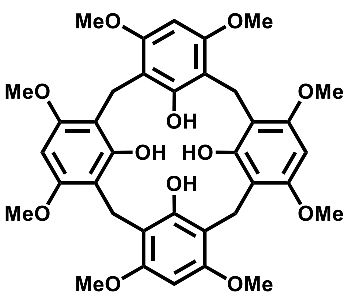

In a 250 mL round bottom flask, equipped with a Teflon coated stirring bar, 3,5-dimethoxyphenol (1.54 g, 10.0 mmol) and paraformaldehyde (300 mg, 10.0 mmol) were suspended in 80 mL of 1,2-DCE. To this suspension 5 mL of trifluoroacetic acid (TFA) were added. Immediate dissolution of the solids and rapid color change to dark red were observed. The solution was stirred at rt for 40 hrs. The crude mixture was

evaporated to dryness, and the product purified by flash chromatography (silica gel, DCM). Collection and evaporation of the first eluted fraction yielded 0.75 g (45% yield) of pure **1** as a white amorphous solid. If necessary, colorless crystals of **1** can be obtained by recrystallization from hot ethyl acetate (not needed for further derivatization).

A single crystal of **2** suitable for XRD was grown of MeCN into its solution in $CHCl_3$.

^{1}H NMR (400 MHz, $CDCl_3$): δ = 9.92 (s, 4H, O*H*), 6.02 (s, 4H, Ar-*H*), 3.86 (s, 8H, Ar_2-C*H*$_2$), 3.77 (s, 24H, OC*H*$_3$) pm.
^{13}C NMR (101 MHz, $CDCl_3$): δ = 157.13, 152.42, 108.70, 89.56, 55.79 (O*C*H_3), 16.51 (Ar_2-*C*H_2) ppm.
HR-MS (ESI+): m/z calculated for $C_{36}H_{41}O_{12}$ $[M+H]^+$ = 665.2598, found = 665.2607.

Bis(trifluoromethanesulfonate)octamethoxytriol[4]arene (2):

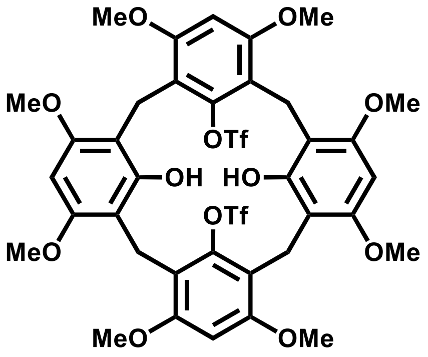

In a 250 mL round bottom flask, equipped with Teflon-coated magnetic stir bar, **1** (2 g, 3.0 mmol) and 1,8-bis(dimethylamino)naphthalene (3.15 g, 14.7 mmol) were dissolved in 80 mL DCM. The solution was cooled to 0°C in an ice bath, and triflic anhydride (2 mL, 11.9 mmol) was added dropwise. Immediate color change to orange pink was observed. The solution was allowed to warm to rt and stirred for 3 hrs, during which the color of the solution became gradually darker. The solution was diluted with 100 mL DCM, washed three times with 10% aqueous HCl, and once with brine. The organic phase was dried over Na_2SO_4, filtered, and evaporated to dryness. The orange residue was washed with a minimal amount of MeOH, yielding 1.84 g (66% yield) of **2** as a white crystalline solid.

A single crystal of **2** suitable for XRD was grown by a vapor diffusion of ether into its solution in DCM.

^{1}H NMR (400 MHz, $CDCl_3$): δ = 6.15 (s, 2H, Ar-*H*), 6.06 (s, 2H, Ar-*H*), 4.36 (s, 2H, OH), 4.20 (d, *J* = 15.0 Hz, 4H, Ar_2-C*H*$_2$), 3.89 (s, 12H, OC*H*$_3$), 3.56 (d, *J* = 15.0 Hz, 4H, Ar_2-C*H*$_2$), 3.30 (s, 12H, OC*H*$_3$) ppm.

^{13}C NMR (101 MHz, $CDCl_3$): δ = 157.63, 156.82, 153.75, 146.19, 116.30, 108.89, 97.82, 88.18, 56.14 (OCH_3), 18.87 (Ar_2-CH_2) ppm.

HR-MS (ESI+): m/z calculated for [$C_{38}H_{37}F_6O_{16}S_2$] $[M+H]^+$ = 929.1584, found = 929.1609.

(Dibenzylamino)octamethoxytriol[4]arene (3):

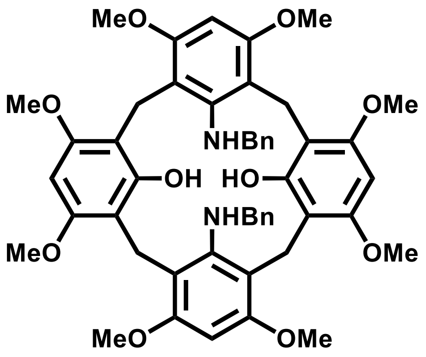

Inside a glovebox, an oven dried Schlenk flask, equipped with a Teflon-coated magnetic stir bar, was charged with **2** (0.75 g, 0.8 mmol), K_3PO_4 (0.7 g, 3.5 mmol), CuI (17 mg, 0.1 mmol), anhydrous ethylene glycol (680 µL, 12.1 mmol), anhydrous benzylamine (250µL, 2.3 mmol), and anhydrous toluene (20mL). The flask was then sealed and heated in an oil bath to 130 °C for 24 hrs. After cooling to rt, the solution was diluted with 100 mL of DCM, washed once with a concentrated aqueous solution of NH_4Cl, three times with 10% aqueous HCl, once with saturated aqueous $NaHCO_3$, and finally once with brine. The organic phase was dried over anhydrous Na_2SO_4, filtered, and evaporated to dryness. The crude product was triturated with a minimal amount of THF, yielding 395mg (58% yield) of pure **3**, as a white crystalline solid. This compound exists as a mixture of two atropisomers, which can be separated by chromatography (silica gel, 5% ethyl acetate / DCM). However, this separation is not necessary for the next step.

XRD quality crystals of **3** (both atropisomers) were grown by a vapor diffusion of ether into their solutions in $CHCl_3$.

Atropisomer A (*cone*) ^{1}H NMR (400 MHz, $CDCl_3$): δ = 9.53 (br s, 4H, NH, OH), 7.34-7.36 (m, 4H, Bn(Ar-*H*)), 7.19-7.24 (m, 6H, Bn-*H*), 6.11 (s, 2H, Ar-*H*), 6.00 (s, 2H, Ar-*H*), 4.28 (d, J=13.82 Hz, 4H, Ar_2-C*H*$_2$), 4.03 (s, 4H, Bn(C*H*$_2$)), 3.86 (s, 12H, OC*H*$_3$), 3.63 (s, 12H, OC*H*$_3$), 3.23 (d, J=13.82 Hz, 4H Ar_2-C*H*$_2$) ppm.

^{13}C NMR (101 MHz, $CDCl_3$): δ = 157.54, 157.17, 156.45, 144.62, 138.70, 128.70, 127.72, 127.24, 116.76, 107.83, 94.00,87.23, 56.74, 56.02, 55.70, 18.66 (Ar_2-*C*H_2) ppm.

Atropisomer B (*1, 3-alternate*) ^{1}H NMR (400 MHz, $CDCl_3$): δ = 9.54 (s, 2H, O*H*), 7.14 (dd, *J* = 5.0, 1.8 Hz, 6H, Bn(Ar-*H*)), 6.93 – 6.84 (m, 4H, Bn(C*H*$_2$)), 6.19 (s, 2H, Ar-*H*), 5.90 (s, 2H, Ar-*H*), 4.17 (d, *J* = 15.7 Hz, 4H, Ar$_2$-C*H*$_2$), 3.73 (s, 9H, OC*H*$_3$), 3.71 (s, 9H, OC*H*$_3$), 3.63 (s, 4H, NH), 3.42 (d, *J* = 15.7 Hz, 4H, Ar$_2$-C*H*$_2$) ppm.

^{13}C NMR (101 MHz, $CDCl_3$): δ = 157.39, 156.72, 156.49, 146.24, 139.31, 128.15, 128.12, 126.87, 115.94, 107.70, 93.33, 87.47, 56.26, 55.80, 54.75, 19.69 (Ar$_2$-*C*H$_2$) ppm.

HR-MS (ESI+): m/z calculated $C_{50}H_{54}N_2O_{10}$ $[M+H]^+$ = 843.3857, found = 843.3861.

(Diamino)octamethoxytriol[4]arene (4):

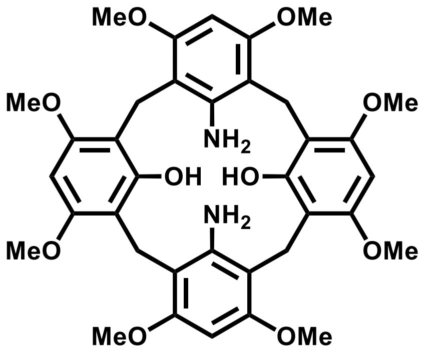

In a 250mL hydrogenation flask, **3** (700mg, 0.8 mmol) and Pearlman's Catalyst (20% $Pd(OH)_2$/C, 250mg) were suspended in 90 mL of THF. The reaction flask was filled with H_2 gas (65 psi) and heated to 70 °C for 18 hrs. The reaction mixture was passed through a short pad of celite, and the product was extracted from the catalyst using copious amounts of DCM. The combined organic extracts were collected and evaporated to dryness under reduced pressure. The solid residue was triturated with a minimal amount of ethyl acetate or THF, yielding 517 mg (94% yield) of pure **4**, as an amorphous white solid. Colorless crystals of **4** can be obtained by recrystallization from hot ethyl acetate (not necessary for the next step).

^{1}H NMR (400 MHz, DMSO-d$_6$): δ = 7.44 (br s, 4H, N*H*$_2$), 6.04 (s, 2H, Ar-*H*), 5.94 (s, 2H, Ar-*H*), 3.80 (s, 2H, O*H*), 3.76 (s, 12H, OC*H*$_3$), 3.66 (s, 8H, Ar$_2$-C*H*$_2$), 3.57 (s, 12H, OC*H*$_3$) ppm.

^{13}C NMR (101 MHz, DMSO-d$_6$, 373K): δ = 155.81, 155.65 143.47, 108.21, 107.97, 89.11, 87.62 55.51, 55.22, 16.54 (Ar$_2$-*C*H$_2$) ppm.

HR-MS (ESI+): m/z calculated $C_{36}H_{43}N_2O_{10}$ $[M+H]^+$ = 663.2918, found = 663.2922.

Bicyclic octamethoxytriol[4]arene 5:

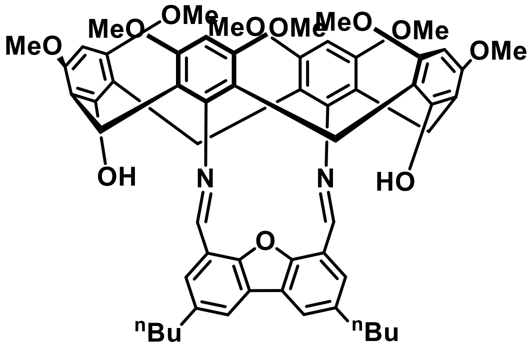

In a 100 mL round bottom flask, equipped with a $CaCl_2$ drying tube, **4** (300 mg, 0.45 mmol) and 2,8-dibutyldibenzofuran-4,6-dicarbaldehyde (167.5 mg, 0.50 mmol) were suspended in anhydrous benzene (30 mL). 10 drops of TFA were added and the mixture was stirred at 80°C for 4 hrs. The solvent was removed under vacuum, the residue was dissolved in 50mL DCM and washed with saturated $NaHCO_3$, followed by brine. The organic phase was dried with anhydrous Na_2SO_4, filtered, and evaporated to dryness. The crude product was washed with diethyl ether, yielding 414 mg (95% yield) of **5**, as a bright yellow amorphous solid.

^{1}H NMR (400 MHz, $CDCl_3$): δ = 8.78 (s, 2H, N=C*H*), 8.17 (d, J = 1.6 Hz, 2H, Ar-*H*), 7.88 (d, J = 1.6 Hz, 2H, Ar-*H*), 6.43 (s, 2H, Ar-*H*), 5.46 – 5.13 (br s, 2H, O*H*), 4.15 (d, *J* = 15.9 Hz, 4H, Ar_2-CH_2), 3.82 (s, 12H, OCH_3), 3.43 (br s, 4H, Ar_2-CH_2), 3.16 (br s, 12H, OCH_3), 2.91 (t, *J* = 7.7 Hz, 4H, nBu(CH_2)), 1.81 – 1.69 (m, 4H, nBu(CH_2)), 1.45 (m, 4H, nBu(CH_2)), 0.98 (t, *J* = 7.3 Hz, 6H, nBu(CH_3)) ppm.

^{13}C NMR (101 MHz, $CDCl_3$): δ = 163.16, 156.94, 155.89, 154.86, 152.15, 142.22, 140.36, 134.35, 126.93, 124.69, 116.78, 106.93, 95.85, 56.20, 54.79, 35.09 (Ar-**C**$H_2CH_2CH_2CH_3$), 34.04 (Ar-CH_2**C**$H_2CH_2CH_3$), 22.28 (Ar-CH_2CH_2**C**H_2CH_3), 17.65 (Ar_2-*C*H_2**)**, 13.89 (Ar-$CH_2CH_2CH_2$**C**H_3) ppm.

HR-MS (ESI+): m/z calculated for $C_{58}H_{62}N_2O_{11}$ $[M+H]^+$ = 963.4432, found = 963.4451.

A single crystal of **5** suitable for XRD was grown by vapor of ether into its $CHCl_3$ solution.

Bicyclic decahydroxytriol[4]arene 6:

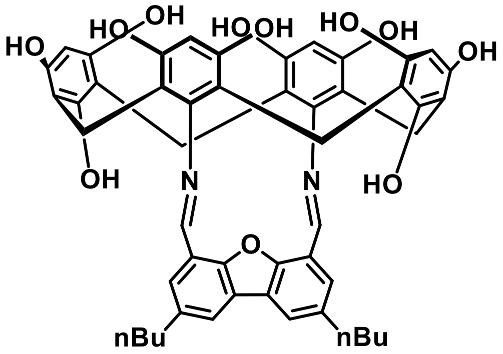

A 100 mL Schlenk tube was charged with **5** (300 mg, 0.311 mmol), BBr_3 (4.68g, 18.68 mmol) and 20 mL anhydrous 1,2-DCE. The resulting reaction mixture was stirred at 100°C for 1.5 days under an atmosphere of nitrogen. The excess BBr_3 was quenched with anhydrous MeOH, and the brown solution was evaporated to dryness under reduced pressure. The resulting yellowish-brown solid was purified by flash chromatography (Silica gel, 10% MeOH / DCM) – yielding 190mg (71% yield) of pure **6** as a light yellow solid.

^{1}H NMR (400 MHz, DMSO-D_6**):** δ = 11.09 (s, 2H, O*H*), 9.70 (s, 4H, O*H*), 9.05 (s, 4H, O*H*), 8.48 (s, 2H, N=C*H*), 8.31 (d, J=1.63 Hz, 2H, Ar-*H*), 7.85 (d, J=1.63 Hz, 2H, Ar-*H*), 6.04 (s, 2H, Ar-*H*), 5.87 (s, 2H, Ar-*H*), 3.54 (d, J=23.6 Hz, 4H, Ar_2-CH_2), 3.50 (d, J=23.6 Hz, 4H, Ar_2-CH_2), 2.85 (t, J=7.77 Hz, 4H, nBu(CH_2)), 1.73 (m, 4H, nBu(CH_2)), 1.40 (m, 4H, nBu(CH_2)), 0.95 (t, J=7.53 Hz, 6H, nBu(CH_3)) ppm.

^{13}C NMR (101 MHz, DMSO-D_6**):** δ = 159.41, 156.81, 153.63, 151.77, 151.55, 149.35, 137.77, 132.58 (C_{Ar}), 124.44, 124.02 (C_{Ar}), 119.97, 109.26, 107.15, 100.15 (C_{Ar}), 93.99 (C_{Ar}), 34.51 (Ar-**C**$H_2CH_2CH_2CH_3$), 33.43 (Ar-CH_2**C**$H_2CH_2CH_3$), 21.77 (Ar-CH_2CH_2**C**H_2CH_3), 18.59 **(**Ar_2-*C*H_2**)**, 13.86 (Ar-$CH_2CH_2CH_2$**C**H_3) ppm.

HR-MS (ESI+): m/z calculated for $C_{58}H_{62}N_2O_{11}$ $[M+H]^+$ = 851.3174; found = 851.3148.

Cavitand 7:

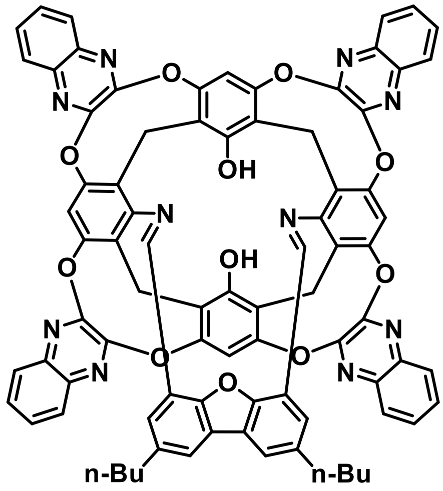

In an oven dried Schlenk tube, equipped with a Teflon-coated magnetic stir bar, **6** (250 mg, 0.293 mmol) and 2,3-dichloroquinoxaline (245.55 mg, 1.23 mol) were dissolved in anhydrous DMF. To this solution was added K_2CO_3 (405.03 mg, 2.93 mmol). The Schlenk flask was sealed, transferred to an oil-bath pre-heated to 70°C, and the reaction mixture was stirred at 70°C for 12 hrs. The reaction mixture was allowed to cool to room temperature, diluted with an equal volume of DCM, and filtered to remove most of the undesired solids. The solvent was removed under reduced pressure, while maintaining the temperature of the water bath below 60°C. The solid residue was suspended in 5% aqueous HCl, stirred for 30 min, and extracted several times with DCM. The combined organic layers were washed with brine, dried with anhydrous Na_2SO_4, and filtered. The solvent was removed under vacuum, and the crude product was purified by flash chromatography (silica gel, 0-1% MeOH / DCM) to obtain 180 mg (45% yield) of **7** as an amorphous light beige to white solid.

A single crystal of **7**, suitable for XRD, was grown by a slow vapor diffusion of diethyl ether into its concentrated DCM solution at rt.

^{1}H NMR (400 MHz, $CDCl_3$): δ = 11.06 (s, 2H, O*H*), 8.40 (s, 2H, N=C*H*), 8.03 (d, *J*=1.77 Hz, H, Ar-*H*), 7.91-7.89 (m, 4H, Ar-*H*), 7.78-7.75 (m, 4H, Ar-*H*), 7.56 (d, J=1.77 Hz, Ar-*H*), 7.55-7.49 (m, 8H, Ar-*H*), 7.46 (s, 2H, Ar-*H*), 4.6 (d, J= 13.90 Hz, 4H, Ar_2-CH_2), 3.7 (d, J= 13.90 Hz, 4H, Ar_2-CH_2), 2.87 (t, *J*= 7.85 Hz, 4H, nBu(CH_2)), 1.75 (m, 4H, nBu(CH_2)), 1.47 (m, 4H, nBu(CH_2)), 0.98 (t, 7.28 Hz, 6H, nBu(CH_3)) ppm.

^{13}C NMR (101 MHz, $CDCl_3$): δ = 161.09, 157.25, 153.73, 153.40, 152.96, 152.88, 151.76, 149.79, 139.66, 138.50, 132.43 (CH, Ar), 129.19, 128.93 (CH, Ar), 128.39 (CH, Ar), 127.55 (CH, Ar), 125.48, 124.48 (CH, Ar), 122.49, 119.80, 117.79, 114.70 (CH, Ar), 108.84 (CH, Ar), 35.55 (**C**$H_2CH_2CH_2CH_3$), 34.23 (CH_2**C**$H_2CH_2CH_3$), 22.26 (CH_2CH_2**C**H_2CH_3), 20.98 (Ar_2-CH_2), 14.14 ($CH_2CH_2CH_2$**C**H_3) ppm.

HR-MS (ESI+): m/z calculated for $C_{82}H_{55}N_{10}O_{11}$ $[M+H]^+$ = 1355.4052, found = 1355.4011

3.2. Preparation of the metallocavitand complexes

Mn(II) metalocavitand (8)

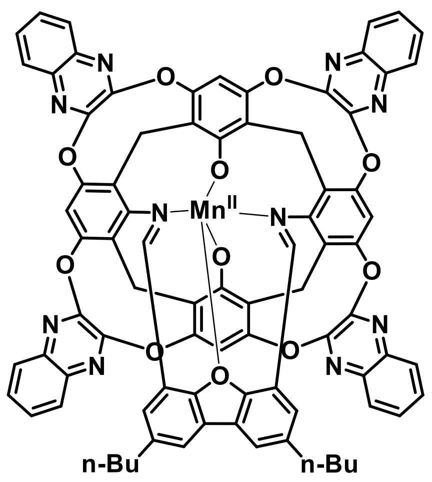

In a nitrogen glovebox, **7** (100 mg, 0.073 mmol) was suspended in 15 mL of anhydrous THF and treated with lithium bis(trimethylsilyl)amide (LiHMDS, 31 mg, 0.183 mmol). Immediate color change from white to vivid orange was observed, and gradually all the solid dissolved. Upon full dissolution of **7**, Mn(II)(OTf)$_2$(MeCN)$_2$ (75mg, 0.185 mmol) was added. The resulting dark brown solution was stirred at rt for 3 hrs and centrifuged to collect the purple-grey precipitate. This precipitate was washed three times with 10 mL anhydrous THF, centrifuged, and the solute impurities discarded. The resulting crude product was dissolved in anhydrous DCM, passed through a short pad of celite to remove excess manganese precursor and byproducts, and evaporated to dryness, affording 55 mg (53% yield) of **8** as a lavender-purple powder. Extremely pure samples of **8** may be obtained, albeit at greatly reduced yields, by recrystallization from DCM / THF (liquid diffusion). If the work-up or recrystallization are performed under air, or using non-anhydrous solvents, the water adduct is obtained instead (**8•H$_2$O**). Conversely, the recrystallization may be performed from DCM / MeCN (liquid diffusion). In this case, the MeCN adduct **8•MeCN** is formed.

UV-Vis: $\lambda_{max/nm}$ (DCM, ε, $M^{-1}cm^{-1}$) = 495 (748).

EA: calculated for **8**•THF•DCM**:** C, 66.76; H, 3.99; N, 8.95; found: C, 66.76, H, 3.97; N, 8.60.

HR-MS (ESI+): m/z calculated for $C_{82}H_{53}MnN_{10}O_{11}$ $[M+H]^+$ = 1408.3276, found 1408.3250.

Evans method (DCM-*d*$_2$ under N$_2$): μ_{eff} (Experimental) = 5.58 BM (Theoritical value 5.91 BM).

A single crystal, suitable for XRD, was grown by slow vapor diffusion of diethyl ether to a DCM solution at rt.

Mn(III)-OH metallocavitand (10)

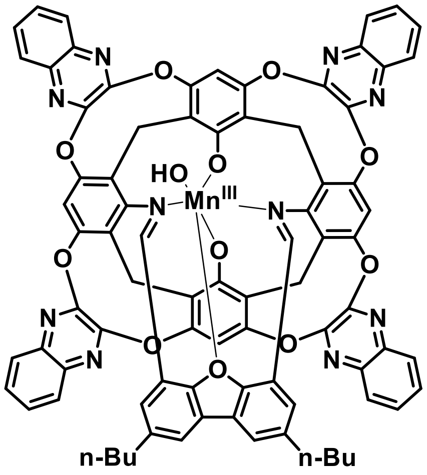

8 (20 mg, 0.014 mmol) was dissolved in 10 mL of 1,2-difluorobenzene (DFB). 50 molar equivalents of ^{t}BuOOH (5.5M solution in nonane) were added. Immediate color change from light pink to indigo was observed. The solvent was evaporated under reduced pressure to dryness, and the resulting dark purple residue washed with Et$_2$O and centrifuged three times. The collected precipitate was dried completely under high vacuum for 12 hrs, yielding 15 mg (74% yield) of **10** of satisfactory purity.

A single crystal, suitable for XRD, was grown by a slow evaporation of its DCM solution at rt.

UV-Vis: $\lambda_{max/nm}$ (DCM, ε, $M^{-1}cm^{-1}$) = 478 (1,519), 504 (1,719), 609 (1,011).

HR-MS (ESI+): m/z calculated for $C_{82}H_{52}MnN_{10}O_{11}$ $[M-H_2O]^+$ = 1407.3197; found = 1407.3185.

Evans method (DCM-*d*$_2$ under N$_2$): μ_{eff} = 4.74 BM (Theoritical value 4.89 BM).

Mn(III)-Cl metallocavitand (11)

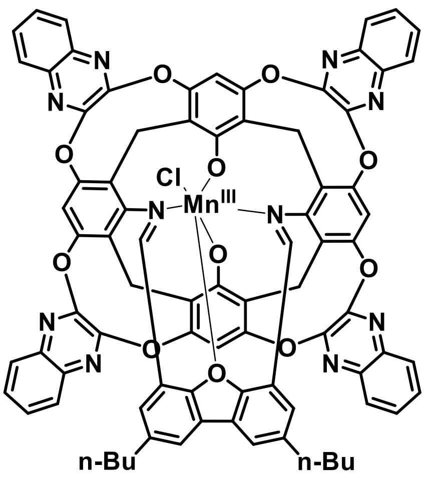

8 (20 mg, 0.014 mmol) was dissolved in 10 mL of DCM. 50 molar equivalents of ^{t}BuOOH (5.5M solution in nonane) were added. Gradual color change from light pink to dark emerald green was observed over the course of 30 minutes. The solvent was evaporated under reduced pressure to dryness, and the resulting dark green residue was washed with 10 mL Et_2O and centrifuged three times. Traces of Et_2O were removed from the green precipitate by evaporation under high vacuum for 12 hrs, yielding 18 mg (88% yield) of **11** of satisfactory purity.

UV-vis: $\lambda_{max/nm}$ (DCM, ε, $M^{-1}cm^{-1}$) = 620 (1,626), 714 (2,169).

HR-MS (ESI+): m/z calculated for M^+ = 1442.2886; found = 1442.2886.

Evans method (DCM-*d*$_2$ under N_2): μ_{eff} = 4.61 BM (Theoritical value 4.89 BM).

***In-situ* generation of the Mn(IV)=O intermediate (9)**

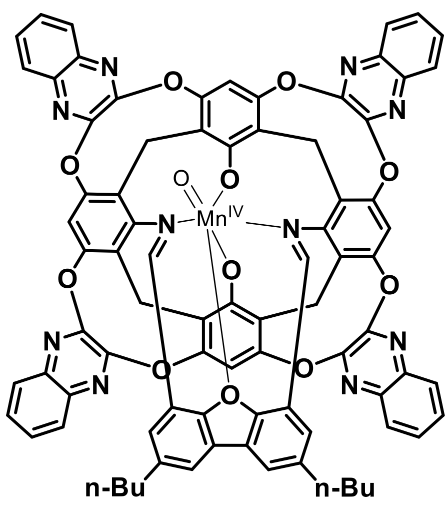

4 mg of **8** were dissolved in 3 mL of an anhydrous chlorinated solvent (e.g., DCM, 1,2-DCE, or $CHCl_3$, as necessary) under N_2. 1.0 mg (1.05 molar equivalents) of sPhIO was dissolved in a minimal amount of the same solvent and added to the solution of the complex. Immediate color change from light pink to blood red was observed. Complete oxidation of **8** to **9** was observed, by UV-vis absorption spectroscopy, to take place within 2 minutes at r.t under these conditions.

This intermediate is short lived and cannot be isolated by conventional means. However, a single crystal suitable for XRD was grown via slow vapor diffusion of $CFCl_3$ to its DCM solution under N_2 at -30°C.

UV-Vis: $\lambda_{max/nm}$ (DCM, ε, $M^{-1}cm^{-1}$) = 507 (2,624).

HR-MS (ESI+): m/z calculated for: $C_{82}H_{52}MnN_{10}O_{12}$ $[M]^+$ = 1423.3147, found: 1423.3147;

Evans method (DCM-*d*2 under N2): μ_{eff} = 3.88 BM (Theoretical value 3.88 BM)

3.3. Preparation of an asymmetric bisphosphine 13.

Synthesis of **13** was done following a literary procedure,[26] starting from 1-bromo-6-chlorohexane, according to Scheme S2.

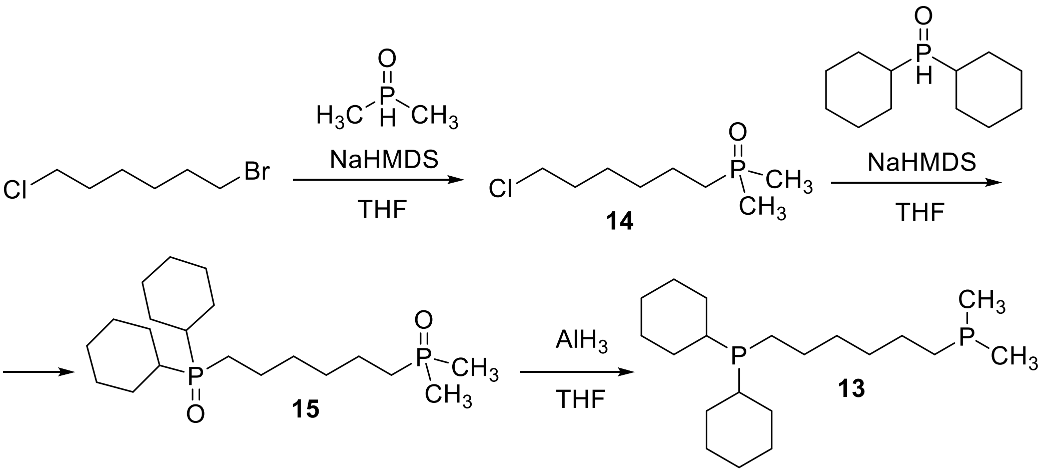

Scheme S2. The synthetic route employed for the preparation of bisphosphine **13**.

6-(Dimethylphosphoryl)-1-chlorohexane (14)

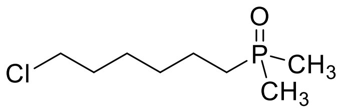

In an inert glovebox environment, a Schlenck tube was loaded with dimethylphosphine oxide (1.0g, 12.8 mmol) and anhydrous THF (15 mL). To this solution, NaHMDS (2.58g, 1.1 eq) was added dropwise at r.t. After stirring for a further 15 min, this phosphinite suspension was slowly added to a solution of 1-bromo-6-chlorohexane (2.55g, 1 eq) in THF (15 mL). The mixture was stirred at r.t for 6 hours, and subsequently quenched with an equal volume of deionized water and extracted with 5 subsequent portions of DCM (20 mL). The resulting crude product was washed multiple times with hexane – affording 14 as a viscous clear oil (1.76g, 70% yield).

^{1}H NMR (400 MHz, C_6D_6): δ = 3.14 (t, J = 6.70 Hz, 2H), 1.45-1.36 (m, 2H), 1.36-1.24 (m, 2H), 1.22-0.99 (m, 6H), 0.96 (d, J = 12.87 Hz, 6H) ppm.

^{13}C NMR (101 MHz, C_6D_6): δ = 45.0, 32.6, 31.9 (d, J = 69.0 Hz), 30.3 (d, J = 14.1 Hz), 26.6, 22.1 (d, J = 3.6 Hz), 16.4 (d, J = 69.0 Hz) ppm.

^{31}P NMR (165 MHz, C_6D_6): δ = 35.6 ppm.

1-(Dicyclohexylphosphoryl)-6-(dimethylphosphoryl)-hexane (15)

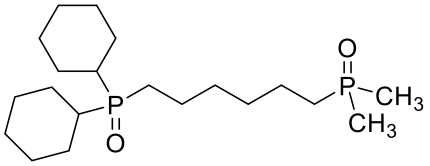

Dicyclohexylphosphine oxide (500mg, 2.33 mmol) was suspended in anhydrous THF (20 mL) in an inert glovebox environment. NaHMDS (476 mg, 1.1 eq) was added slowly to the solution, and the resulting suspension was stirred at r.t for 15min. This suspension was added dropwise at r.t to a solution of **14** (459 mg, 1 eq) in anhydrous THF (10 mL) at r.t, and the mixture was stirred for an additional 6 hours, and subsequently quenched with deionized water (reaction progress may be monitored by ^{31}P NMR of the crude mixture). The product was extracted using copious amounts of DCM, evaporated to dryness, and the resulting yellow oil was purified by liquid chromatography (0-20% MeOH in DCM) to yield the product as a white solid (891 mg, 55% yield).

^{1}H NMR (400 MHz, $CDCl_3$): δ = 1.99 – 1.56 (m, 20H), 1.41 (d, 6H, J=12.63 Hz), 1.41-1.10 (m, 14H) ppm.

^{13}C NMR (101 MHz, $CDCl_3$): δ = 36.34 (CH, Cy, d, J=63.65 Hz), 31.81 (CH_2, Hex, d, J=69.16 Hz), 31.24 (CH_2, Hex, d, J=12.44 Hz), 30.67 (CH_2, Hex, d, J=14.31 Hz), 26.66 (CH_2, Cy, dd, J=2.93 Hz, 12.17 Hz), 26.02 (CH_2, Cy, s), 25.84 (CH_2, Cy, dd, J =2.84 Hz, 32.71 Hz), 23.73 (CH_2, Hex, d, J= 61.29 Hz), 21.92(CH_2, Hex, d, J=4.30 Hz), 21.86 (CH_2, Hex, d. J=3.75 Hz), 16.22 (CH_3, d, J=68.52 Hz) ppm.

^{31}P NMR (165 MHz, $CDCl_3$): δ = 50.7, 42.5 ppm.

1-(Dicyclohexylphosphoryl)-6-(dimethylphosphoryl)- hexane (15)

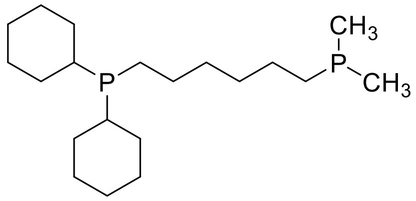

A freshly prepared THF solution of alane (16.4 mmol, 16.4 mL, 6.4 equivalents) was added to a THF solution of the bis-phosphine oxide derivative **15** in a Schlenk tube under N_2. The mixture was heated to 66°C overnight. After completion of the reaction (monitored by ^{31}P NMR of the crude mixture), the solution was cooled to r.t and quenched by slow addition of anhydrous MeOH inside the glove box. The solvent was removed under vacuum and the desired product was obtained by eluting the crude residue through a short column of anhydrous silica (~6 cm x 1.5 cm) with a 10% MeOH/DCM mixture. Removal of the solvent yielded a colorless oil as the desired product **13** (934 mg, 58% yield).

^{1}H NMR (400 MHz, C_6D_6): δ = 1.89 – 1.86 (m, 2H), 1.79-1.73 (m, 6H), 1.59-1.50 (m, 6H), 1.46-1.36 (m ,10H), 1.31-1.18 (m, 10H), 0.87 (d, J=2.22Hz, 6H) ppm.

^{13}C NMR (101 MHz, C_6D_6): δ = 34.05 (d, J=15.2 Hz), 32.79 (d, J=10.80 Hz), 31.72 (d, J=11.86 Hz), 31.54 (d, J=10.97 Hz), 30.88 (d, J=15.28Hz), 29.50 (d, J=8.66 Hz), 29.07 (d, J =20.22), 27.80 (d, J= 8.80 Hz), 27.71 (d, J=5.16 Hz), 20.01 (s), 26.25 (d, J=10.06 Hz), 22.05 (d, J=18.72 Hz), 14.36 (d, 14.95 Hz) ppm.

^{31}P NMR (165 MHz, C_6D_6): δ = -5.88, -53.27 ppm.

3.4.Selective oxidation of phosphine substrates

All phosphine oxidation experiments were performed in J. Young-tubes, sealed inside a nitrogen glovebox, and monitored using ^{31}P NMR. Quantitative measurements were done using an inverse-gated H-decoupled pulse program (zgig), with a sufficiently long D1 interval of 15 sec, as determined by appropriate inversion-recovery experiments. In general, the relaxation times of phosphines were found to be in the range of 0.5-2 sec, and shortened by 1-2 orders of magnitude during interaction with Mn complexes in solution. In all cases, complete release of phosphines and phosphine-oxides, from the Mn species in solution, was achieved by the addition of a large excess of 4-dimethylaminopyridine (DMAP) and heating the mixture inside the sealed J. Young-tube to 80°C for 30 min prior to the measurement. This served to both decompose Mn-phosphine or Mn-phosphine oxide adducts formed, as well as to quench any residual Mn(IV)-oxo species. Two control experiments were performed for the oxidation of

bisphosphine **13** – with 1 molar equivalent of sPhIO or with excess MnO_2. Both reactions were performed inside sealed J. Young tubes, under N_2, using 1,2-DCE as solvent.

4. NMR spectra of compounds 1–7, 13 & 14

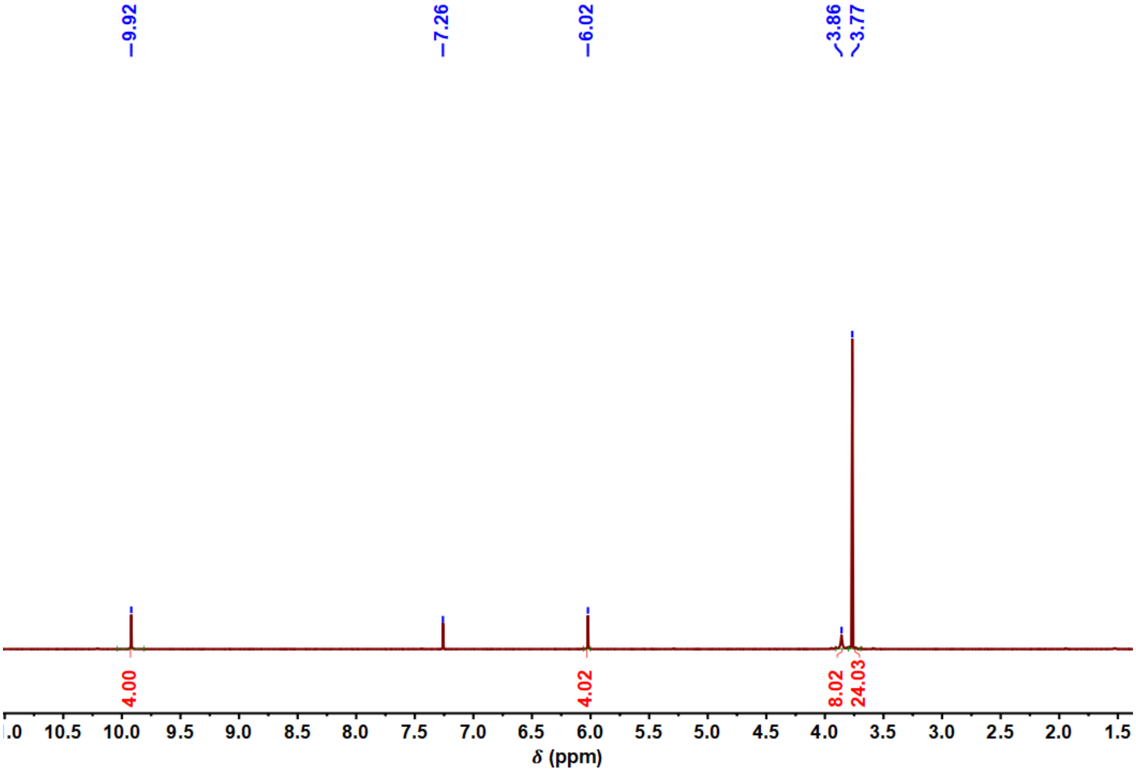

Figure S1. ^{1}H-NMR spectrum of **1** in $CDCl_3$ recorded at room temperature.

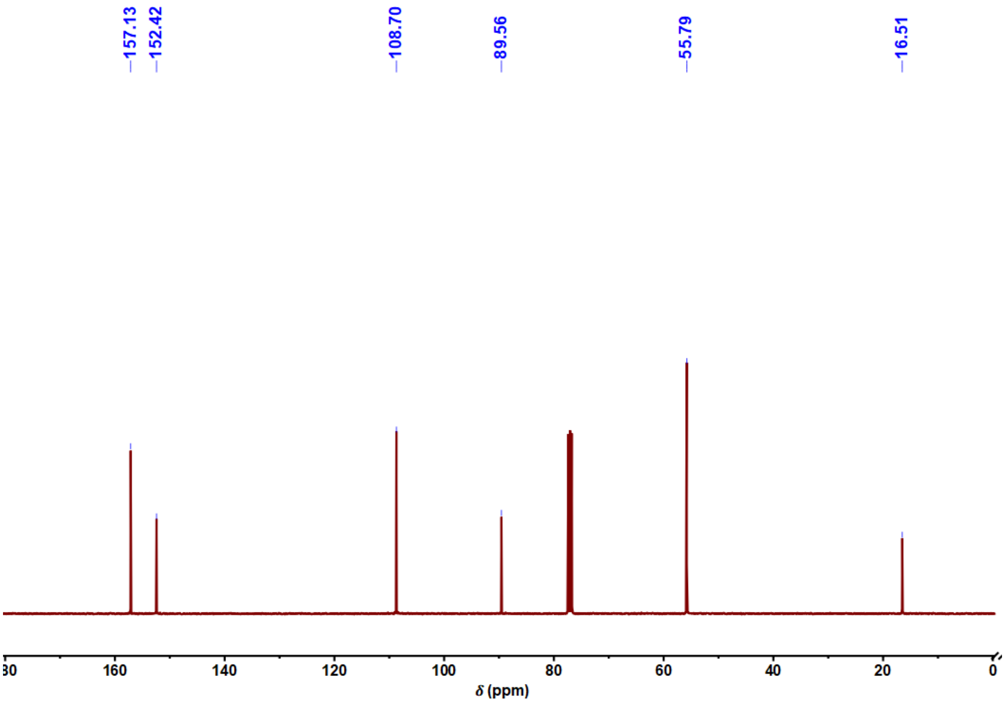

Figure S2. ^{13}C-NMR spectrum of **1** in $CDCl_3$ recorded at room temperature.

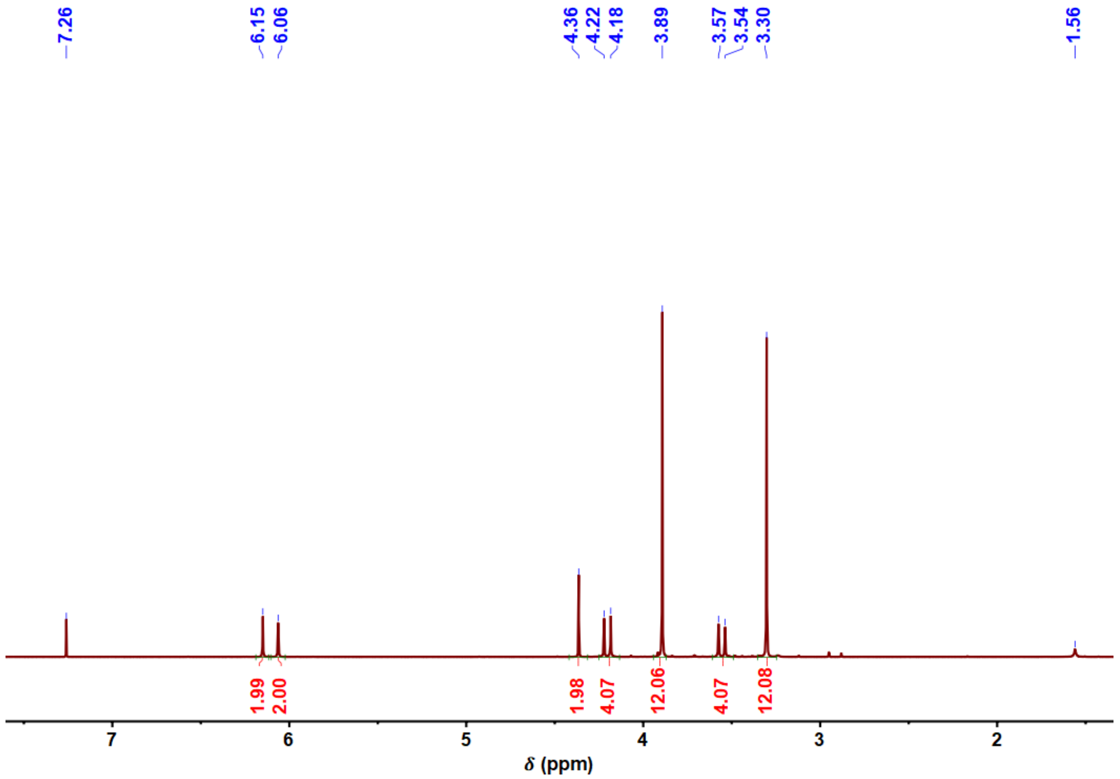

Figure S3. ^{1}H-NMR spectrum of **2** in $CDCl_3$ recorded at room temperature.

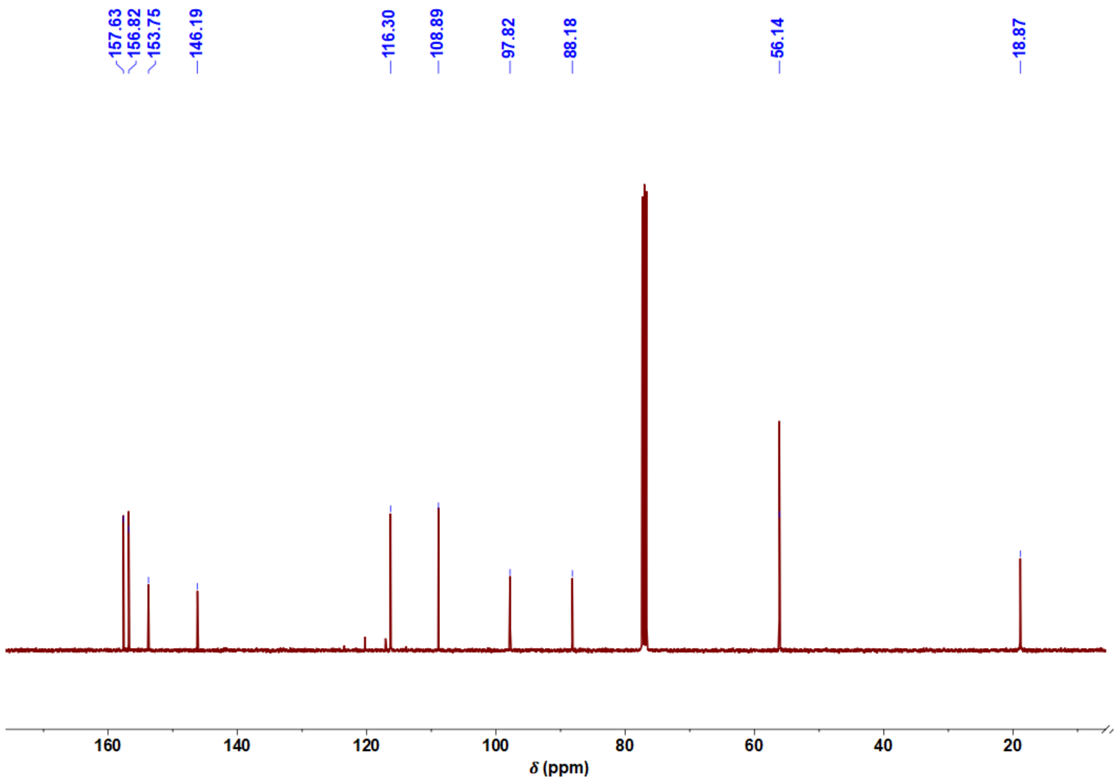

Figure S4. ^{13}C-NMR spectrum of **2** in $CDCl_3$ recorded at room temperature.

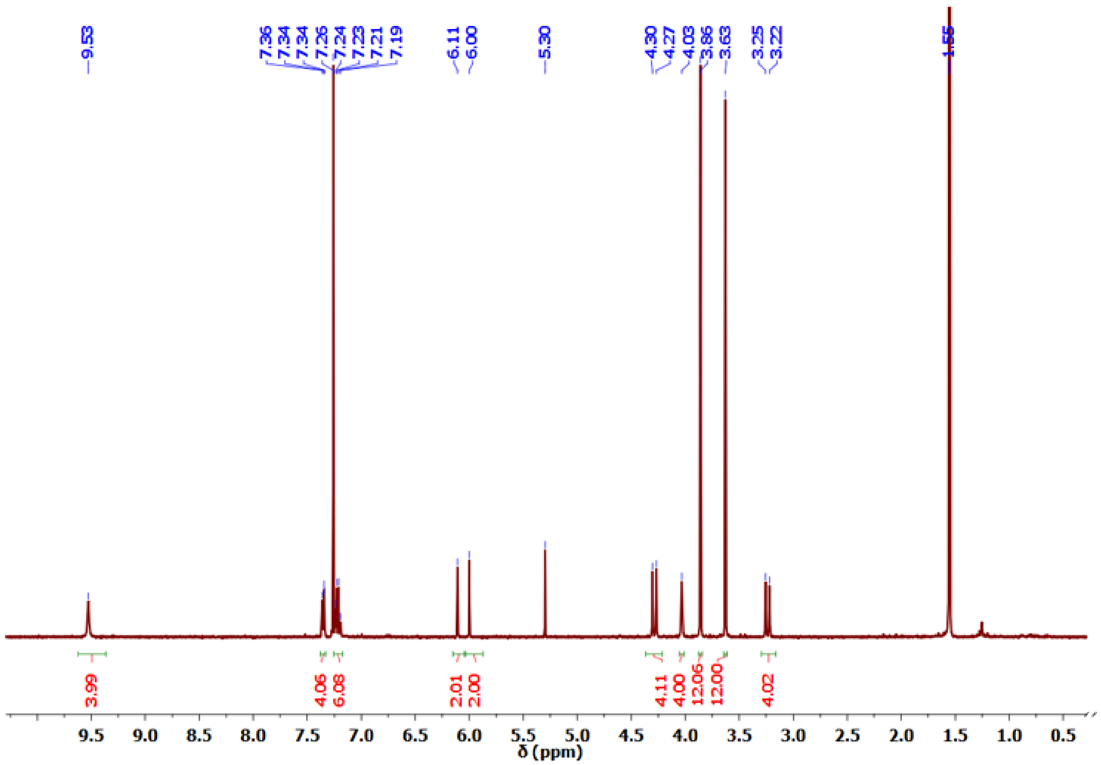

Figure S5. ^{1}H-NMR spectrum of **3** (atropisomer A) in $CDCl_3$ recorded at room temperature.

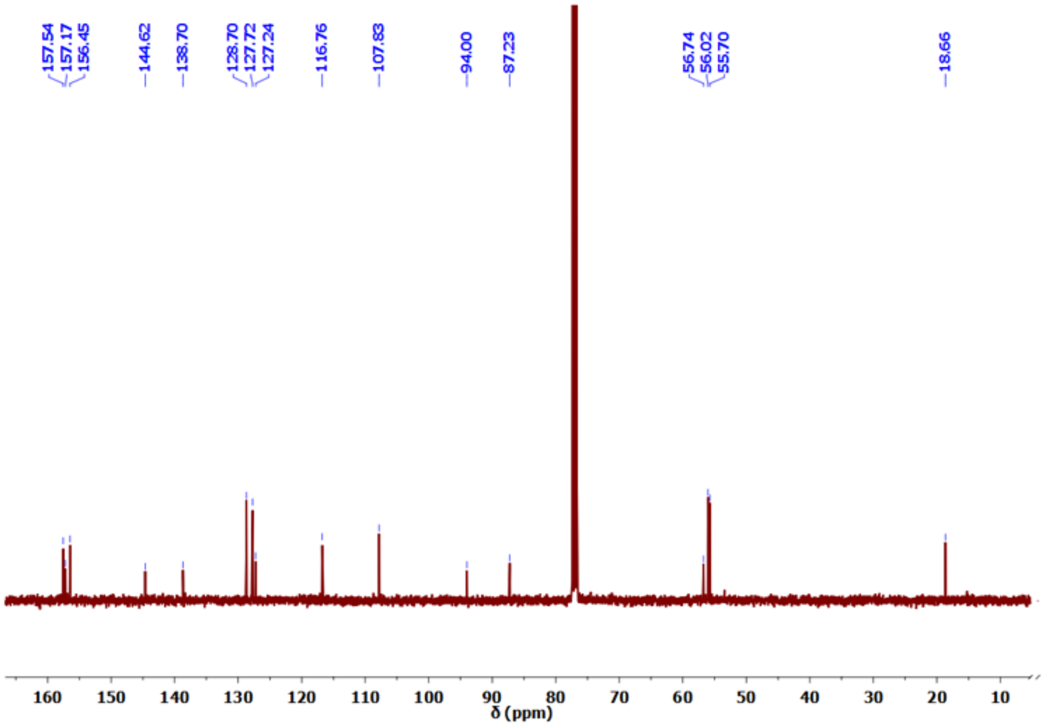

Figure S6. ^{13}C-NMR spectrum of **3** (atropisomer A) in $CDCl_3$ recorded at room temperature.

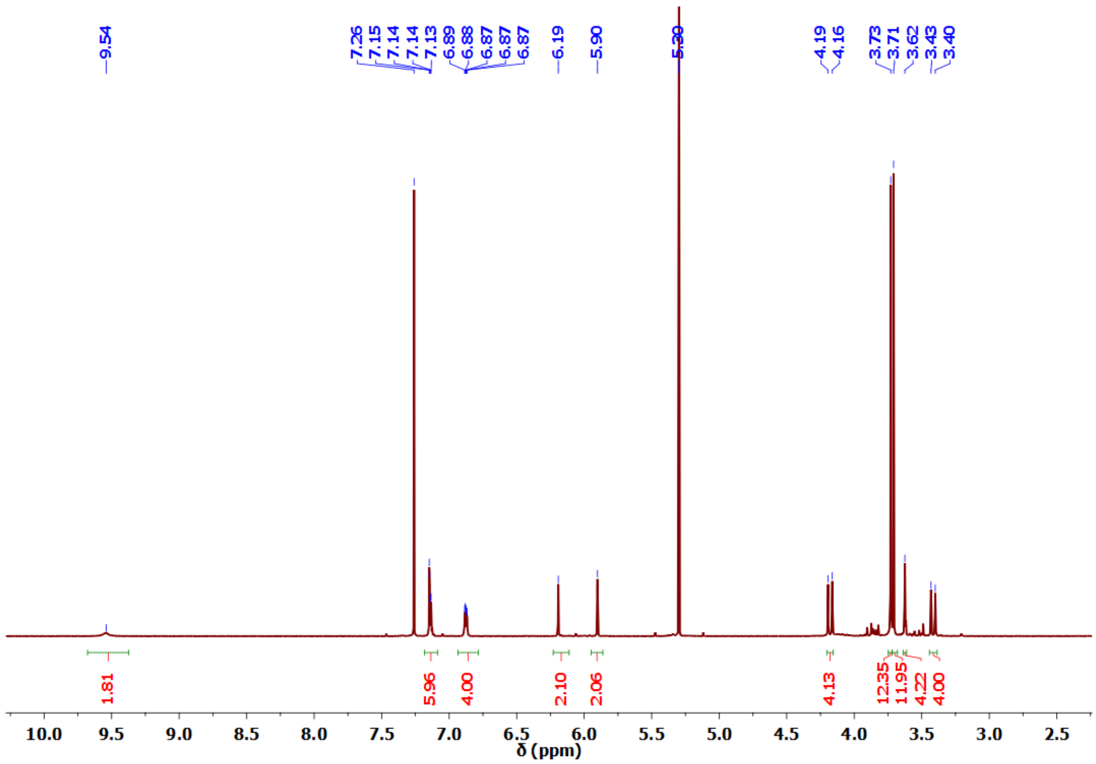

Figure S7. ^{1}H-NMR spectrum of **3** (atropisomer B) in $CDCl_3$ recorded at room temperature.

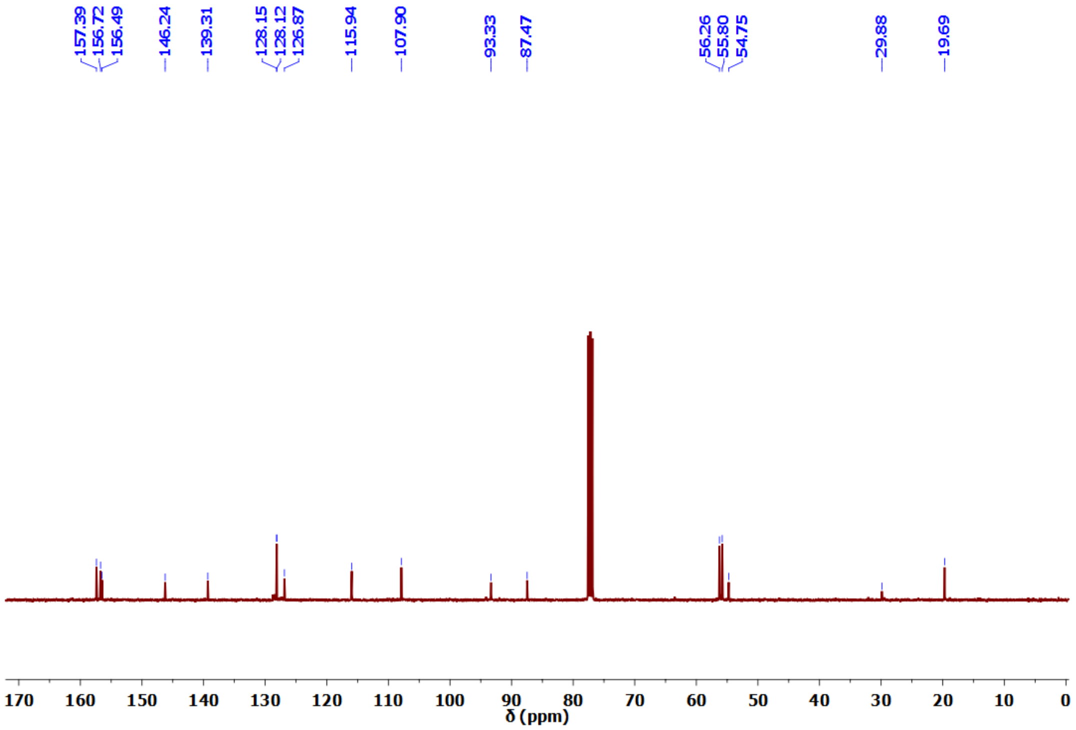

Figure S8. ^{13}C-NMR spectrum of **3** (atropisomer B) in $CDCl_3$ recorded at room temperature.

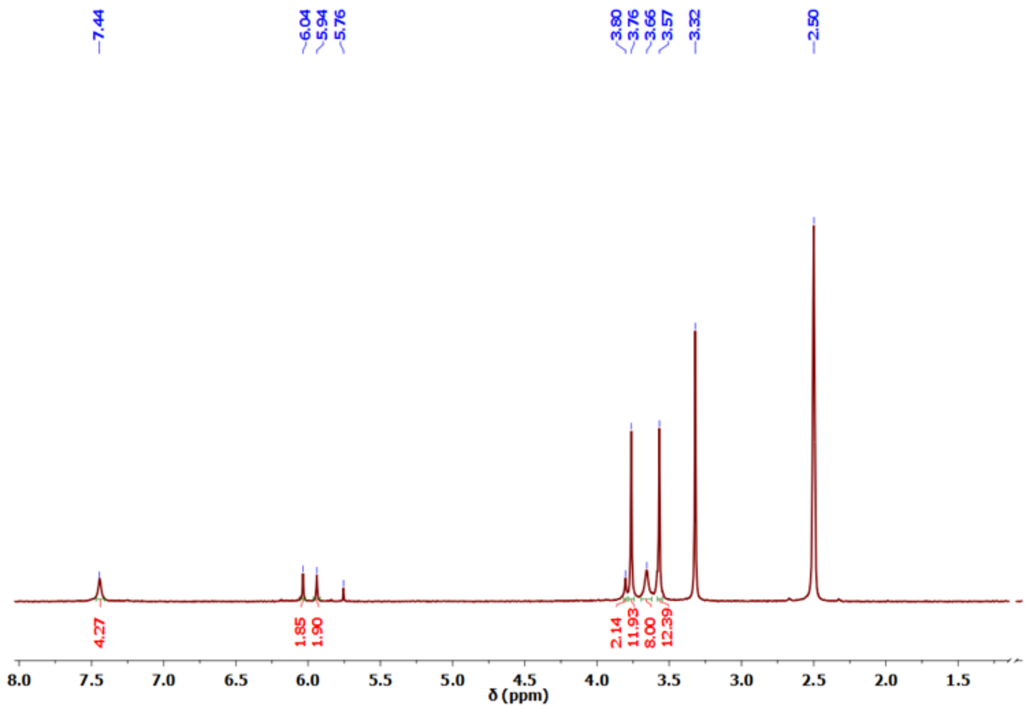

Figure S9. ^{1}H-NMR spectrum of **4** in DMSO-d_6 recorded at room temperature.

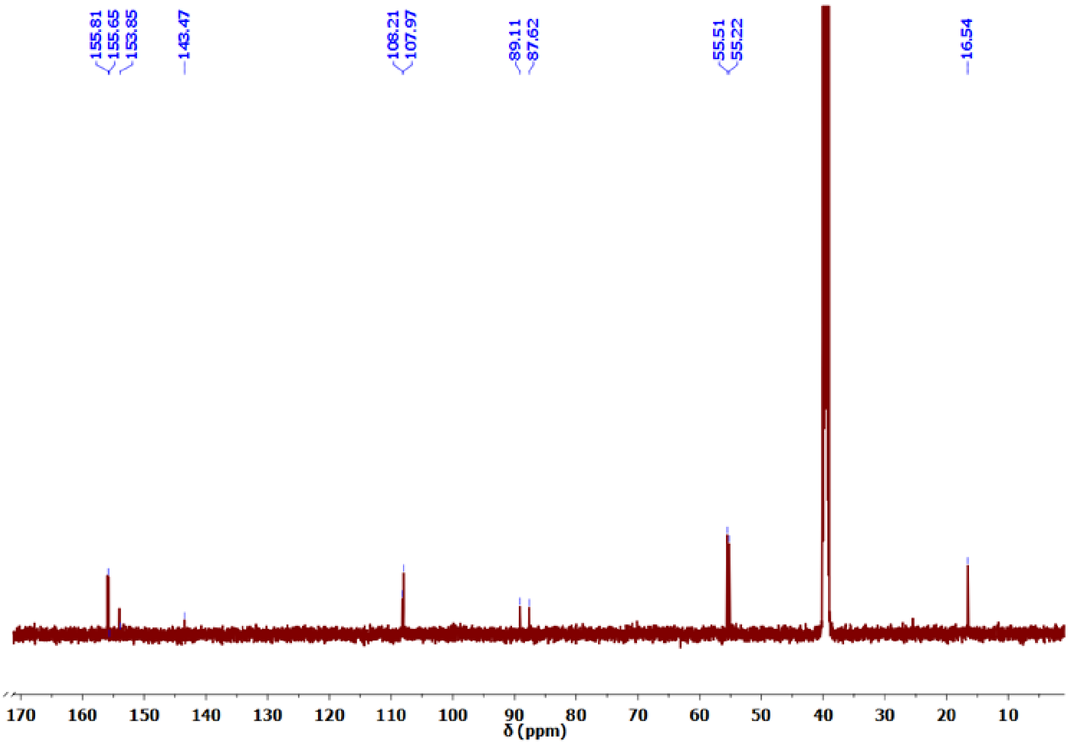

Figure S1. ^{13}C-NMR spectrum of **4** in DMSO-d_6 recorded at 373K, due to insufficient solubility at room temperature.

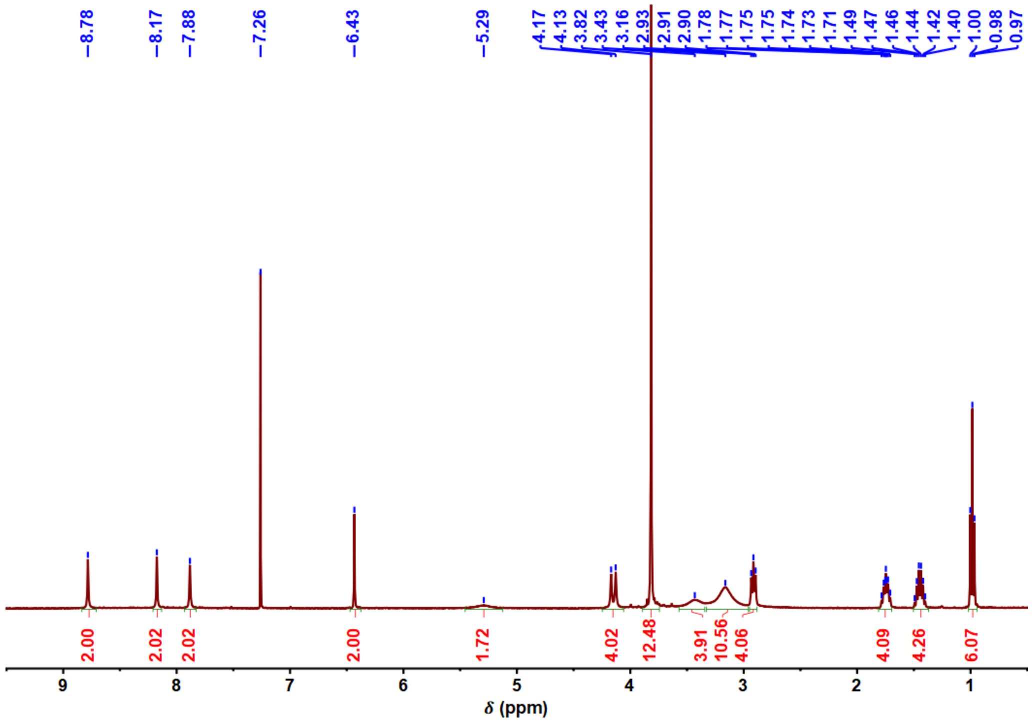

Figure S2. ^{1}H-NMR spectrum of **5** in $CDCl_3$ recorded at room temperature.

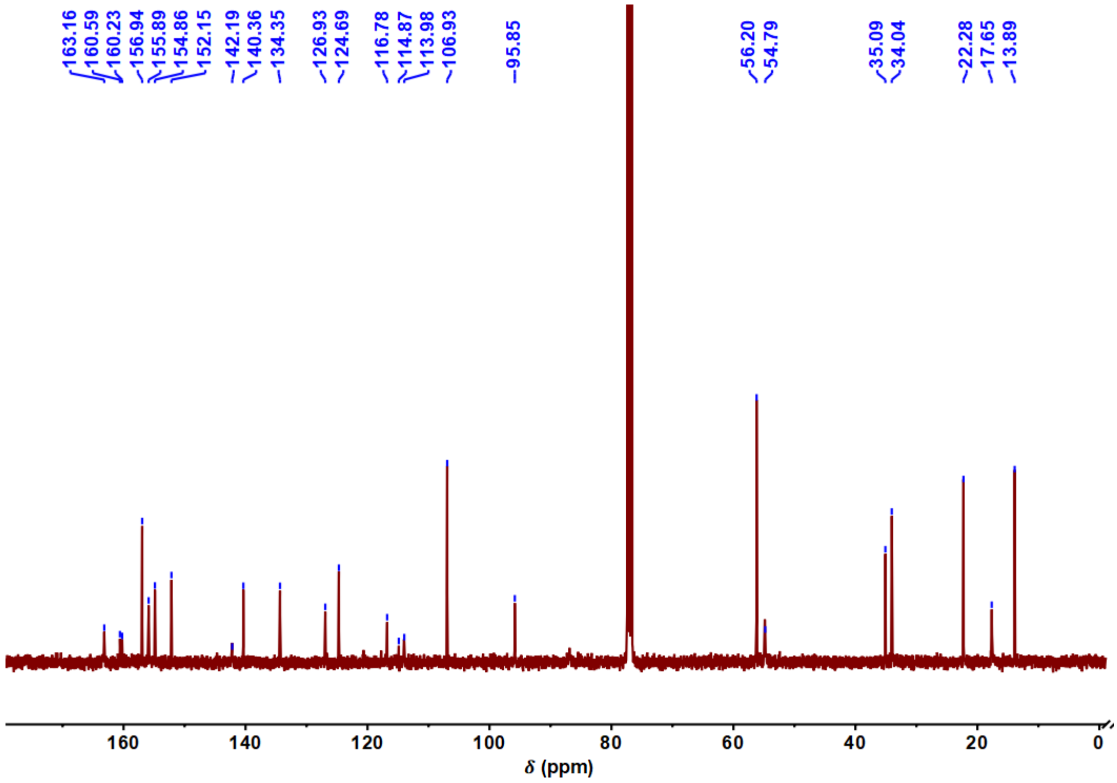

Figure S3. ^{13}C-NMR spectrum of **5** in $CDCl_3$ recorded at room temperature.

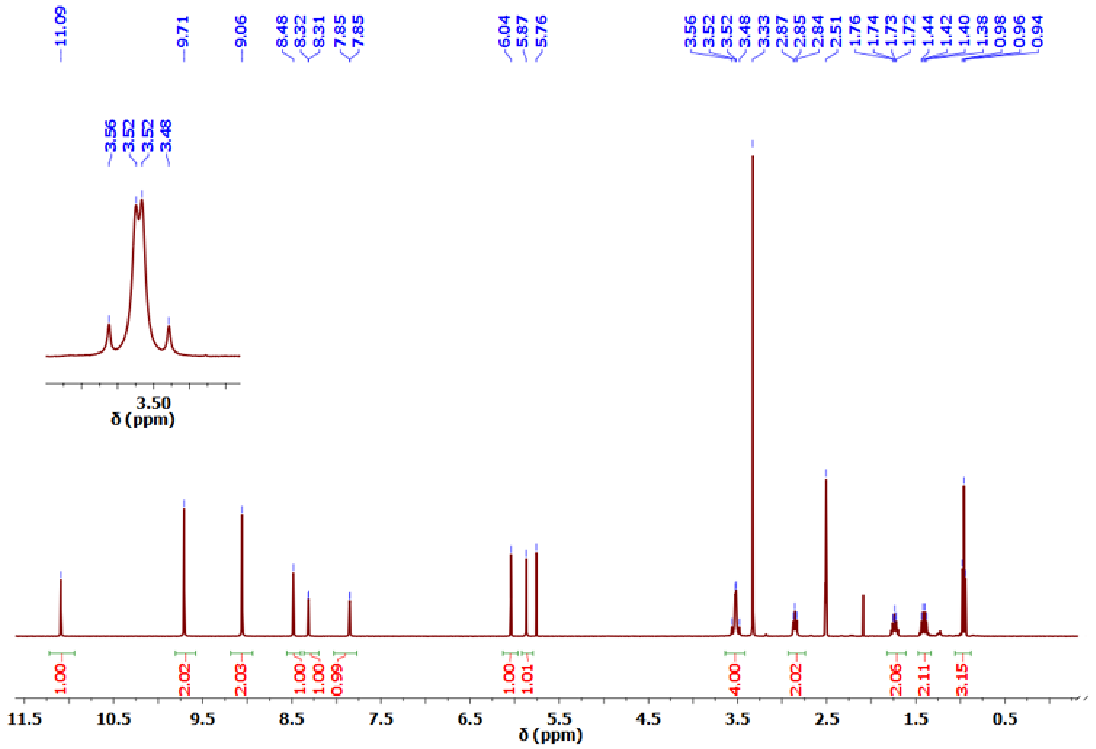

Figure S4. ^{1}H-NMR spectrum of **6** in DMSO-d_6 recorded at room temperature

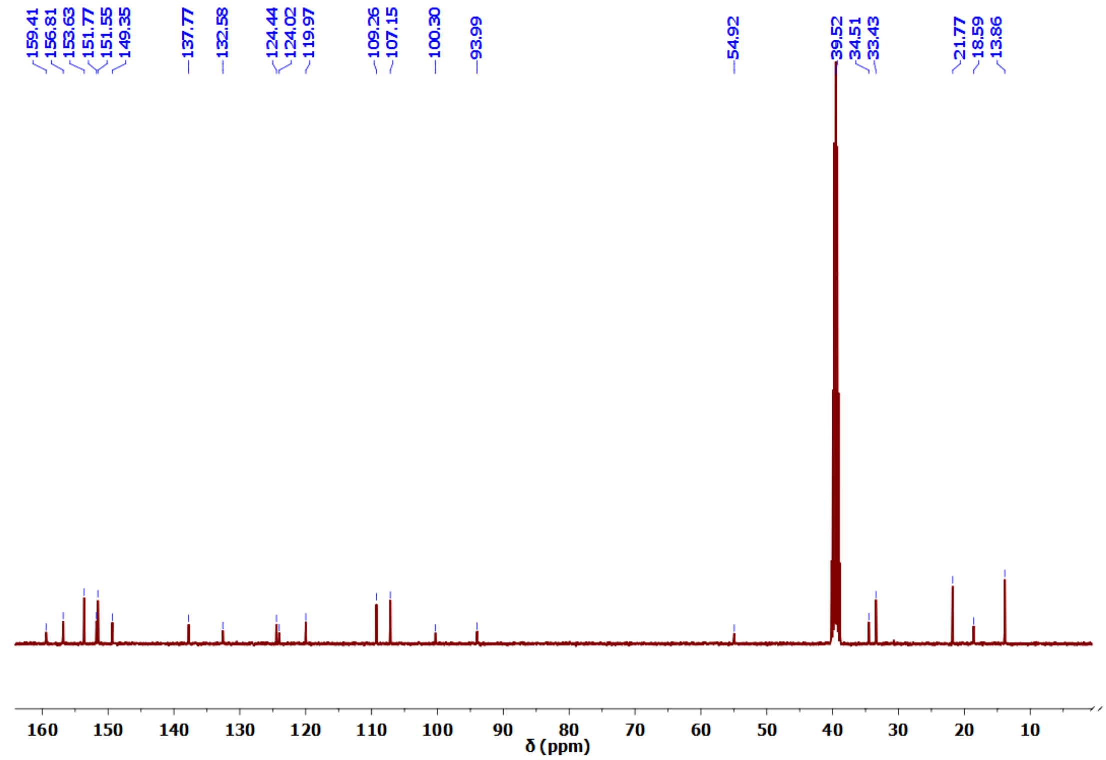

Figure S5. ^{13}C-NMR spectrum of **6** in DMSO-d_6 recorded at room temperature

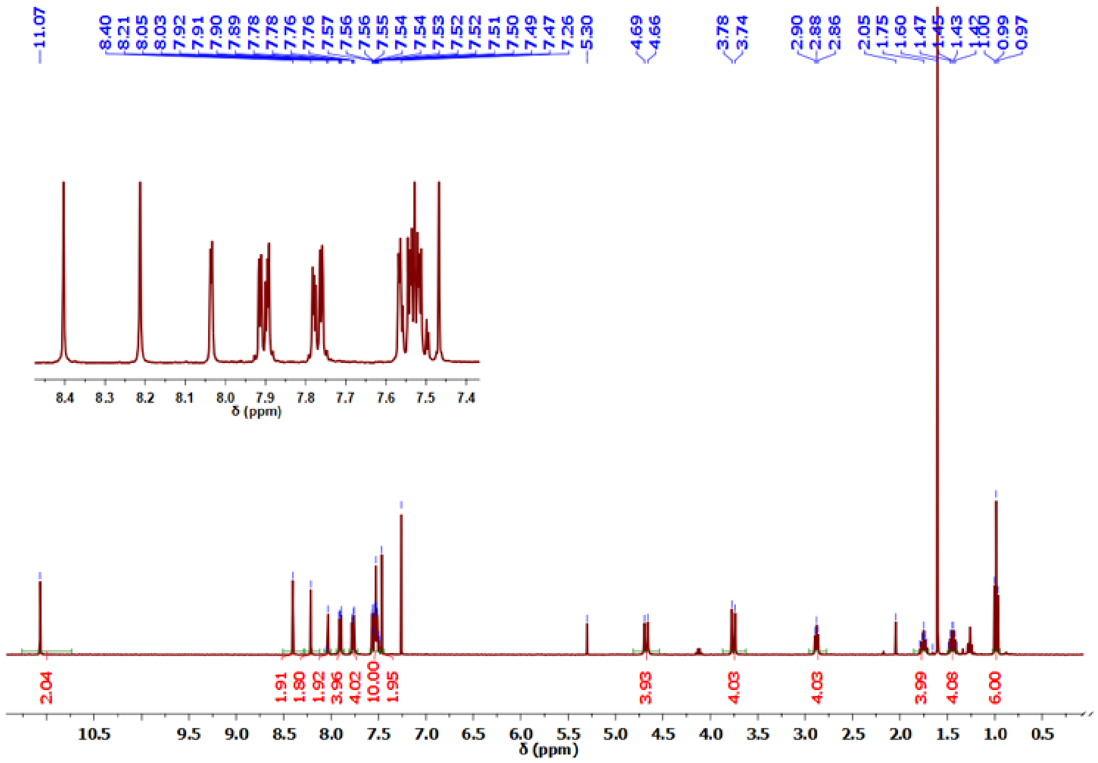

Figure S6. ^{1}H-NMR spectrum of **7** in $CDCl_3$ recorded at room temperature.

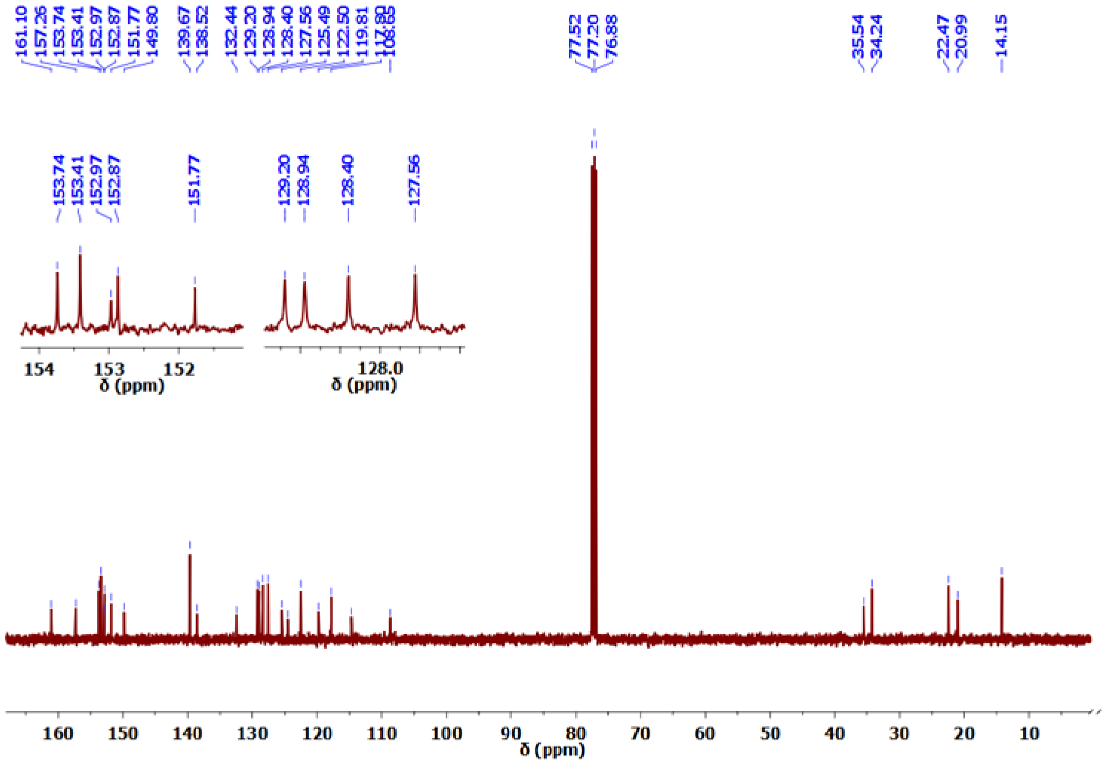

Figure S7. ^{13}C-NMR spectrum of **7** in $CDCl_3$ recorded at room temperature.

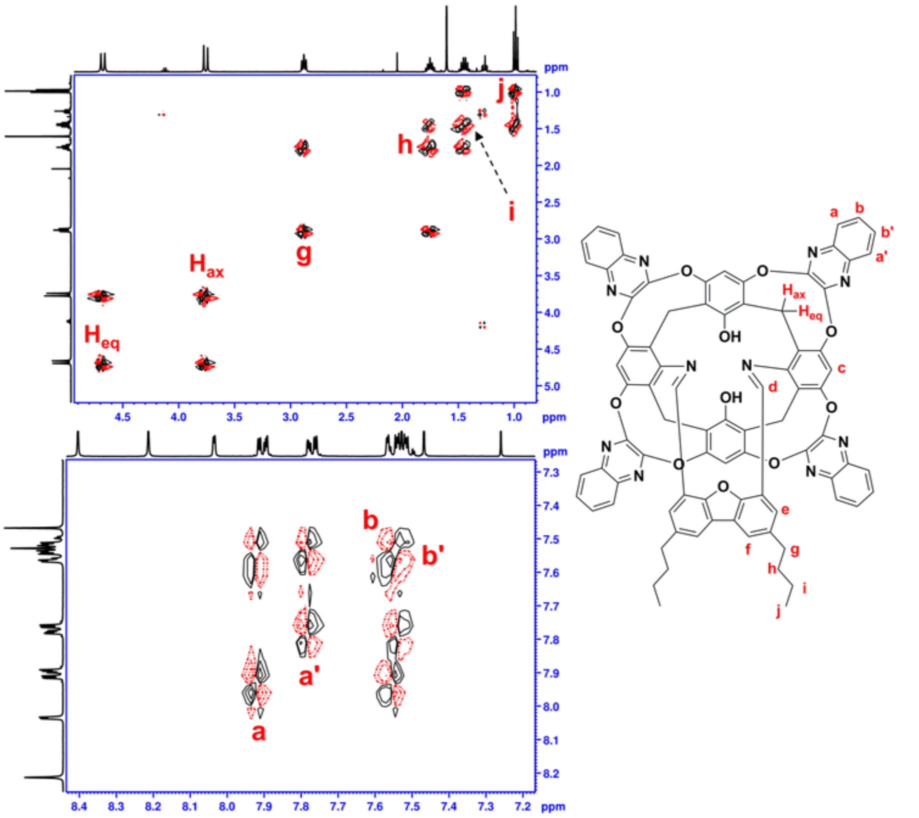

Figure S8. ^{1}H-^{1}H COSY NMR spectrum of **7** in $CDCl_3$ recorded at room temperature.

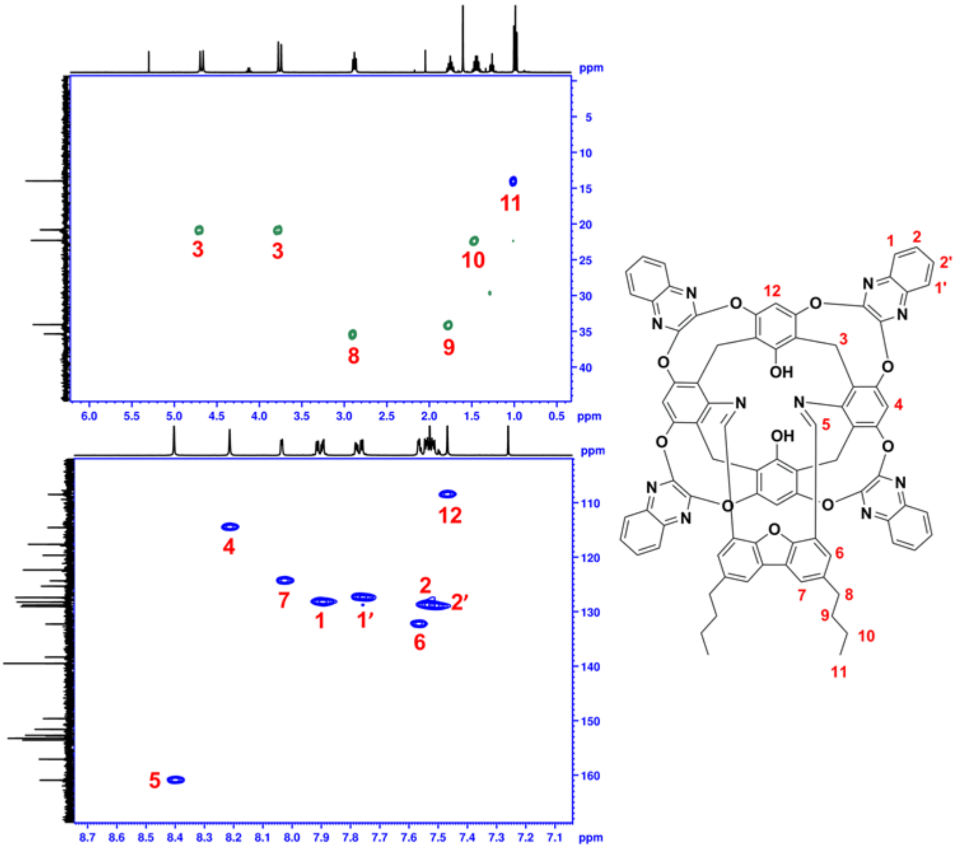

Figure S18. ^{1}H-^{13}C HSQC NMR spectrum of **7** in $CDCl_3$ recorded at room temperature.

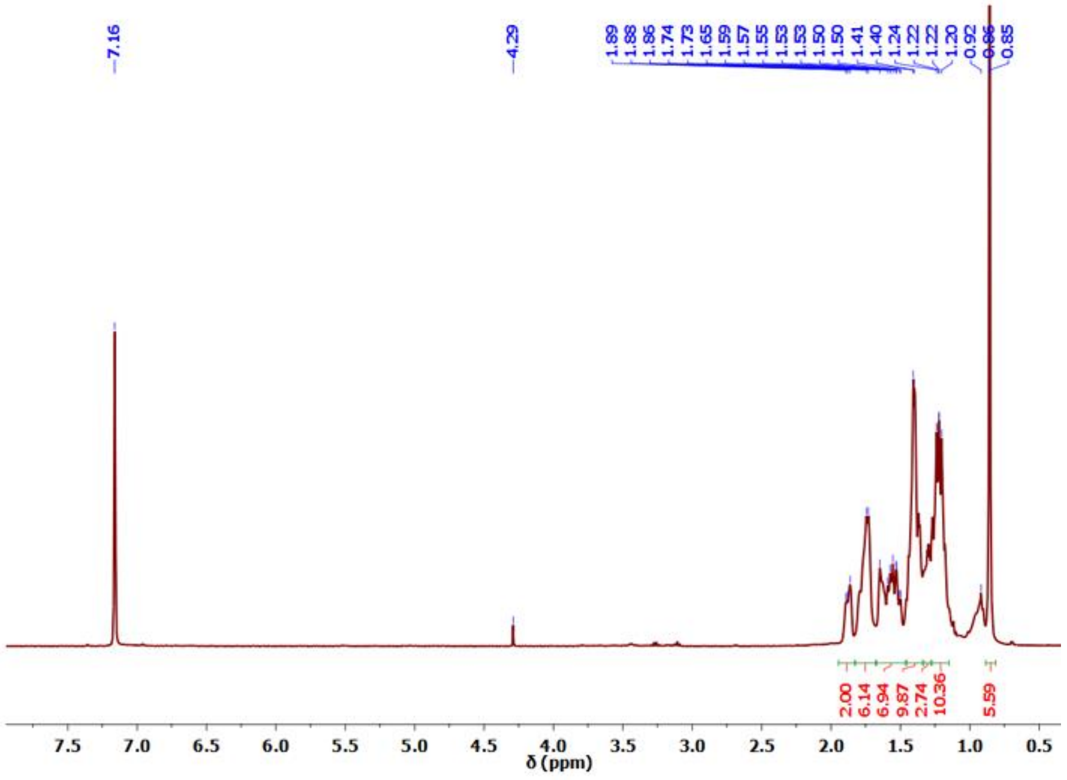

Figure S19. ^{1}H-NMR spectrum of **13** in C_6D_6 recorded at room temperature.

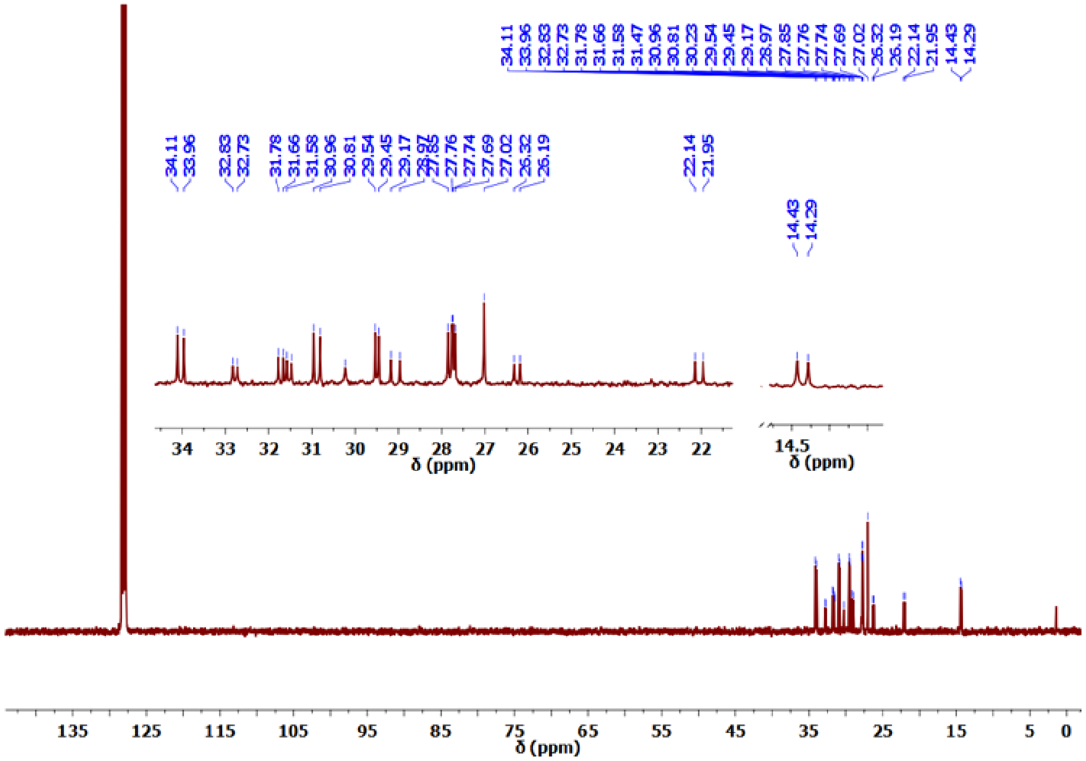

Figure S9. ^{13}C-NMR spectrum of **13** in C_6D_6 recorded at room temperature.

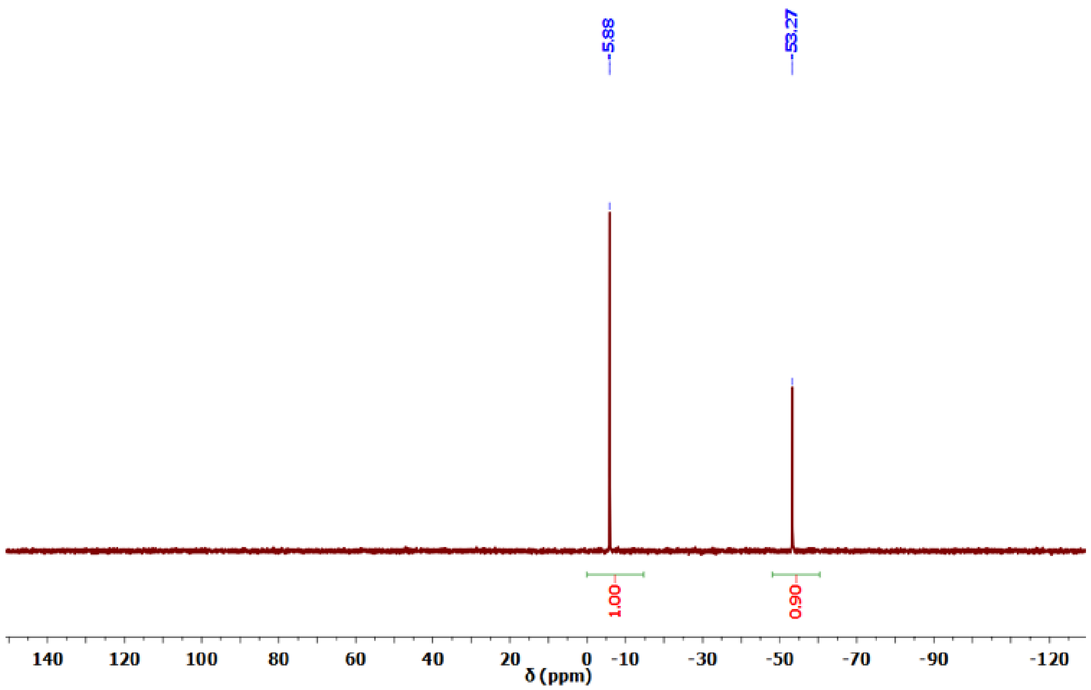

Figure S10. ^{31}P NMR (^{1}H decoupled) spectrum of **13** in C_6D_6 recorded at room temperature.

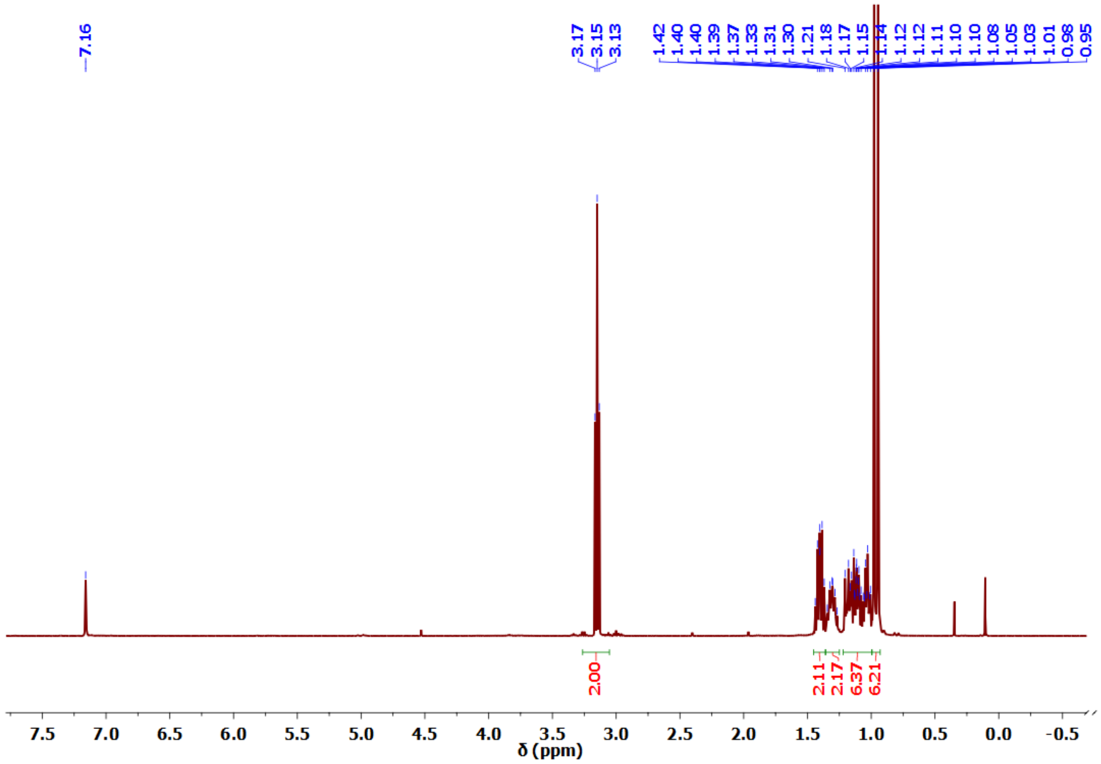

Figure S11. ^{1}H-NMR spectrum of **14** in C_6D_6 recorded at room temperature.

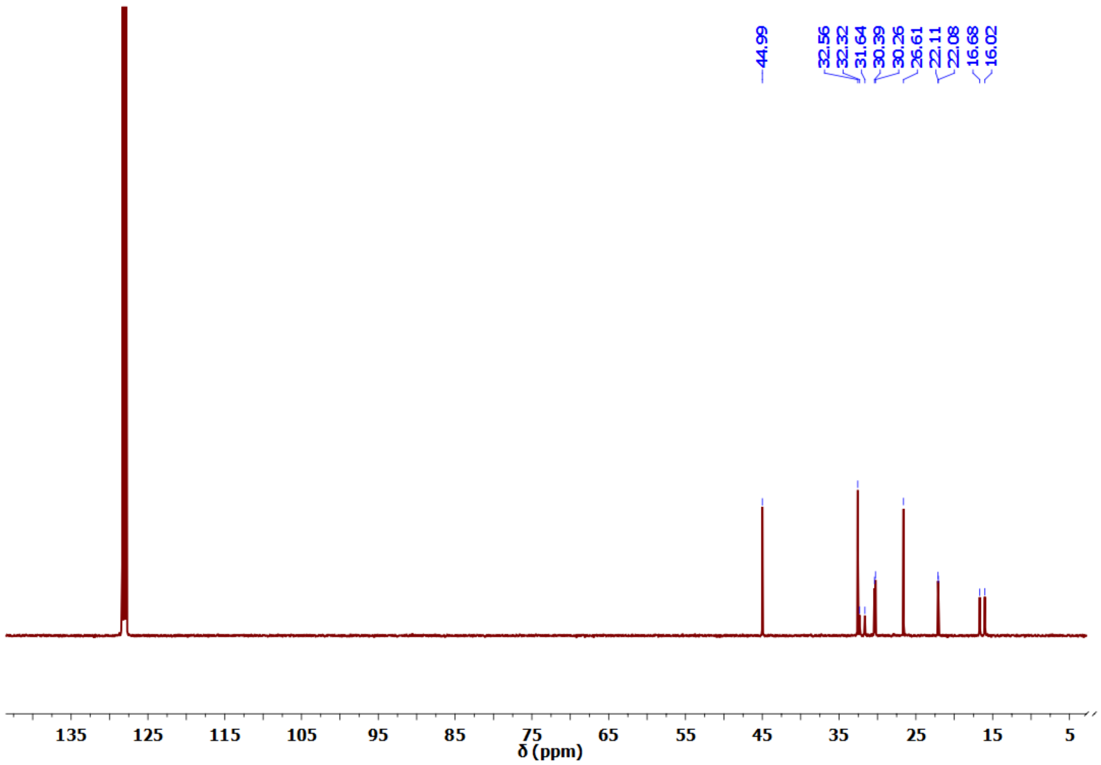

Figure S12. ^{13}C-NMR spectrum of **14** in C_6D_6 recorded at room temperature.

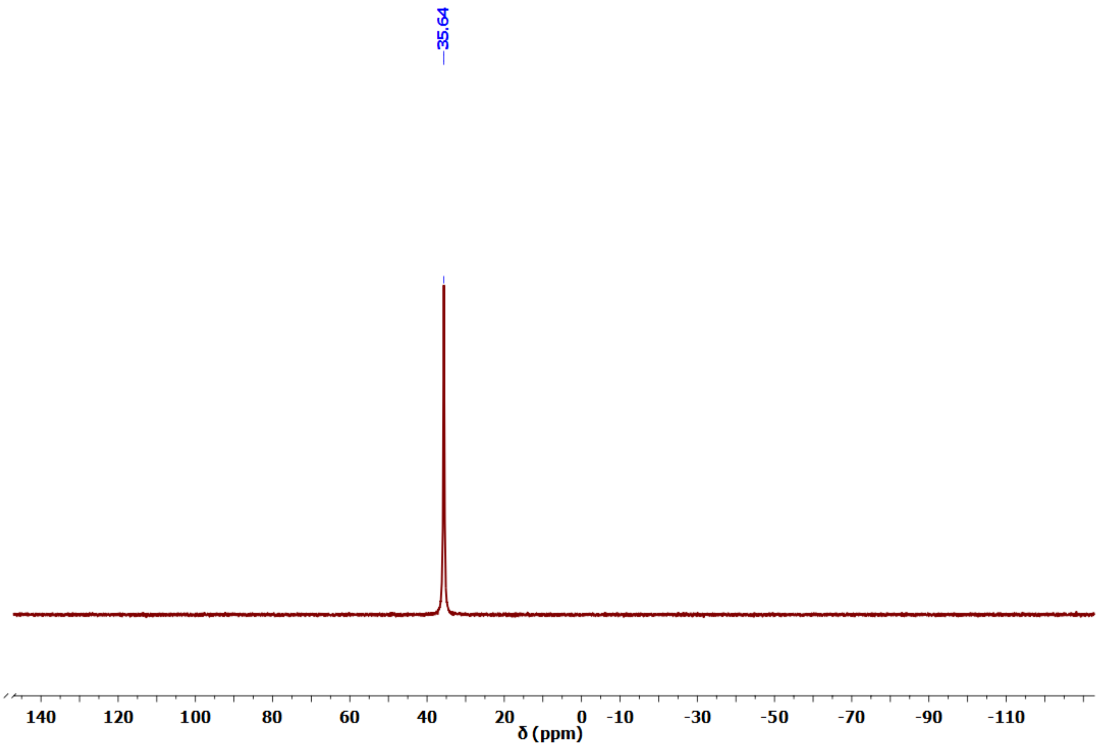

Figure S13. ^{31}P NMR (^{1}H decoupled) spectrum of **14** in C_6D_6 recorded at room temperature.

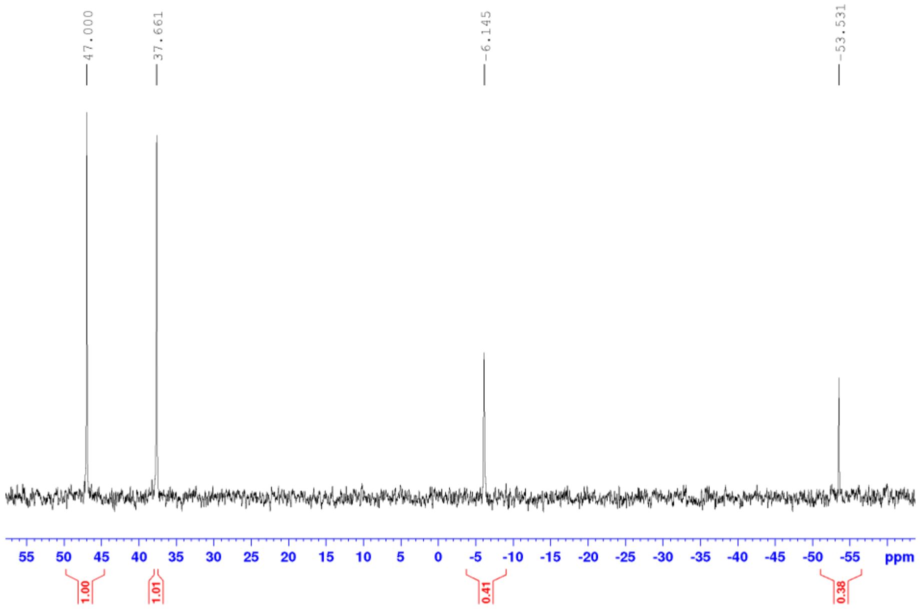

Figure S25. ^{31}P-NMR spectrum of **13** in 1,2-dichloroethane, recorded at room temperature in a sealed J. Young tube under N_2, after the addition of sPhIO (1 molar equivalent).

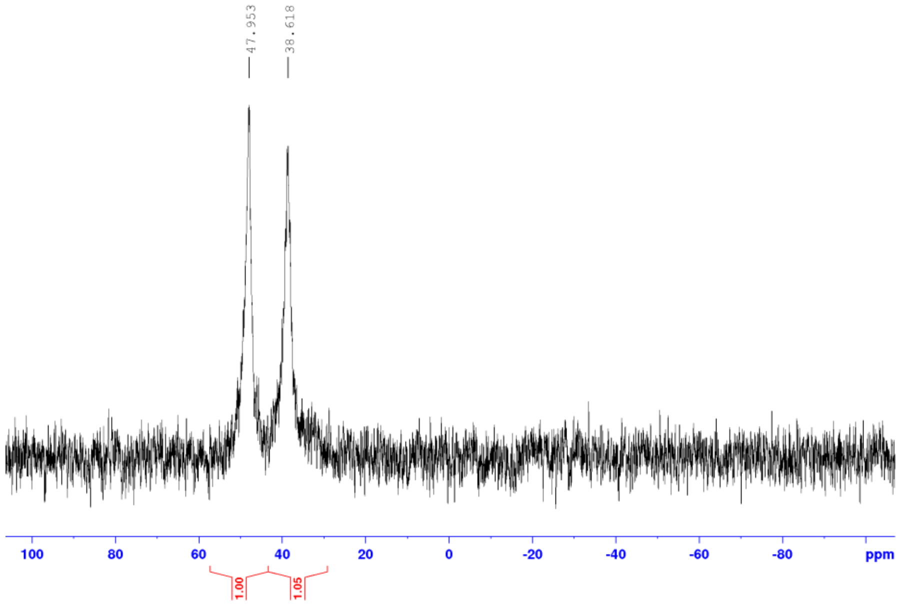

Figure S26. ^{31}P-NMR spectrum of **13** in 1,2-dichloroethane, recorded at room temperature in a sealed J. Young tube under N_2, after the addition of MnO_2 (excess).

5. EPR spectrum of complex 8

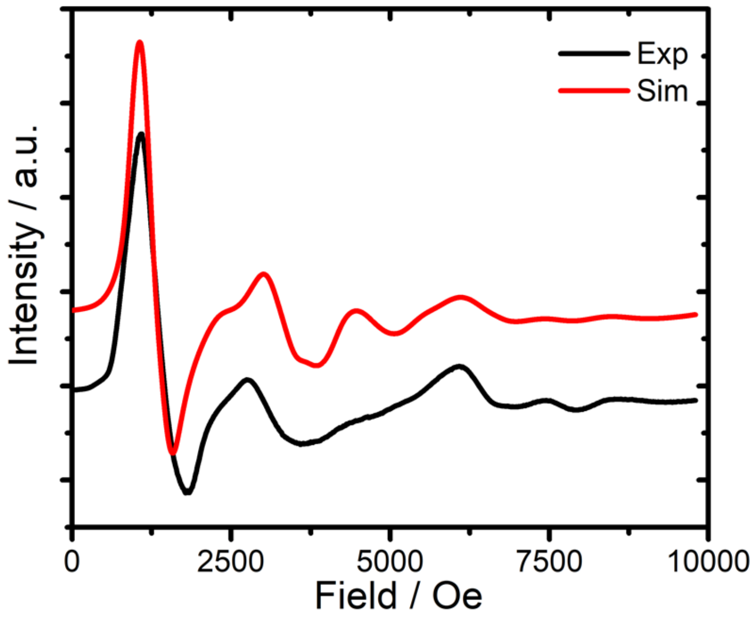

Figure S27. X-band CW-EPR spectra of complex **8** (ground solid) at 5 K. The experimental spectrum (black trace) is overlaid with the simulated spectrum (red trace). Spin Hamiltonian parameters used for the simulations are specified in **Table S6** (see section 9).

6. XPS spectra of complexes 8-11.

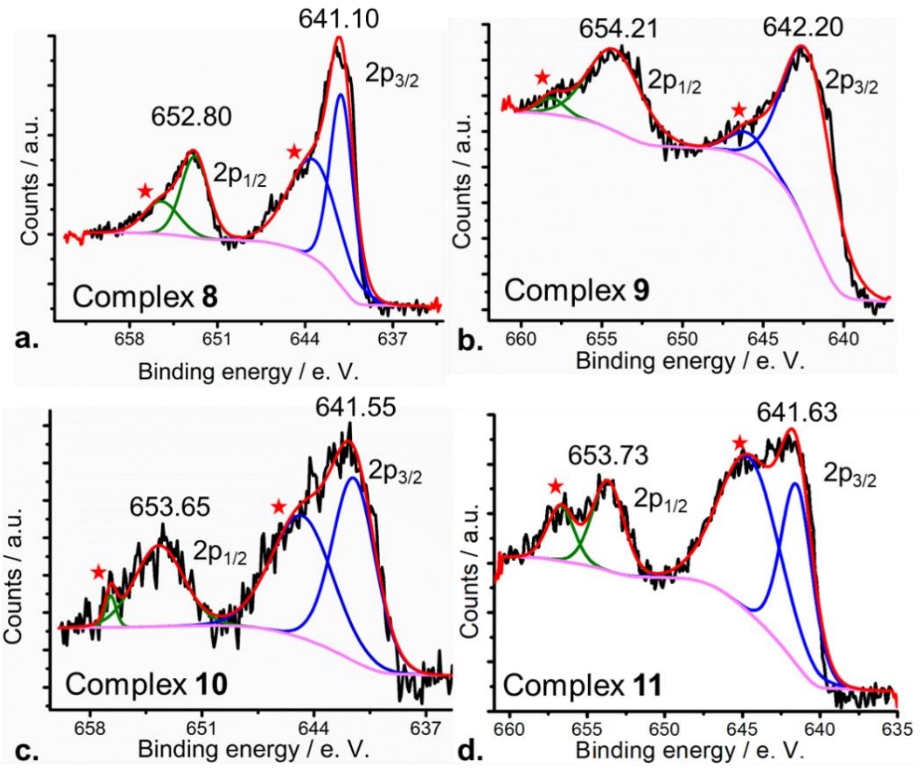

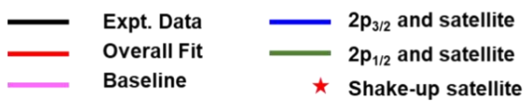

Figure S28. XPS spectra of the Mn 2p photoelectrons in complexes **8** (a), **9** (b), **10** (c), and **11** (d).

Table S1. Binding energies of the Mn (2p) electrons in complexes **8-11** from fitting the XPS data.

Complex	$2p_{3/2}$ (eV)	$2p_{1/2}$ (eV)
**8**	641.10	652.80
**9**	642.20	654.20
**10**	641.55	653.65
**11**	641.63	653.73

7. UV-vis spectra and their kinetic analysis

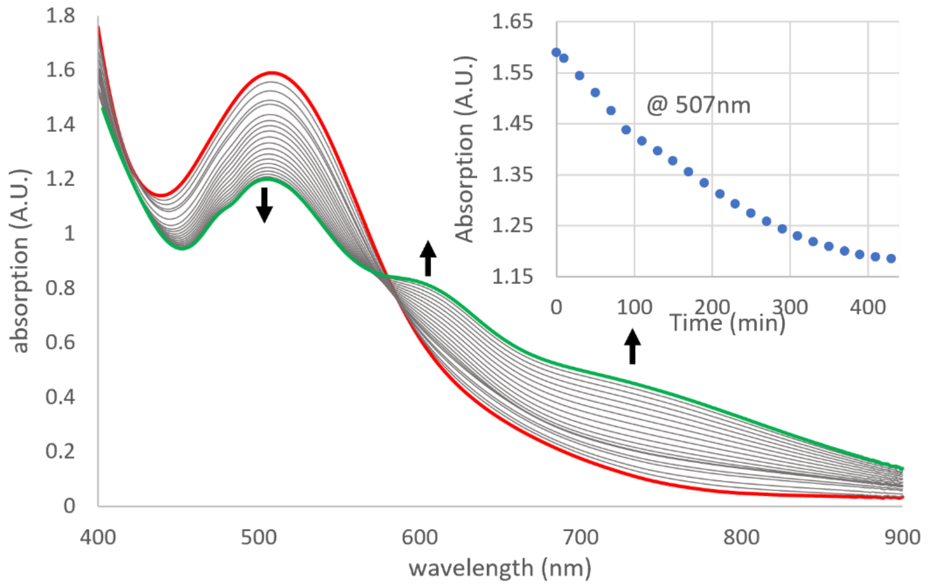

Figure S29 – The spectral changes in the electronic absorption of **9** during the decay of the intermediate in 1,2-DCE at r.t. The inset shows the change in absorption at λ_{max} = 507 nm over time. The black arrows indicate the direction of change.

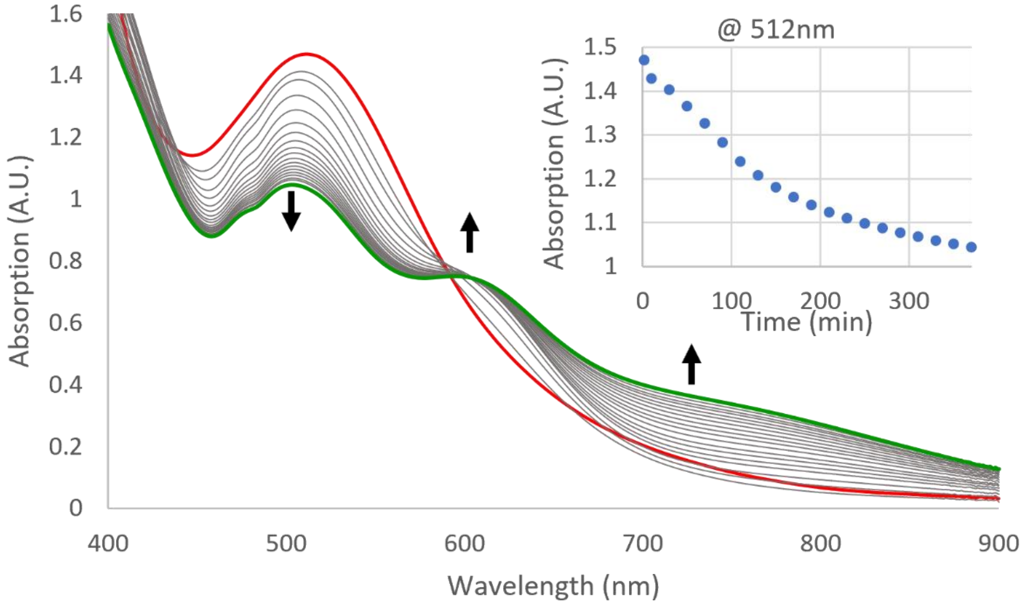

Figure S30 - The spectral changes in the electronic absorption of **9** during the decay of the intermediate in $CHCl_3$ at r.t. The inset shows the change in absorption at λ_{max} = 507nm in time. The black arrows indicate the direction of change.

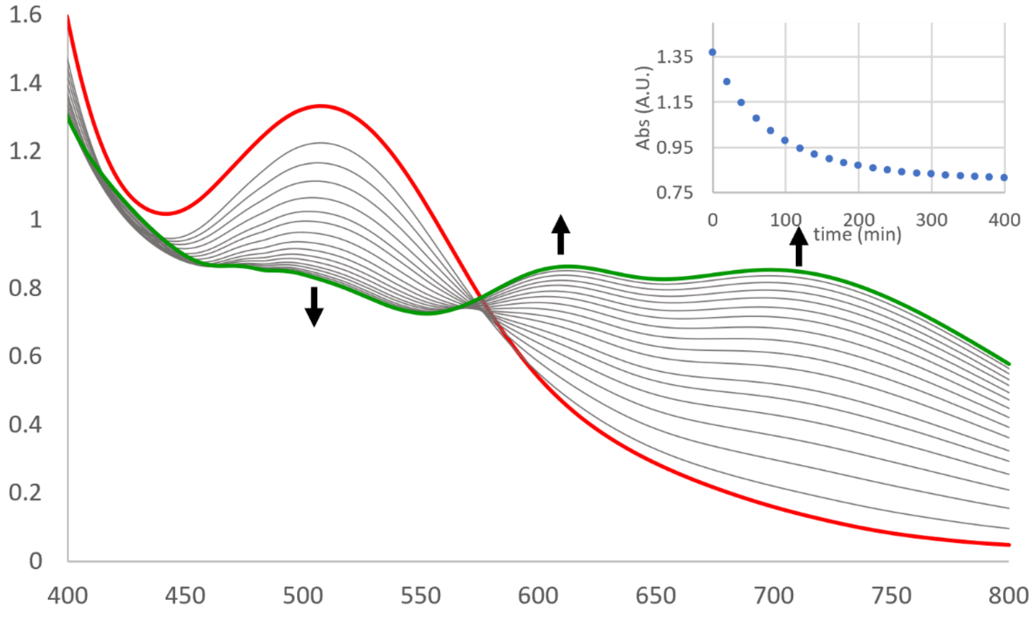

Figure S31 - The spectral changes in the electronic absorption of **9** during the decay of the intermediate in DCM at r.t. The inset shows the change in absorption at λ_{max} = 507 nm in time. The black arrows indicate the direction of change.

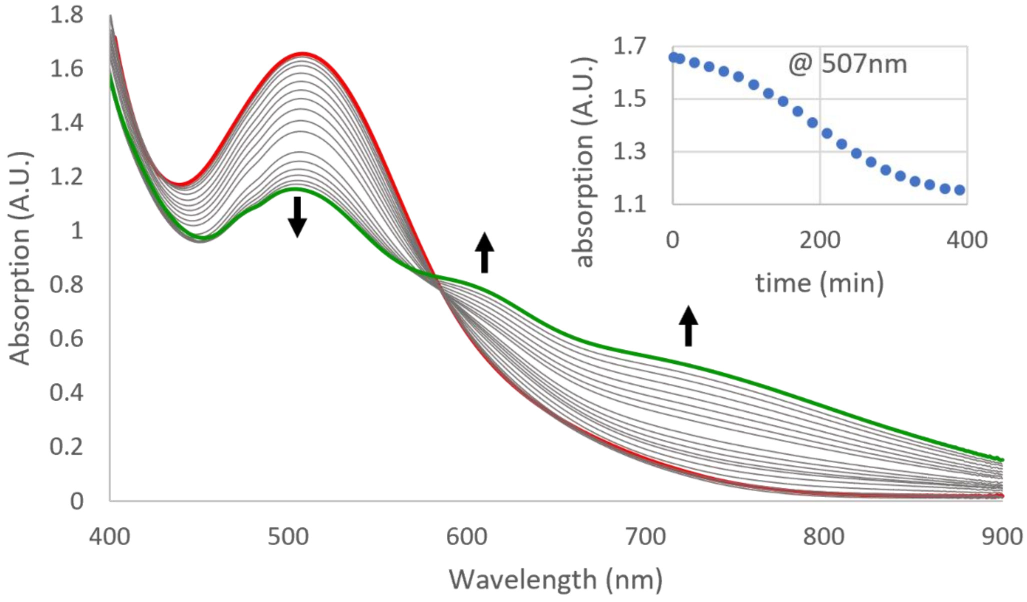

Figure S32 – The spectral changes in the electronic absorption of **9** in 1,2-DCE, after addition of 50 molar equivalents of 9,10-DHA at r.t. The inset shows the change in absorption at λ_{max} = 507 nm in time.The black arrows indicate the direction of change.

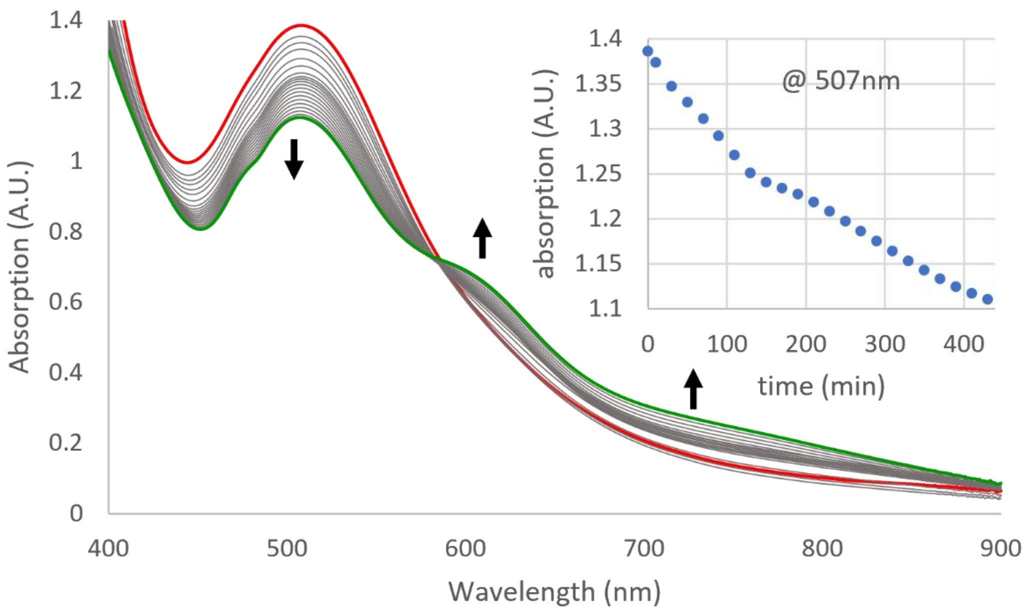

Figure S33 - The spectral changes in the electronic absorption of **9** in 1,2-DCE, after addition of 50 molar equivalents of Ph_3CH at r.t. The inset shows the change in absorption at λ_{max} = 507nm in time.The black arrows indicate the direction of change.

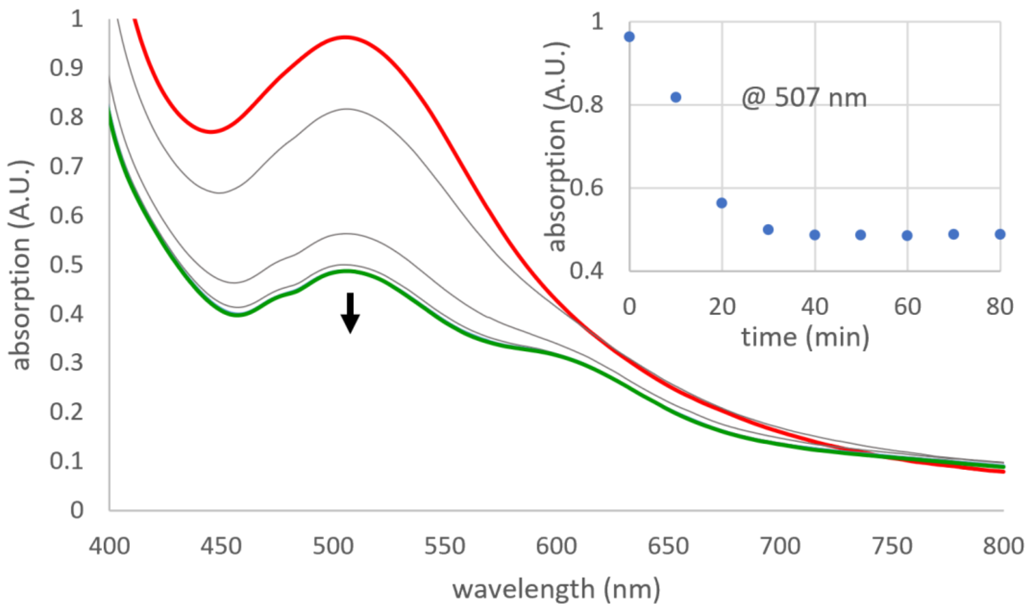

Figure S14 - The spectral changes in the electronic absorption of **9** in 1,2-DCE, after addition of 50 molar equivalents of 1,4-CHD at r.t. The inset shows the change in absorption at λ_{max} = 507nm in time.The black arrow indicates the direction of change.

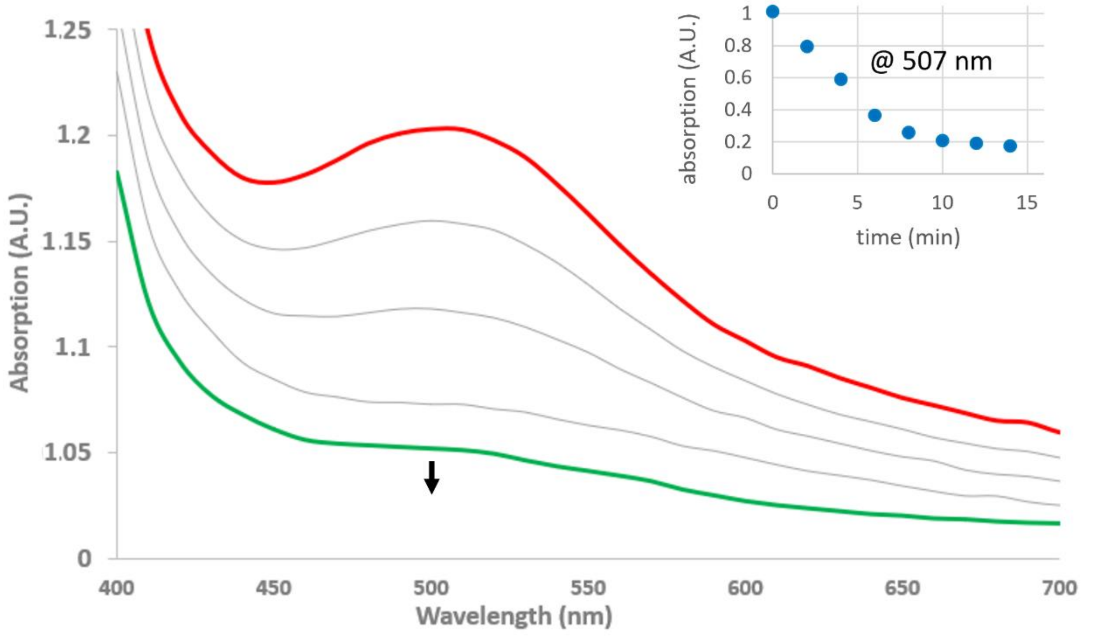

Figure S15 – The spectral changes in the electronic absorption of **9** in 1,2 DCE at r.t. upon addition of 50 molar equivalents of PMe_3. The inset shows the change in absorption at λ_{max} = 507nm in time. The black arrow indicates the direction of change.

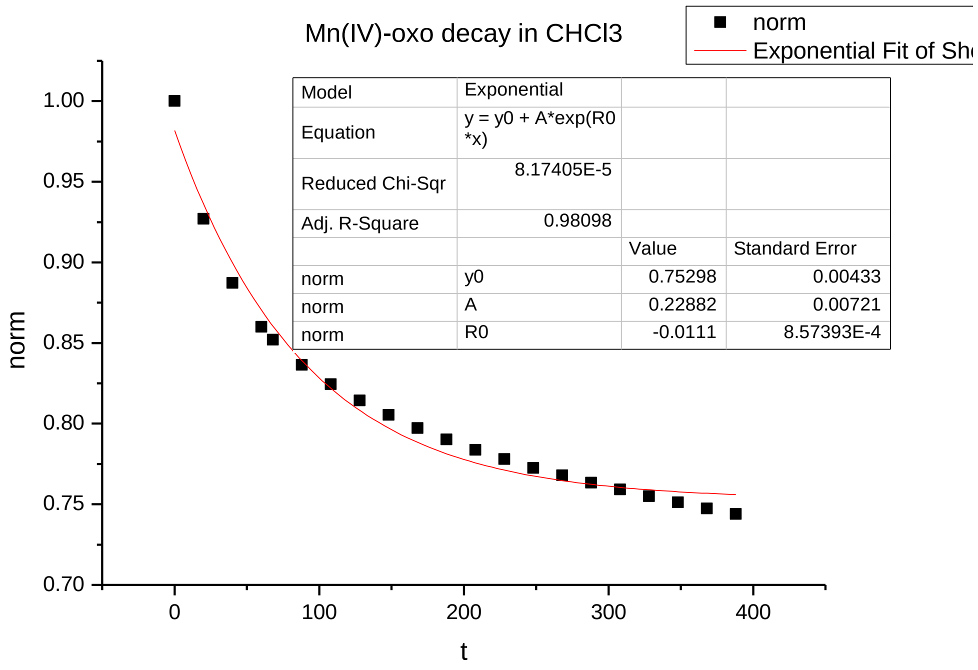

Figure S16 – The change in electronic absorption of **9** at λ_{max} = 507nm, during the decay of the intermediate in $CHCl_3$ at r.t. The red curve represents the best fit obtained.

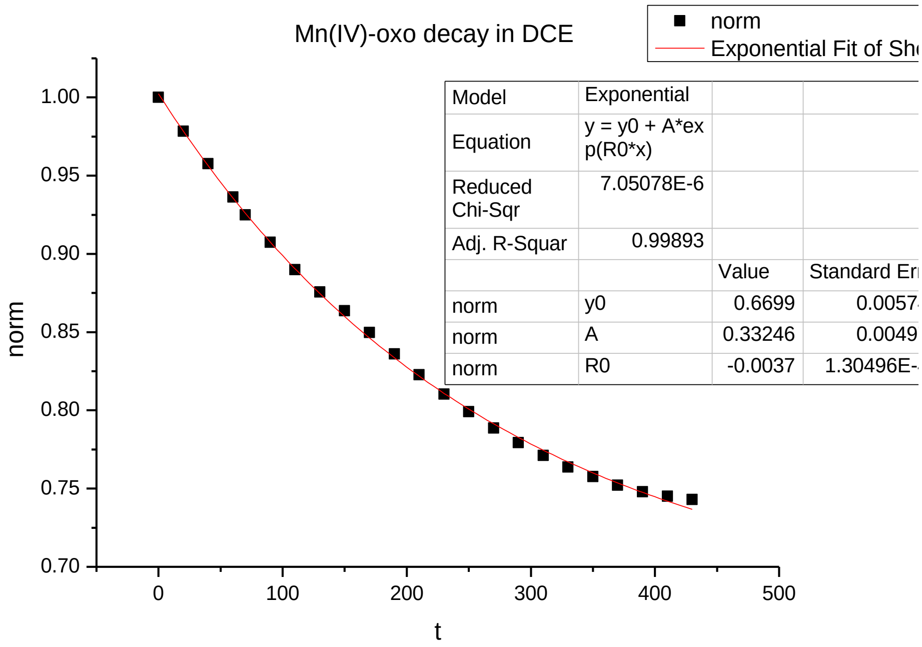

Figure S17 – The change in electronic absorption of **9** at λ_{max} = 507nm, during the decay of the intermediate in 1,2-DCE at r.t. The red curve represents the best fit obtained.

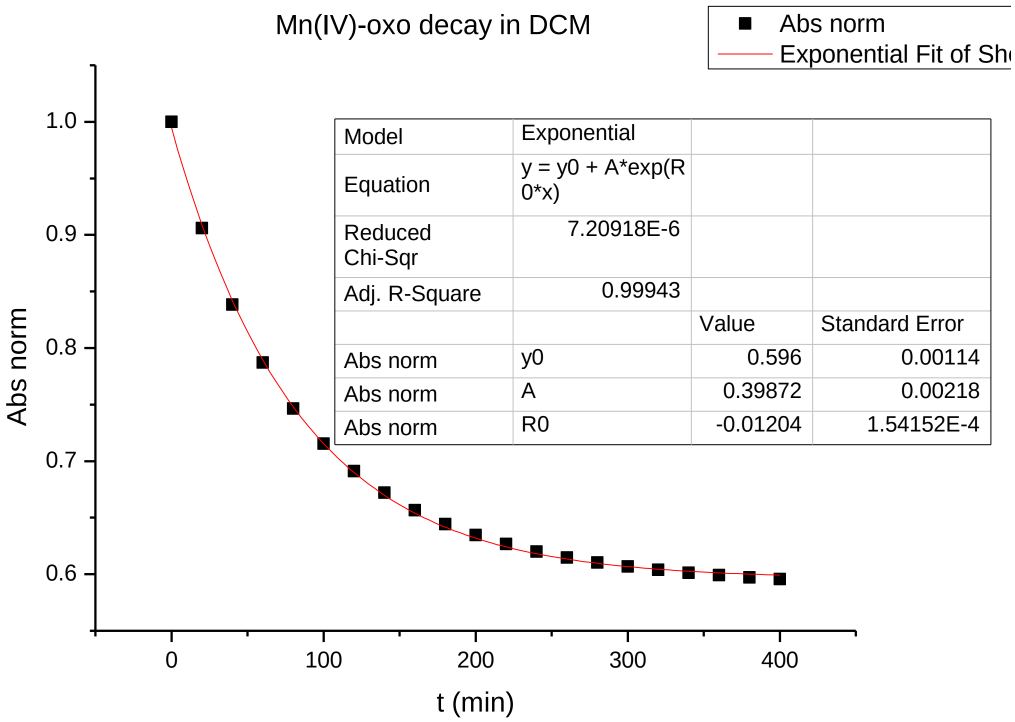

Figure S18 – The change in electronic absorption of **9** at λ_{max} = 507nm, during the decay of the intermediate in DCM at r.t. The red curve represents the best fit obtained.

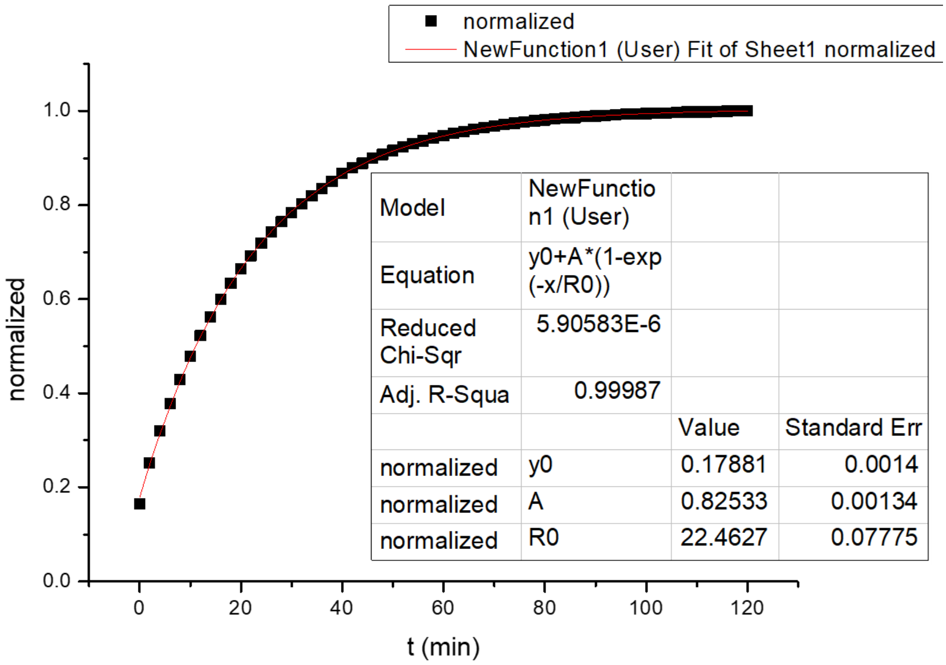

Figure S39 – The change in electronic absorption of **8** in DCM at λ_{max} = 507nm, after the addition of 1.5 molar equivalents of sPhIO at 241K. The red curve represents the best fit obtained.

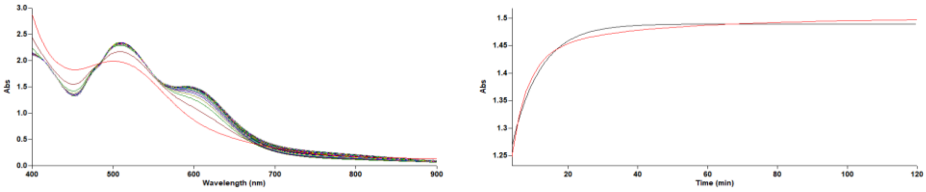

Figure S40. Left: The spectral changes observed in the UV-Vis spectra of **9** for the reaction with Ethylbenzene (105 molar equivalents) at room temperature in DCE; Right: Time-dependent absorbance at 600 nm (Black trace), and the best 1st order rate fit obtained (red trace).

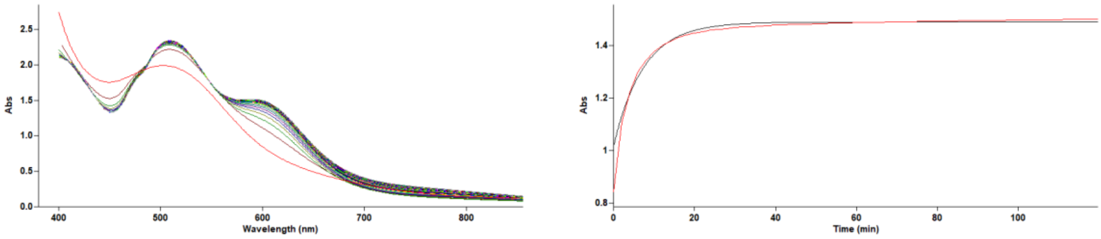

Figure S41. Left: The spectral changes observed in the UV-Vis spectra of **9** for the reaction with Ethylbenzene (158 molar equivalents) at room temperature in DCE; Right: Time-dependent absorbance at 600 nm (Black trace), and the best 1st order rate fit obtained (red trace).

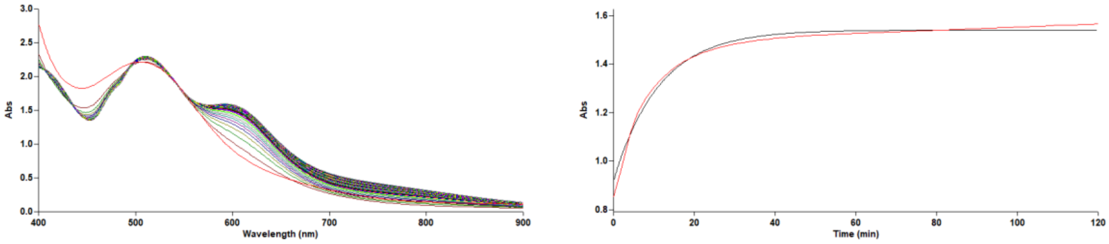

Figure S42. Left: The spectral changes observed in the UV-Vis spectra of **9** for the reaction with Ethylbenzene (210 molar equivalents) at room temperature in DCE; Right: Time-dependent absorbance at 600 nm (Black trace), and the best 1st order rate fit obtained (red trace).

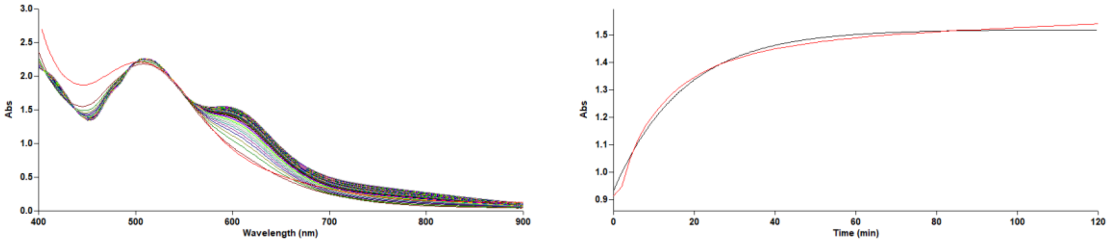

Figure S43. Left: The spectral changes observed in the UV-Vis spectra of **9** for the reaction with Ethylbenzene (263 molar equivalents) at room temperature in DCE; Right: Time-dependent absorbance at 600 nm (Black trace), and the best 1st order rate fit obtained (red trace).

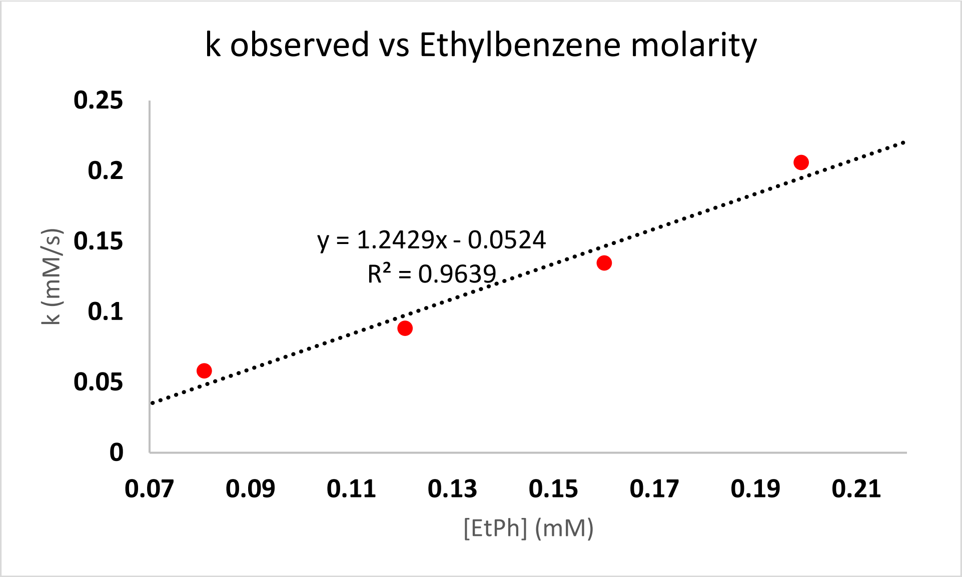

k = k_{obs}/[Mn(IV)-oxo] = 0.027±0.001 s^{-1}

Figure S44. Plot of k_{obs} versus the concentration of ethyl benzene. Dotted line represents the linear fit.

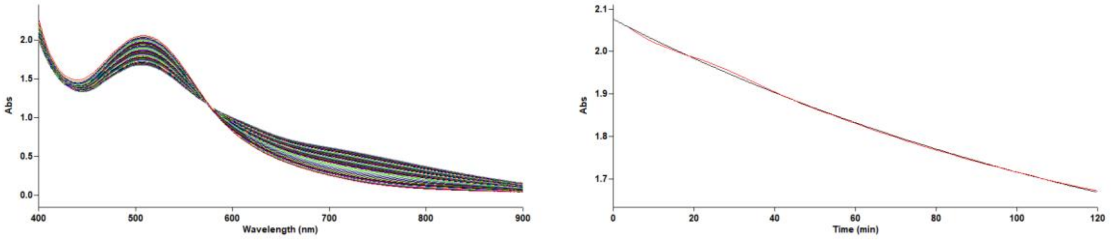

Figure S45. Left: The spectral changes observed in the UV-Vis spectra of **9** for the reaction with Diphenylmethane (77 molar equivalents) at room temperature in DCE; Right: Time-dependent absorbance at 507 nm (Black trace), and the best 1st order rate fit obtained (red trace).

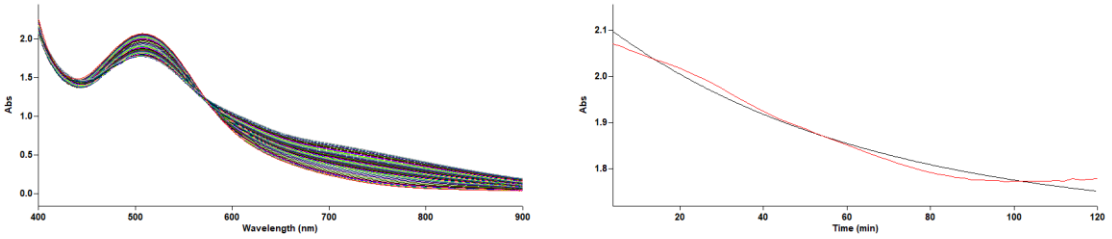

Figure S46. Left: The spectral changes observed in the UV-Vis spectra of **9** for the reaction with Diphenylmethane (116 molar equivalents) at room temperature in DCE; Right: Time-dependent absorbance at 507 nm (Black trace), and the best 1st order rate fit obtained (red trace).

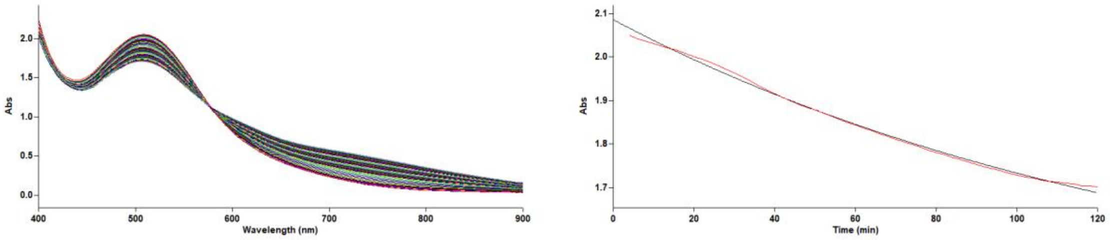

Figure S47. Left: The spectral changes observed in the UV-Vis spectra of **9** for the reaction with Diphenylmethane (154 molar equivalents) at room temperature in DCE; Right: Time-dependent absorbance at 507 nm (Black trace), and the best 1st order rate fit obtained (red trace).

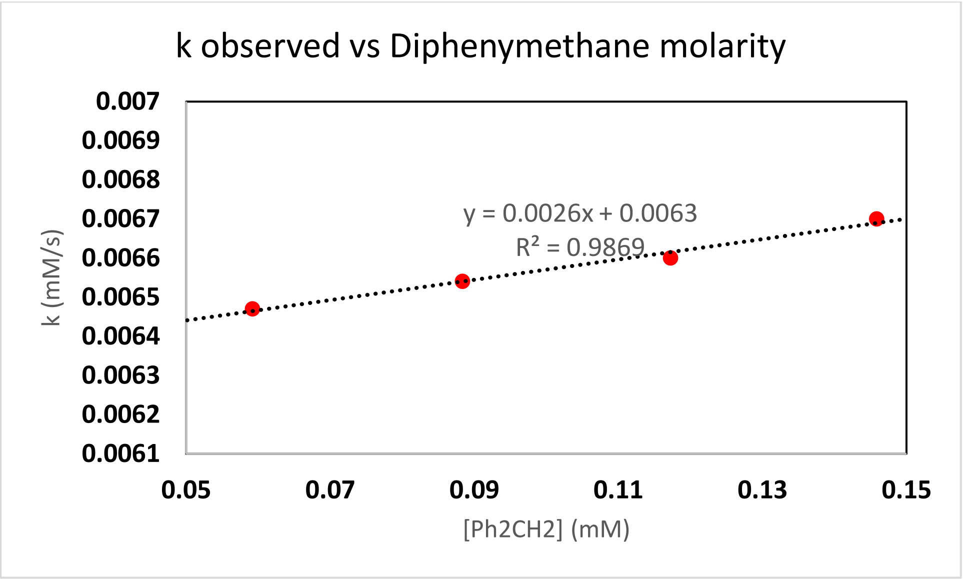

$$\mathbf{k = k_{obs}/[Mn(IV)\text{-}oxo] = 5.7 \cdot 10^{-6} \pm 7 \cdot 10^{-8}\ s^{-1}}$$

Figure S48. Plot of k_{obs} versus the concentration of Diphenylmethane. Black dotted line represents the linear fit.

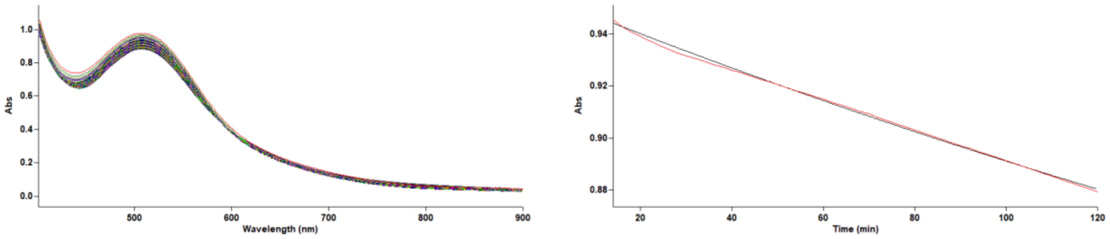

Figure S49. Left: The spectral changes observed in the UV-Vis spectra of **9** for the reaction with Toluene (709 molar equivalents) at room temperature in DCE; Right: Time-dependent absorbance at 507 nm (Black trace), and the best 1st order rate fit obtained (red trace).

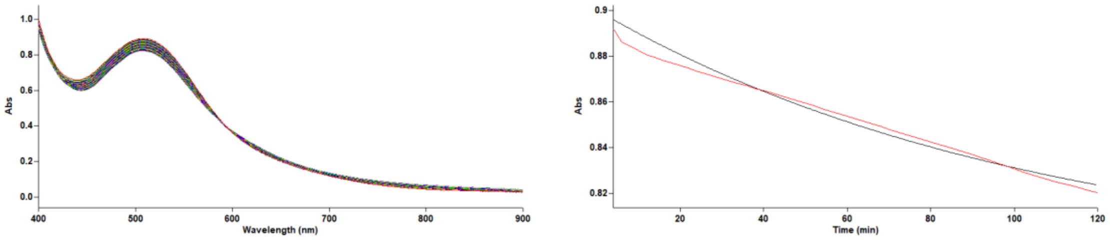

Figure S50. Left: The spectral changes observed in the UV-Vis spectra of **9** for the reaction with Toluene (1,417 molar equivalents) at room temperature in DCE; Right: Time-dependent absorbance at 507 nm (Black trace), and the best 1st order rate fit obtained (red trace).

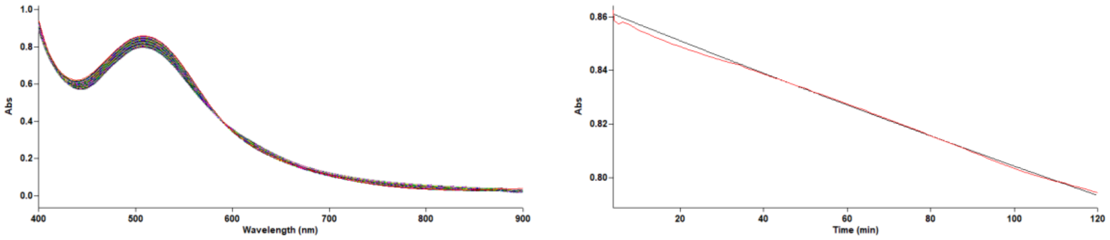

Figure S51. Left: The spectral changes observed in the UV-Vis spectra of **9** for the reaction with Toluene (2,126 molar equivalents) at room temperature in DCE; Right: Time-dependent absorbance at 507 nm (Black trace), and the best 1st order rate fit obtained (red trace).

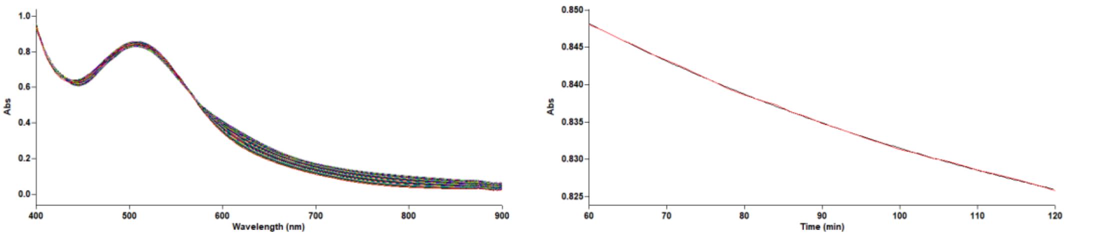

Figure S52. Left: The spectral changes observed in the UV-Vis spectra of **9** for the reaction with Toluene (3,543 molar equivalents) at room temperature in DCE; Right: Time-dependent absorbance at 507 nm (Black trace), and the best 1st order rate fit obtained (red trace).

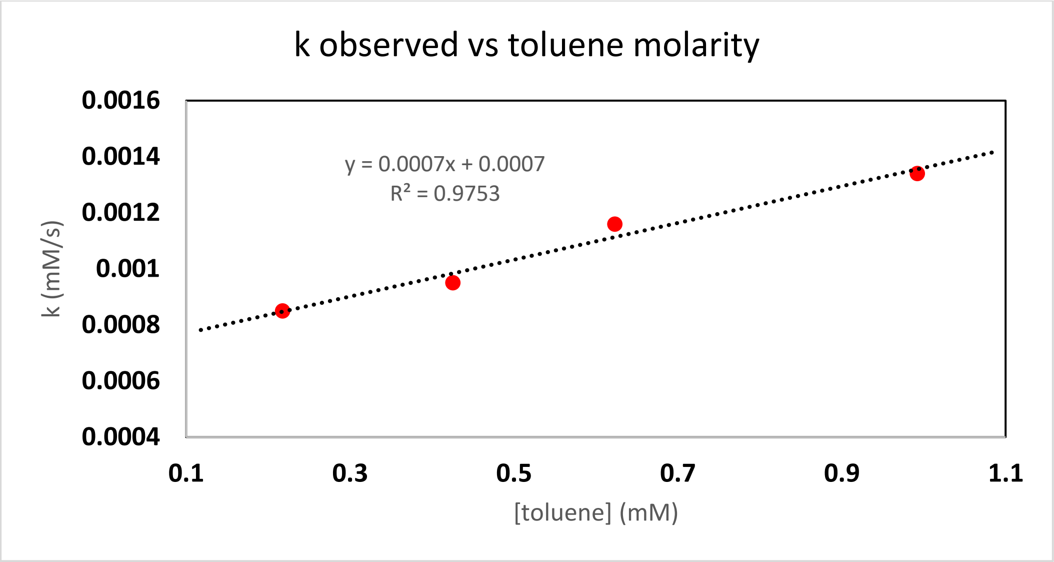

k = k_{obs}/[Mn(IV)-oxo] = $3.7\cdot10^{-6}\pm1\cdot10^{-7}$ s^{-1}

Figure S53. Plot of k_{obs} versus the concentration of toluene. Black dotted line represents the linear fit.

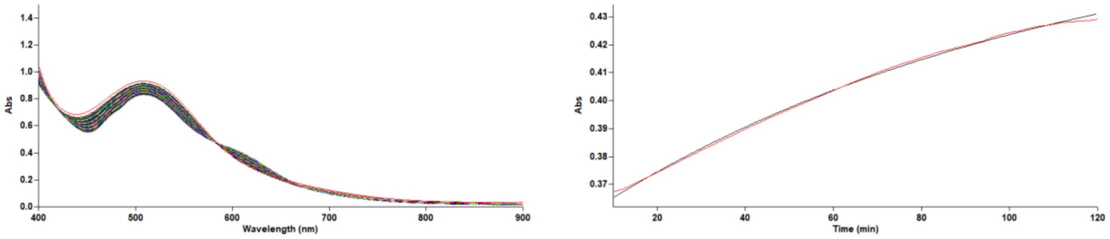

Figure S54. Left: The spectral changes observed in the UV-Vis spectra of **9** for the reaction with Cyclohexene (297 molar equivalents) at room temperature in DCE; Right: Time-dependent absorbance at 605 nm (Black trace), and the best 1st order rate fit obtained (red trace).

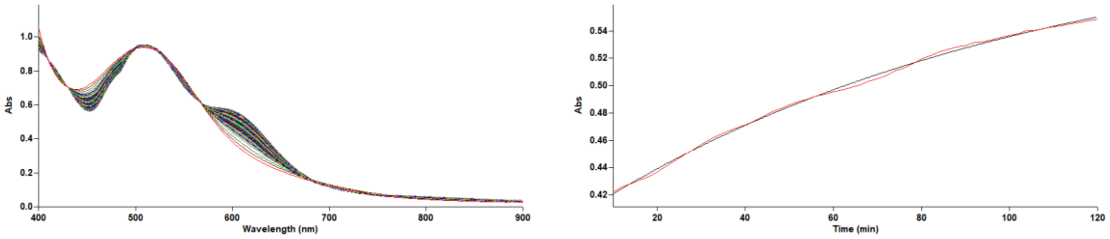

Figure S55. Left: The spectral changes observed in the UV-Vis spectra of **9** for the reaction with Cyclohexene (446 molar equivalents) at room temperature in DCE; Right: Time-dependent absorbance at 605 nm (Black trace), and the best 1st order rate fit obtained (red trace).

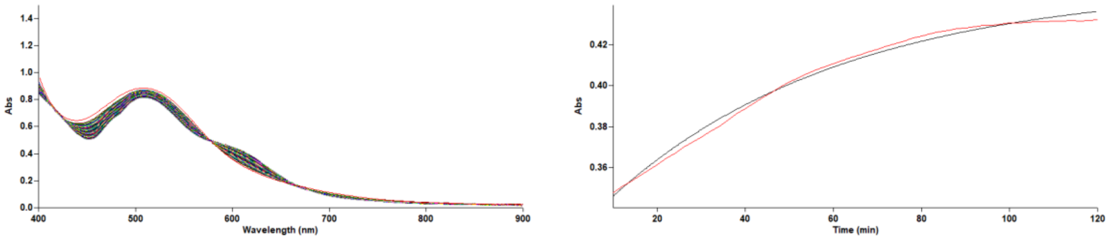

Figure S56. Left: The spectral changes observed in the UV-Vis spectra of **9** for the reaction with Cyclohexene (595 molar equivalents) at room temperature in DCE; Right: Time-dependent absorbance at 605 nm (Black trace), and the best 1st order rate fit obtained (red trace).

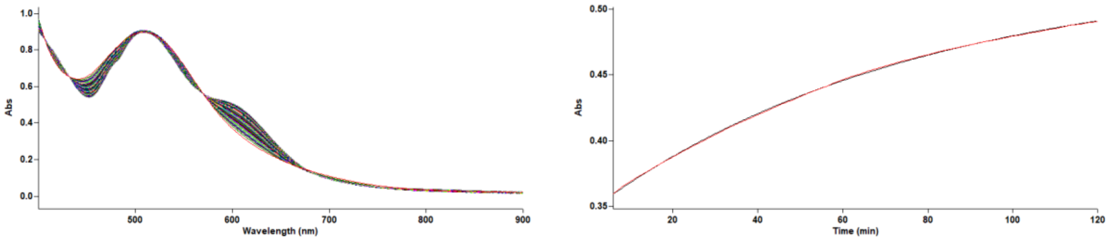

Figure S57. Left: The spectral changes observed in the UV-Vis spectra of **9** for the reaction with Cyclohexene (735 molar equivalents) at room temperature in DCE; Right: Time-dependent absorbance at 605 nm (Black trace), and the best 1st order rate fit obtained (red trace).

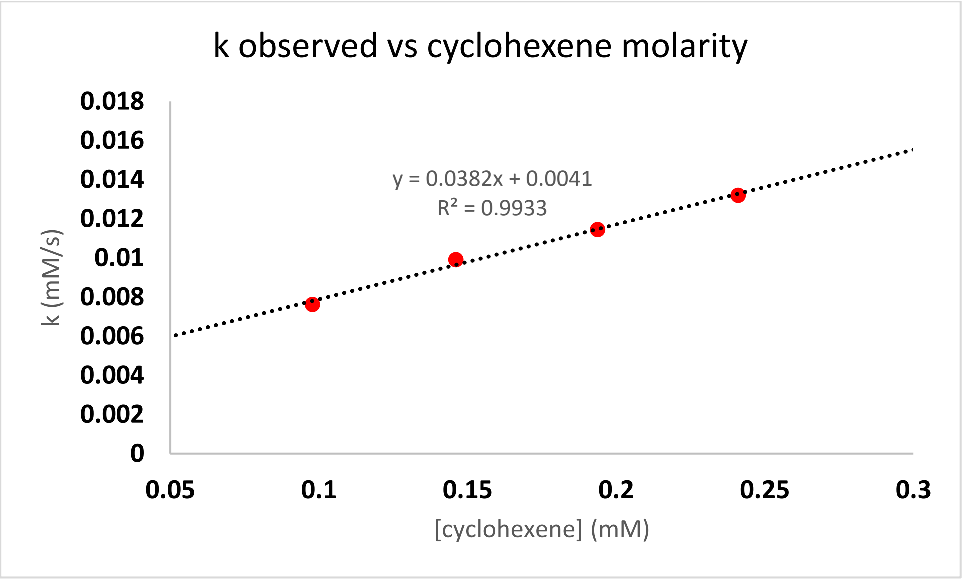

$$\mathbf{k = k_{obs}/[Mn(IV)\text{-}oxo] = 0.0019 \pm 0.0001\ s^{-1}}$$

Figure S58. Plot of k_{obs} versus the concentration of cyclohexene. Black dotted line represents the linear fit.

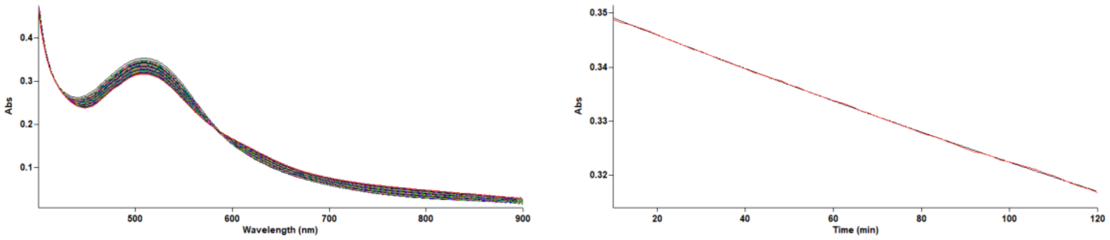

Figure S59. Left: The spectral changes observed in the UV-Vis spectra of **9** for the reaction with *p*-Cymene (735 molar equivalents) at room temperature in DCE; Right: Time-dependent absorbance at 605 nm (Black trace), and the best 1st order rate fit obtained (red trace).

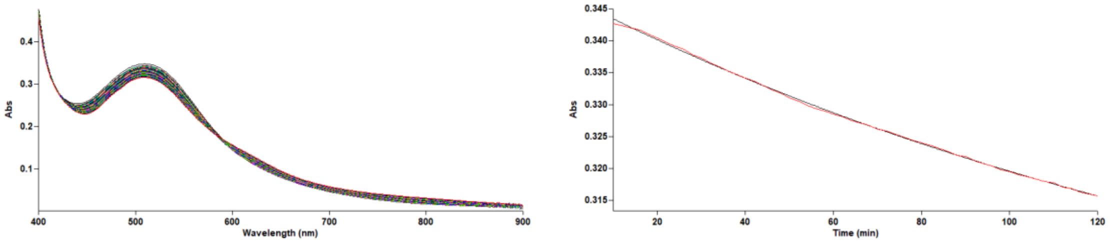

Figure S60. Left: The spectral changes observed in the UV-Vis spectra of **9** for the reaction with *p*-Cymene (1,345 molar equivalents) at room temperature in DCE; Right: Time-dependent absorbance at 605 nm (Black trace), and the best 1st order rate fit obtained (red trace).

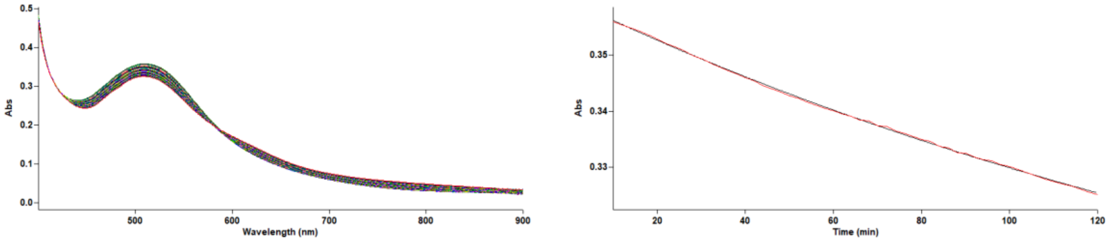

Figure S61. Left: The spectral changes observed in the UV-Vis spectra of **9** for the reaction with *p*-Cymene (2,150 molar equivalents) at room temperature in DCE; Right: Time-dependent absorbance at 605 nm (Black trace), and the best 1st order rate fit obtained (red trace).

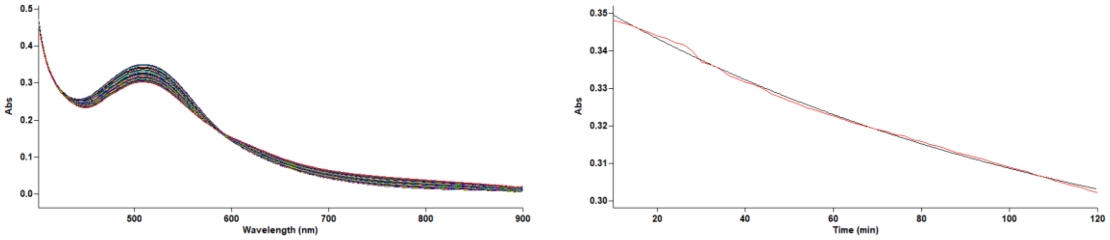

Figure S62. Left: The spectral changes observed in the UV-Vis spectra of **9** for the reaction with *p*-Cymene (2,800 molar equivalents) at room temperature in DCE; Right: Time-dependent absorbance at 605 nm (Black trace), and the best 1st order rate fit obtained (red trace).

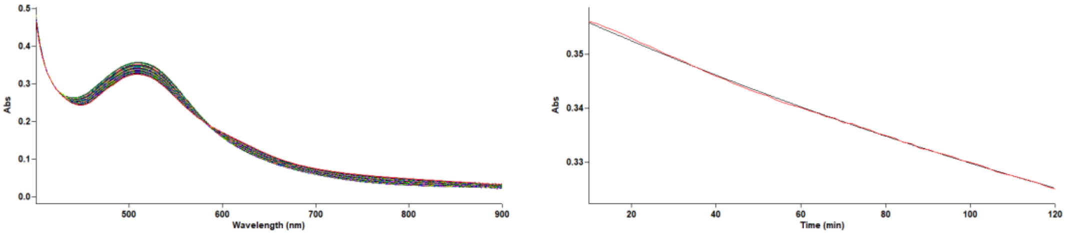

Figure S63. Left: The spectral changes observed in the UV-Vis spectra of **9** for the reaction with *p*-Cymene (5,600 molar equivalents) at room temperature in DCE; Right: Time-dependent absorbance at 605 nm (Black trace), and the best 1st order rate fit obtained (red trace).

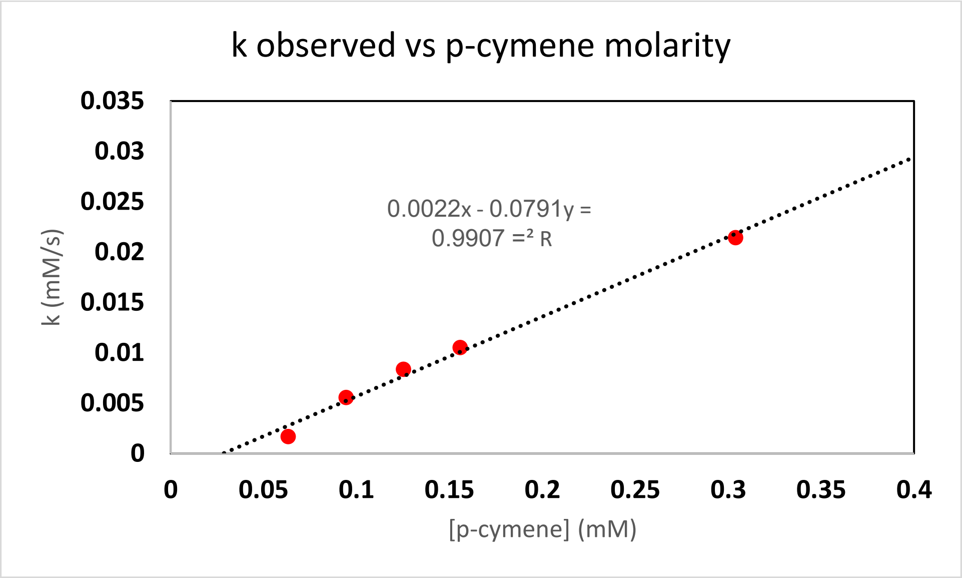

$$\mathbf{k = k_{obs}/[Mn(IV)\text{-}oxo] = 0.0118 \pm 0.0001\ s^{-1}}$$

Figure S64. Plot of k_{obs} versus the concentration of p-cymene. Black dotted line represents the linear fit.

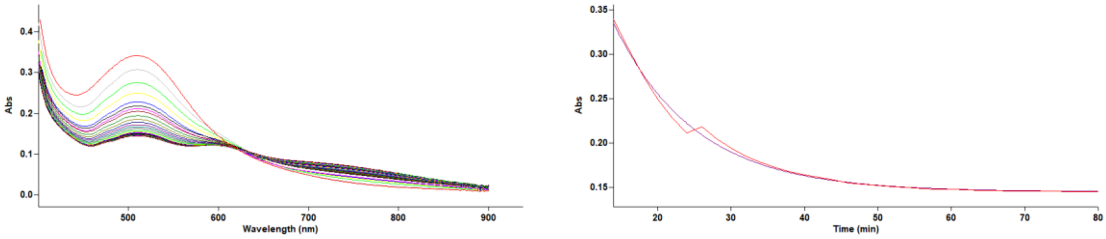

Figure S65. Left: The spectral changes observed in the UV-Vis spectra of **9** for the reaction with 1,4-cyclohexadiene (230 molar equivalents) at room temperature in DCE; Right: Time-dependent absorbance at 605 nm (Black trace), and the best 1st order rate fit obtained (red trace).

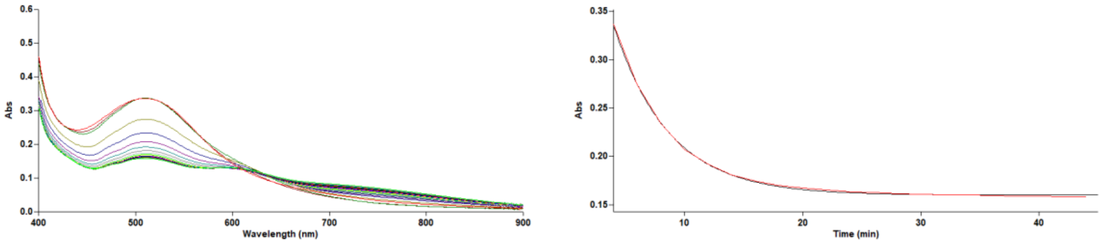

Figure S66. Left: The spectral changes observed in the UV-Vis spectra of **9** for the reaction with 1,4-cyclohexadiene (460 molar equivalents) at room temperature in DCE; Right: Time-dependent absorbance at 605 nm (Black trace), and the best 1st order rate fit obtained (red trace).

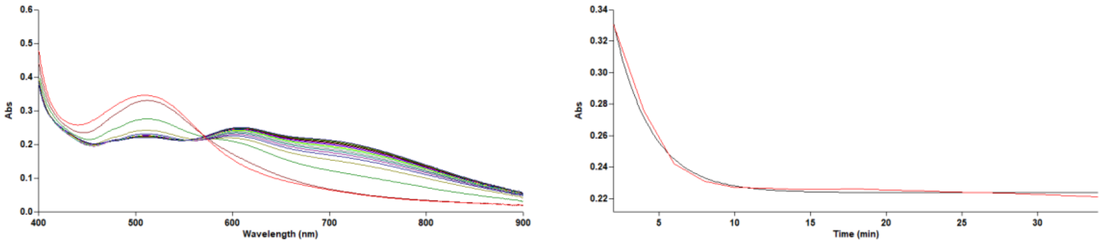

Figure S67. Left: The spectral changes observed in the UV-Vis spectra of **9** for the reaction with 1,4-cyclohexadiene (690 molar equivalents) at room temperature in DCE; Right: Time-dependent absorbance at 605 nm (Black trace), and the best 1st order rate fit obtained (red trace).

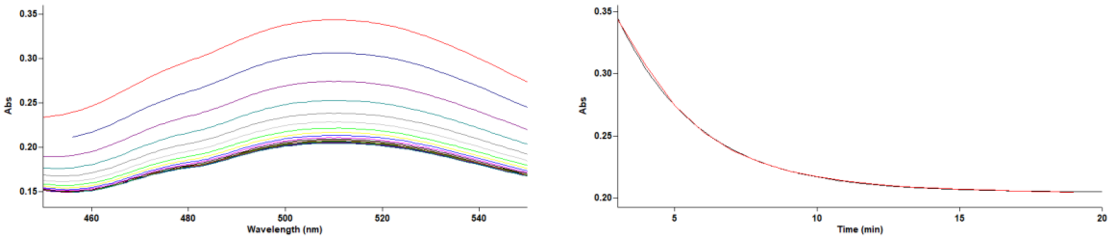

Figure S68. Left: The spectral changes observed in the UV-Vis spectra of **9** for the reaction with 1,4-cyclohexadiene (920 molar equivalents) at room temperature in DCE; Right: Time-dependent absorbance at 605 nm (Black trace), and the best 1st order rate fit obtained (red trace).

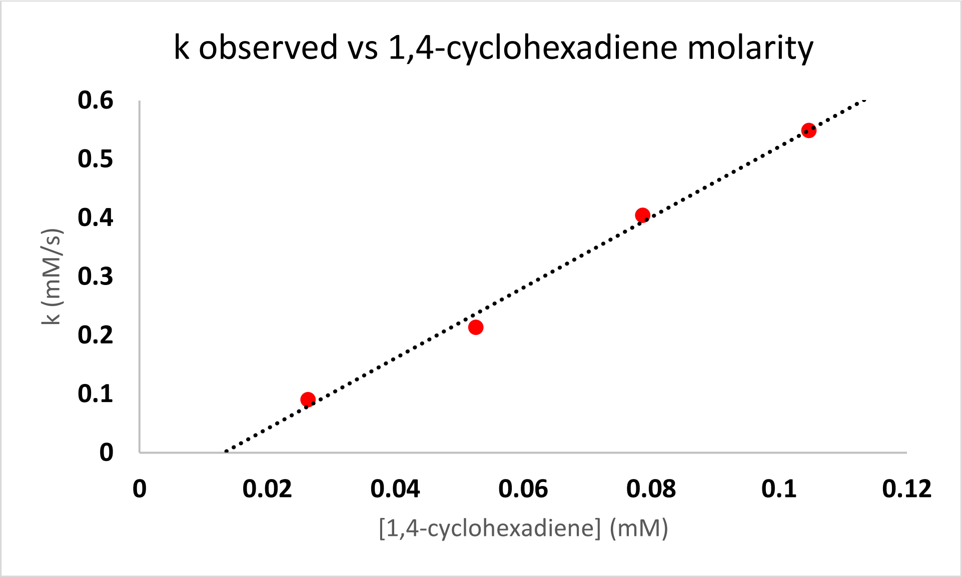

k = k_{obs}/[Mn(IV)-oxo] = 0.870±0.005 s^{-1}

Figure S69. Plot of k_{obs} versus the concentration of 1,4cyclohexadiene. Black dotted line represents the linear fit.

8. Crystallographic information

Table S3. Crystallographic information for compounds **1, 2** and **3** (atropisomer A**)**

Identification code	1	2	3_A
**Empirical formula**	$C_{40}H_{46}O_{12}N_2$	$C_{43}H_{38}O_{16}F_6S_2Cl$	$C_{50}H_{54}N_2O_{10}$
**Formula weight**	746.79	1024.30	842.95
**Temperature/K**	293(1)	295(1)	200(2)
**Crystal system**	triclinic	monoclinic	monoclinic
**Space group**	P-1	C2/c	C2/c
**a/Å**	11.147(3)	21.103(7)	30.145(8)
**b/Å**	12.962(3)	11.302(4)	10.813(3)
**c/Å**	14.414(4)	20.604(6)	27.426(7)
**α/°**	101.231(4)	90.00	90
**β/°**	100.358(4)	106.229(5)	107.324(6)
**γ/°**	106.761(4)	90.00	90
**Volume/Å^3**	1893.2(9)	4718(3)	8534(4)
**Z**	2	4	8
**ρ_{calc}g/cm^3**	1.310	1.442	1.312
**μ/mm^{-1}**	0.097	0.262	0.091
**F(000)**	792.0	2108.0	3584.0
**Crystal size/mm^3**	0.3 × 0.24 × 0.22	0.39 × 0.31 × 0.12	0.06 × 0.06 × 0.03
**Radiation**	MoKα (λ = 0.71073)	MoKα (λ = 0.71073)	MoKα (λ = 0.71073)
**2Θ range for data collection/°**	3.4 to 50	4.12 to 54	2.83 to 50.122
**Index ranges**	-13 ≤ h ≤ 13, -15 ≤ k ≤ 15, -17 ≤ l ≤ 17	-26 ≤ h ≤ 26, -14 ≤ k ≤ 14, -26 ≤ l ≤ 26	-35 ≤ h ≤ 35, -12 ≤ k ≤ 12, -32 ≤ l ≤ 31
**Reflections collected**	17678	22367	37553
**Independent reflections**	6581 [R_{int} = 0.0321, R_{sigma} = 0.0434]	5085 [R_{int} = 0.0995, R_{sigma} = 0.0842]	7529 [R_{int} = 0.1132, R_{sigma} = 0.2205]
**Data/restraints/parameters**	6581/0/501	5085/0/309	7529/0/571
**Goodness-of-fit on F^2**	1.027	1.079	0.836
**Final R indexes [I>=2σ (I)]**	R_1 = 0.0509, wR_2 = 0.1235	R_1 = 0.1010, wR_2 = 0.2726	R_1 = 0.0638, wR_2 = 0.1222
**Final R indexes [all data]**	R_1 = 0.0735, wR_2 = 0.1353	R_1 = 0.1483, wR_2 = 0.2948	R_1 = 0.1995, wR_2 = 0.1598
**Largest diff. peak/hole / e Å^{-3}**	0.17/-0.16	0.58/-1.14	0.52/-0.40

Table S4. Crystallographic information for compounds **3** (atropisomer B)**, 5**, and **7**.

**Identification code**	**3_B**	**5**	**7**
**Empirical formula**	$C_{50}H_{54}N_2O_{10}$	$C_{61}Cl_9H_{67}N_2O_{12}$	$C_{83}H_{56}Cl_3N_{10}O_{11}$
**Formula weight**	842.95	1192.76	1475.72
**Temperature/K**	293(2)	149.98(10)	149.99(10)
**Crystal system**	monoclinic	triclinic	monoclinic
**Space group**	$P2_1/n$	P-1	C2/c
**a/Å**	10.417(2)	12.4584(3)	15.1706(4)
**b/Å**	17.299(3)	14.2608(3)	22.9613(4)
**c/Å**	12.909(2)	18.9926(3)	21.6496(5)
**α/°**	90.00	91.447(2)	90
**β/°**	113.790(3)	101.863(2)	109.822(3)
**γ/°**	90.00	111.615(2)	90
**Volume/Å^3**	2128.6(7)	3051.10(12)	7094.5(3)
**Z**	2	2	4
**ρ_{calc}g/cm^3**	1.315	1.298	1.382
**μ/mm^{-1}**	0.091	0.340	0.202
**F(000)**	896.0	1238.0	3052.0
**Crystal size/mm^3**	0.42 × 0.21 × 0.18	0.197 × 0.06 × 0.051	0.48 × 0.12 × 0.09
**Radiation**	MoKα (λ = 0.71073)	Mo Kα (λ = 0.71073)	Mo Kα (λ = 0.71073)
**2Θ range for data collection/°**	4.18 to 54	4.096 to 50	4.072 to 49.996
**Index ranges**	$-13 \le h \le 13$, $-21 \le k \le 22$, $-16 \le l \le 16$	$-14 \le h \le 14$, $-16 \le k \le 16$, $-21 \le l \le 22$	$-18 \le h \le 18$, $-27 \le k \le 27$, $-25 \le l \le 25$
**Reflections collected**	22877	38256	56811
**Independent reflections**	4640 [R_{int} = 0.0328, R_{sigma} = 0.0265]	10745 [R_{int} = 0.0317, R_{sigma} = 0.0308]	6247 [R_{int} = 0.0394, R_{sigma} = 0.0191]
**Data/restraints/parameters**	4640/0/286	10745/17/764	6247/49/562
**Goodness-of-fit on F^2**	1.225	1.027	1.026
**Final R indexes [I>=2σ (I)]**	R_1 = 0.0775, wR_2 = 0.1598	R_1 = 0.0543, wR_2 = 0.1424	R_1 = 0.0606, wR_2 = 0.1692
**Final R indexes [all data]**	R_1 = 0.0850, wR_2 = 0.1640	R_1 = 0.0650, wR_2 = 0.1491	R_1 = 0.0707, wR_2 = 0.1790
**Largest diff. peak/hole / e Å^{-3}**	0.27/-0.25	1.18/-0.89	0.69/-0.61

Table S5. Crystallographic information for compounds **8**·H_2O**,** **9**, and **10**.

**Complex**	**8**·H_2O	**9**	**10**
**Empirical formula**	$C_{84}Cl_4H_{60}MnN_{10}O_{12}$	$C_{83}H_{54}Cl_2MnN_{10}O_{12}$	$C_{83.07}H_{56.35}Cl_2MnN_{10}O_{12}$
**Formula weight**	1598.16	1509.20	1512.41
**Temperature/K**	150.00(10)	175.15	169(20)
**Crystal system**	monoclinic	monoclinic	monoclinic
**Space group**	C2/c	$P2_1/n$	C2/c
**a/Å**	15.1700(5)	11.4898(5)	15.1502(7)
**b/Å**	22.8821(5)	26.6532(9)	22.7752(7)
**c/Å**	21.6828(7)	23.9649(8)	21.7691(10)
**α/°**	90	90	90
**β/°**	110.141(4)	90.972(4)	109.611(5)
**γ/°**	90	90	90
**Volume/Å^3**	7066.3(4)	7338.0(5)	7075.7(6)
**Z**	4	4	4
**ρ_{calc}g/cm^3**	1.502	1.366	1.420
**Radiation**	Mo Kα (λ = 0.71073)	Mo Kα (λ = 0.71073)	Mo Kα (λ = 0.71073)
**Reflections collected**	23264	37091	15403
**Independent reflections**	6185 [R_{int} = 0.0260, R_{sigma} = 0.0230]	12433 [R_{int} = 0.1314, R_{sigma} = 0.1218]	6177 [R_{int} = 0.0370, R_{sigma} = 0.0411]
**Goodness-of-fit on F^2**	1.062	1.071	1.053
**Final R indexes [I>=2σ (I)]**	R_1 = 0.0740, wR_2 = 0.2298	R_1 = 0.1652, wR_2 = 0.3612	R_1 = 0.0803, wR_2 = 0.2197
**Final R indexes [all data]**	R_1 = 0.0835, wR_2 = 0.2407	R_1 = 0.2332, wR_2 = 0.4026	R_1 = 0.0974, wR_2 = 0.2320
**Largest diff. peak/hole / e Å^{-3}**	0.79/-0.81	1.75/-1.30	1.74/-0.84

9. Ab-initio calculations and orbital analysis

Table S6. Comparison between experimental EPR parameters (from simulation) of **8** and computed (CASSCF/NEVPT2) spin Hamiltonian parameters for **8***.

	**8**	**8***	
	**Exp**	**CAS (5,5)**	**NEV (5,5)**
***D*/cm^{-1} (*D* Strain)**	**0.267 (0.035)**	0.15	0.19
E/D	**0.007 (0.0006)**	0.019	0.039
**g_1**	**2.05**	2.002	2.002
**g_2**	**2.004**	2.002	2.002
**g_3**	**1.97**	2.002	2.002
**A_1, A_2, A_3 /MHz**	**220 200 300**	-	-
**lwpp**	**[12 9]**	-	-

Table S7. Comparison between experimental EPR parameters (from simulation) of **9** and computed (CASSCF/NEVPT2) spin Hamiltonian parameters for **9*** with smaller and larger active spaces.

	**9**	**9***							
	**Experiment**	**Mult = 4, Root = 10**				**Mult = 4, Root = 10 Mult=2, root=9**			
		**CAS (3,5)**	**NEV (3,5)**	**CAS (11,9)**	**NEV (11,9)**	**CAS (3,5)**	**NEV (3,5)**	**CAS (11,9)**	**NEV (11,9)**
***D*/cm^{-1} (*D* strain)**	**-0.914 (0.15 0.0067)**	1.43	-0.76	-12.02	-1.60	0.36	-1.04	-5.13	-1.41
E/D	**0.33**	0.23	0.084	0.27	0.11	0.28	0.07	0.26	0.22
**g_1**	**2.00**	1.95	1.97	1.60	1.95	1.95	1.94	1.60	1.97
**g_2**	**1.98**	1.96	1.98	1.84	1.99	1.96	1.96	1.90	1.99
**g_3**	**1.70**	1.98	1.98	1.87	1.99	1.97	1.97	1.90	1.99
**A_1, A_2, A_3 /MHz**	**200, 300, 245**	-	-	-	-	-	-	-	-
**lwpp**	**[5 5]**	-	-	-	-	-	-	-	-

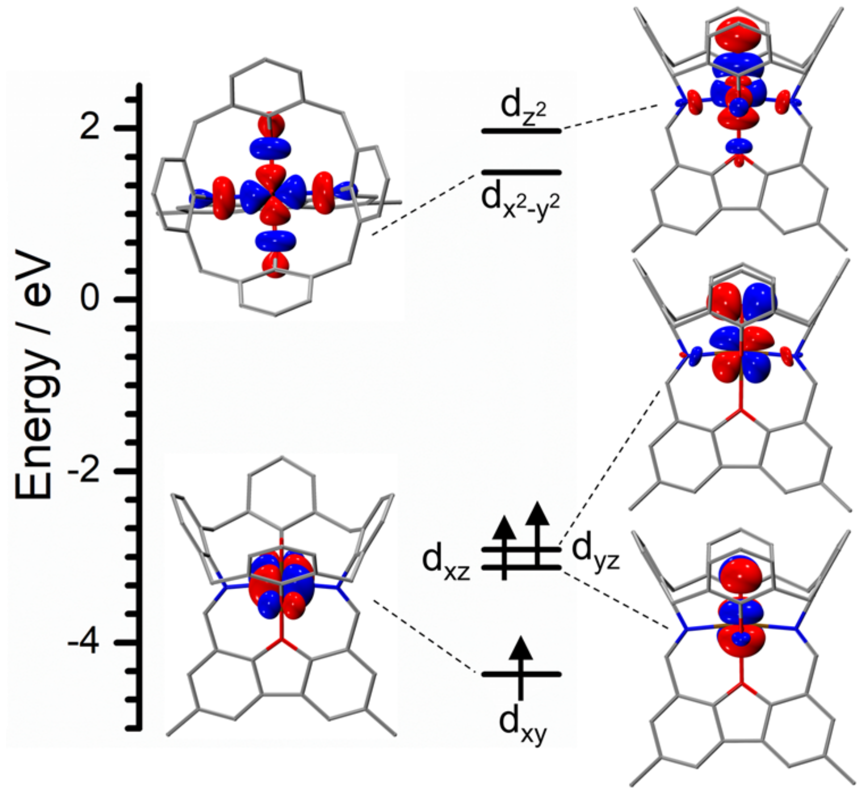

Figure S70. CASSCF/NEVPT2 active orbitals of **9*** computed with a CAS(3,5) active space.

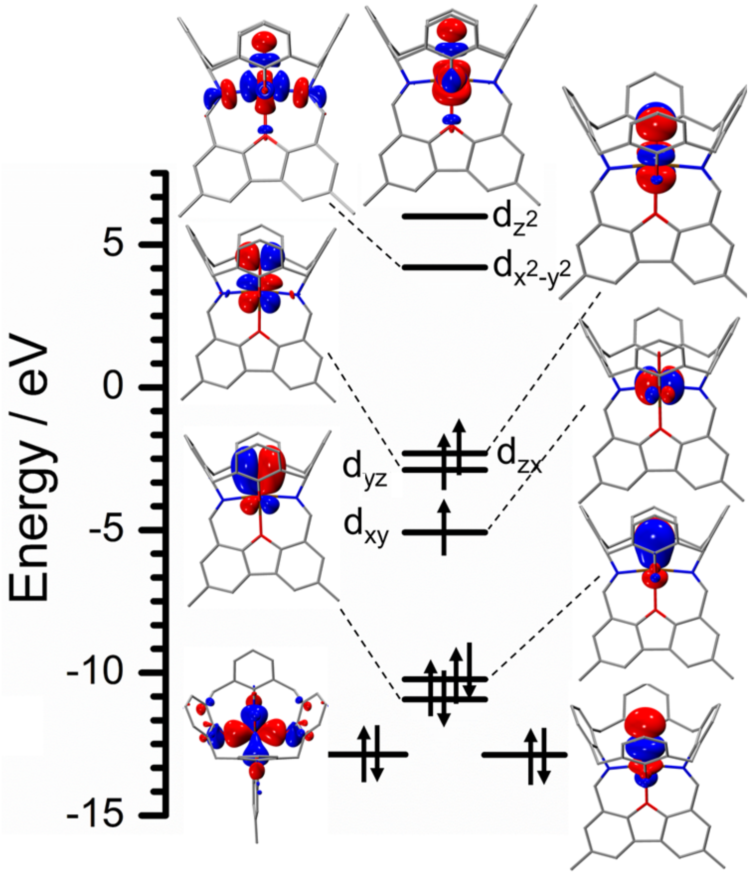

Figure S71. CASSCF/NEVPT2 active orbitals of **9*** computed with a CAS(11,9) active space.

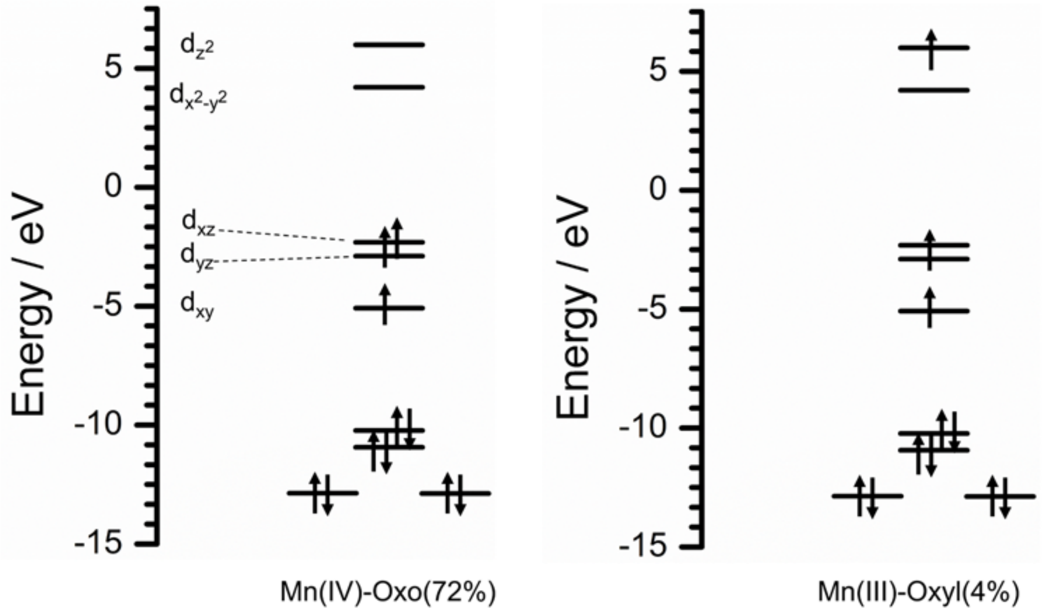

Figure S72. CASSCF computed percent contributions from the major electronic configurations to the ground state of **9***

10. HRMS spectra of compounds 1-11

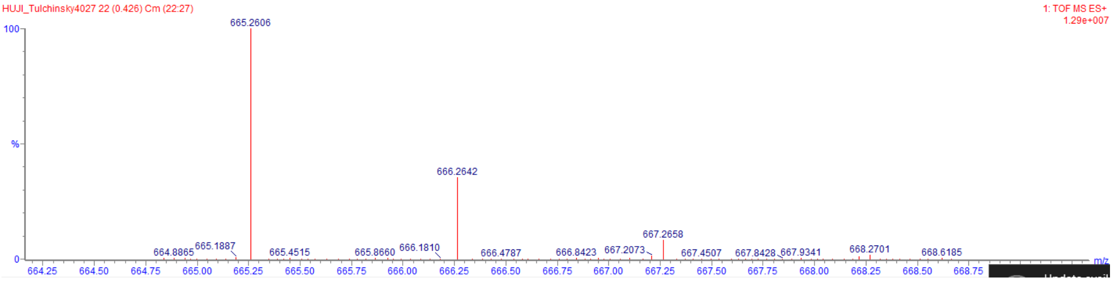

Figure S73. Mass spectrum of **1**

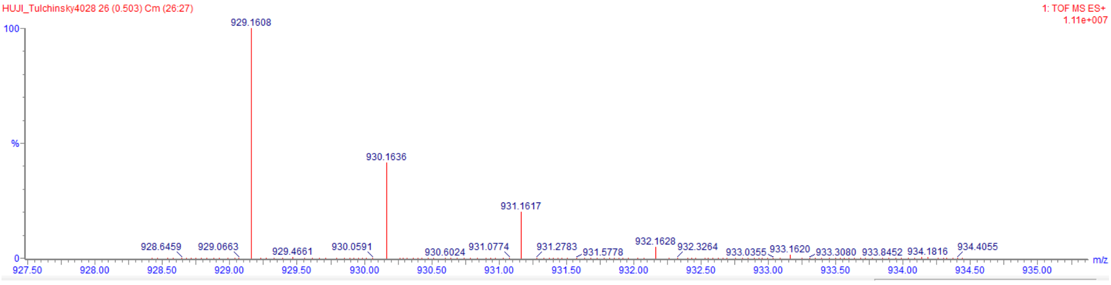

Figure S74. Mass spectrum of **2**

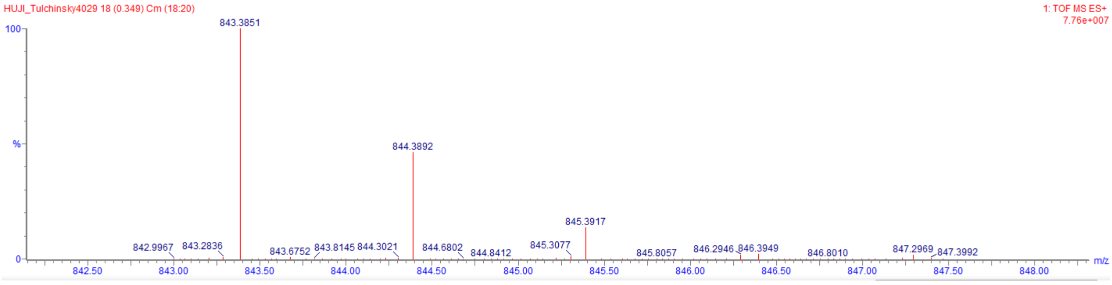

Figure S75. Mass spectrum of **3**

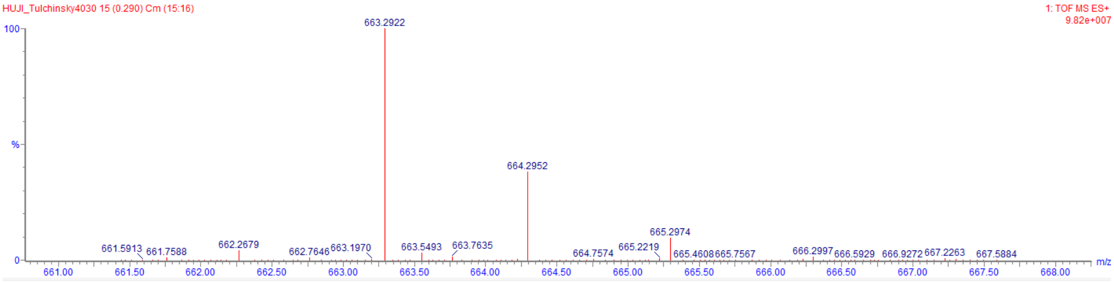

Figure S76. Mass spectrum of **4**

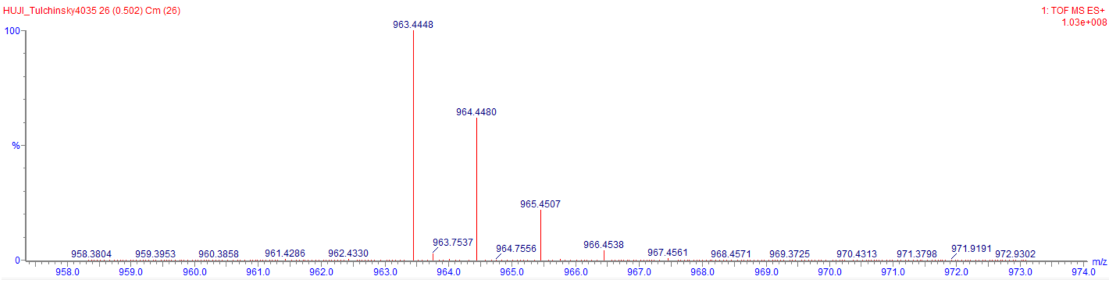

Figure S77. Mass spectrum of **5**

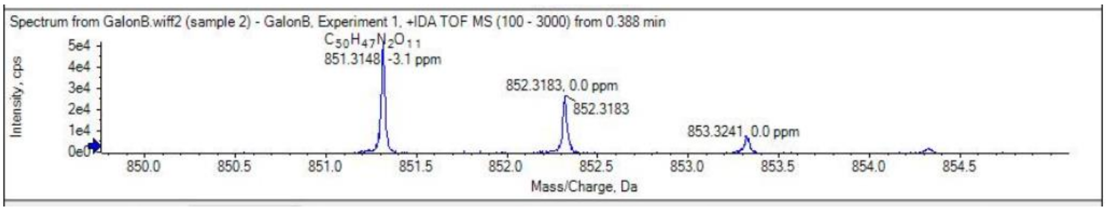

Figure S78. Mass spectrum of **6**

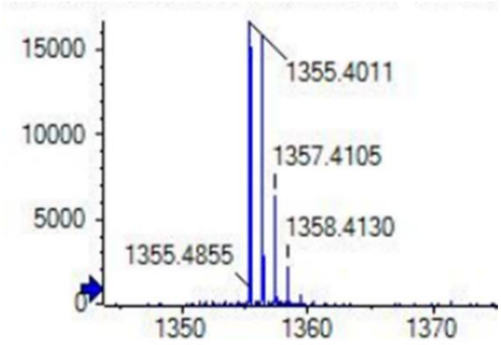

Figure S79. Mass spectrum of **7**

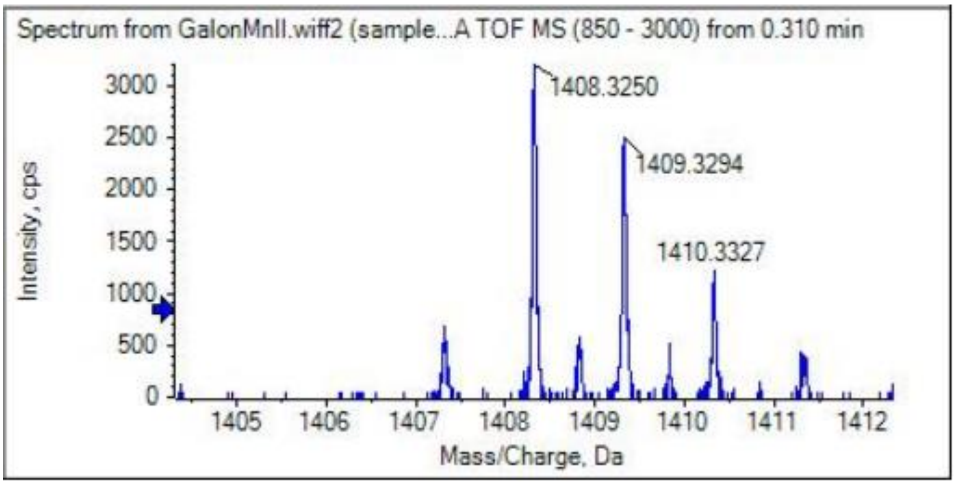

Figure S80. Mass spectrum of **8**

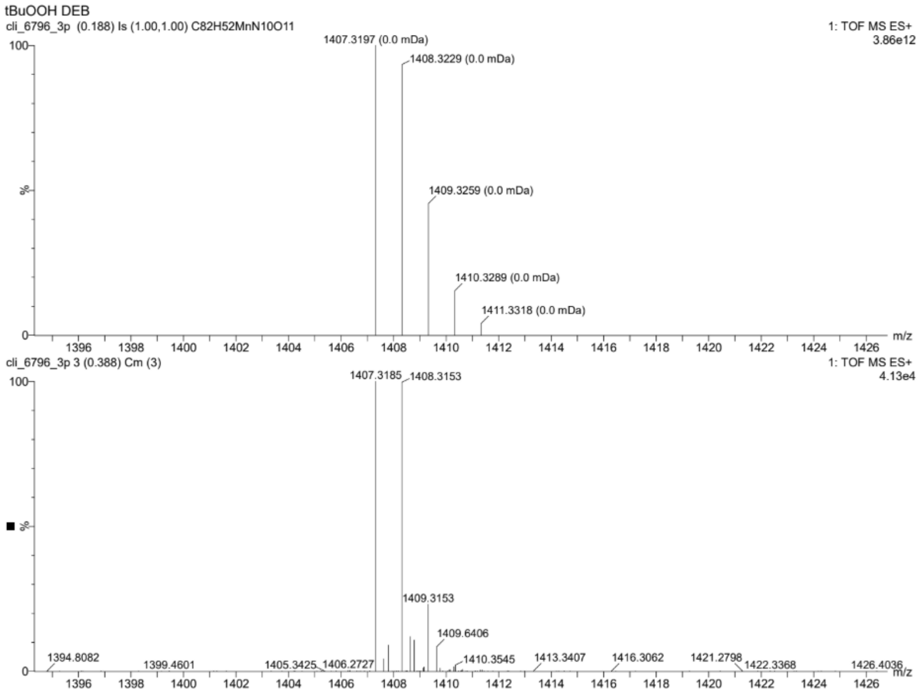

Figure S81. Mass spectrum of complex **10** generated from the reaction of **8** and ^{t}BuOOH in 1,2-DFB at r.t. (top: simulated and bottom: experimental).

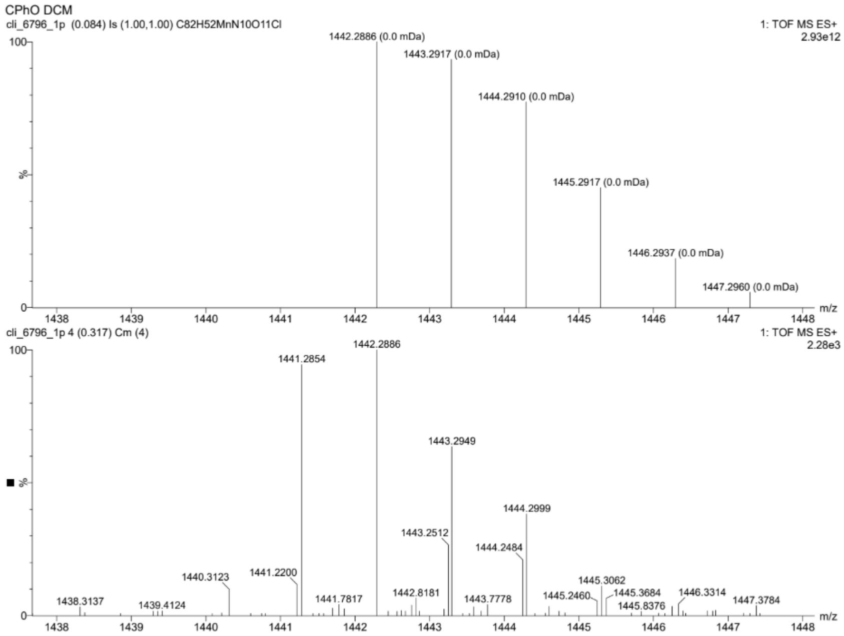

Figure S82. Mass spectrum of complex **11** generated from the reaction of **8** and ^{t}BuOOH in DCM at r.t. (top: simulated and bottom: experimental spectrum).

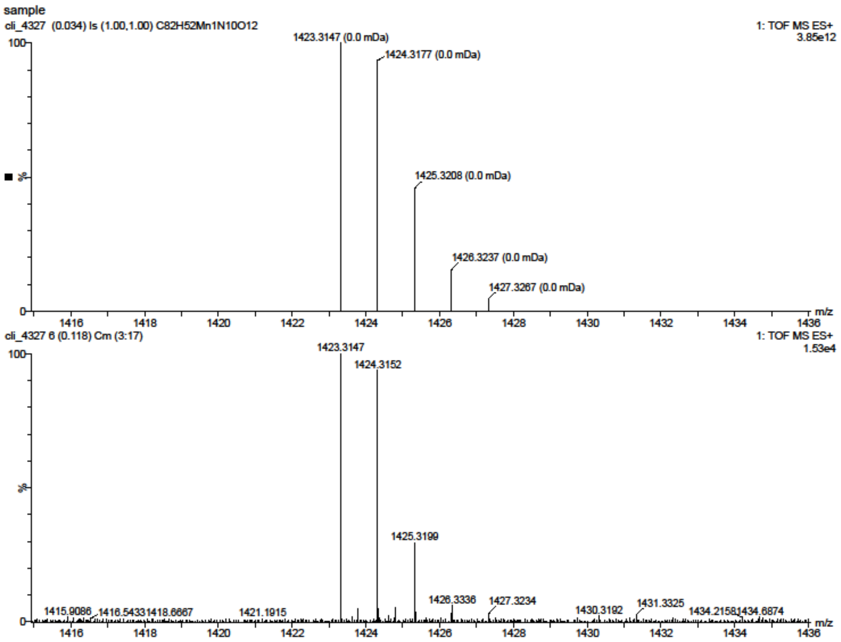

Figure S83. Mass spectrum of intermediate **9** generated from the reaction of **8** and sPhIO in 1,2-DCE at r.t (top: simulated and bottom: experimental).

11. References.

1. Sheldrick, G. M., Crystal structure refinement with SHELXL. *Acta Crystallographica Section C: Structural Chemistry* **2015,** *71* (1), 3-8.

2. Dolomanov, O. V.; Bourhis, L. J.; Gildea, R. J.; Howard, J. A. K.; Puschmann, H., OLEX2: a complete structure solution, refinement and analysis program. *J. Appl. Crystallogr.* **2009,** *42* (2), 339-341.

3. Neese, F., Software update: The ORCA program system—Version 5.0. *WIREs Computational Molecular Science* **2022,** *12* (5), e1606.

4. Grimme, S.; Ehrlich, S.; Goerigk, L., Effect of the damping function in dispersion corrected density functional theory. *J. Comput. Chem.* **2011,** *32* (7), 1456-1465.

5. Frisch, M. J.; Trucks, G. W.; Schlegel, H. B.; Scuseria, G. E.; Robb, M. A.; Cheeseman, J. R.; Scalmani, G.; Barone, V.; Petersson, G. A.; Nakatsuji, H.; Li, X.; Caricato, M.; Marenich, A. V.; Bloino, J.; Janesko, B. G.; Gomperts, R.; Mennucci, B.; Hratchian, H. P.; Ortiz, J. V.; Izmaylov, A. F.; Sonnenberg, J. L.; Williams; Ding, F.; Lipparini, F.; Egidi, F.; Goings, J.; Peng, B.; Petrone, A.; Henderson, T.; Ranasinghe, D.; Zakrzewski, V. G.; Gao, J.; Rega, N.; Zheng, G.; Liang, W.; Hada, M.; Ehara, M.; Toyota, K.; Fukuda, R.; Hasegawa, J.; Ishida, M.; Nakajima, T.; Honda, Y.; Kitao, O.; Nakai, H.; Vreven, T.; Throssell, K.; Montgomery Jr., J. A.; Peralta, J. E.; Ogliaro, F.; Bearpark, M. J.; Heyd, J. J.; Brothers, E. N.; Kudin, K. N.; Staroverov, V. N.; Keith, T. A.; Kobayashi, R.; Normand, J.; Raghavachari, K.; Rendell, A. P.; Burant, J. C.; Iyengar, S. S.; Tomasi, J.; Cossi, M.; Millam, J. M.; Klene, M.; Adamo, C.; Cammi, R.; Ochterski, J. W.; Martin, R. L.; Morokuma, K.; Farkas, O.; Foresman, J. B.; Fox, D. J. *Gaussian 16 Rev. C.01*, Wallingford, CT, 2016.

6. Becke, A. D., Density-functional thermochemistry. III. The role of exact exchange. *J. Chem. Phys* **1993,** *98* (7), 5648-5652.

7. Becke, A. D., Density-functional exchange-energy approximation with correct asymptotic behavior. *Phys. Rev. A.* **1988,** *38* (6), 3098-3100.

8. Schäfer, A.; Horn, H.; Ahlrichs, R., Fully optimized contracted Gaussian basis sets for atoms Li to Kr. *J. Chem. Phys* **1992,** *97* (4), 2571-2577.

9. Schäfer, A.; Huber, C.; Ahlrichs, R., Fully optimized contracted Gaussian basis sets of triple zeta valence quality for atoms Li to Kr. *J. Chem. Phys* **1994,** *100* (8), 5829-5835.

10. Ditchfield, R.; Hehre, W. J.; Pople, J. A., Self-Consistent Molecular-Orbital Methods. IX. An Extended Gaussian-Type Basis for Molecular-Orbital Studies of Organic Molecules. *J. Chem. Phys* **1971,** *54* (2), 724-728.

11. Miertuš, S.; Scrocco, E.; Tomasi, J., Electrostatic interaction of a solute with a continuum. A direct utilizaion of AB initio molecular potentials for the prevision of solvent effects. *Chem. Phys.* **1981,** *55* (1), 117-129.

12. Miertuš̃, S.; Tomasi, J., Approximate evaluations of the electrostatic free energy and internal energy changes in solution processes. *Chem. Phys.* **1982,** *65* (2), 239-245.

13. Cossi, M.; Barone, V.; Cammi, R.; Tomasi, J., Ab initio study of solvated molecules: a new implementation of the polarizable continuum model. *Chem. Phys. Lett.* **1996,** *255* (4), 327-335.

14. Perdew, J. P., Density-functional approximation for the correlation energy of the inhomogeneous electron gas. *Phys. Rev. B.* **1986,** *33* (12), 8822-8824.

15. Becke, A. D., Density functional calculations of molecular bond energies. *J. Chem. Phys* **1986,** *84* (8), 4524-4529.

16. Perdew, J. P.; Zunger, A., Self-interaction correction to density-functional approximations for many-electron systems. *Phys. Rev. B.* **1981,** *23* (10), 5048-5079.

17. Reiher, M., Douglas–Kroll–Hess Theory: a relativistic electrons-only theory for chemistry. *Theor. Chem. Acc.* **2006,** *116* (1), 241-252.

18. Angeli, C.; Cimiraglia, R.; Evangelisti, S.; Leininger, T.; Malrieu, J. P., Introduction of n-electron valence states for multireference perturbation theory. *J. Chem. Phys* **2001,** *114* (23), 10252-10264.

19. Maurice, R.; Bastardis, R.; Graaf, C. d.; Suaud, N.; Mallah, T.; Guihéry, N., Universal Theoretical Approach to Extract Anisotropic Spin Hamiltonians. *J. Chem. Theory Comput.* **2009,** *5* (11), 2977-2984.

20. Lu, T.; Chen, F., Multiwfn: A multifunctional wavefunction analyzer. *J. Comput. Chem.* **2012,** *33* (5), 580-592.

21. Lu, T., A comprehensive electron wavefunction analysis toolbox for chemists, Multiwfn. *J. Chem. Phys* **2024,** *161* (8), 082503.

22. Glendening, E. D.; Landis, C. R.; Weinhold, F., NBO 7.0: New vistas in localized and delocalized chemical bonding theory. *J. Comput. Chem.* **2019,** *40* (25), 2234-2241.

23. Stoll, S.; Schweiger, A., EasySpin, a comprehensive software package for spectral simulation and analysis in EPR. *J. Magn. Reson.* **2006,** *178* (1), 42-55.

24. Skene, W. G.; Berl, V.; Risler, H.; Khoury, R.; Lehn, J.-M., Selective product amplification of thymine photodimer by recognition-directed supramolecular assistance. *Org. Biomol. Chem.* **2006,** *4* (19), 3652-3663.
25. Macikenas, D.; Skrzypczak-Jankun, E.; Protasiewicz, J. D., A New Class of Iodonium Ylides Engineered as Soluble Primary Oxo and Nitrene Sources. *J. Am. Chem. Soc.* **1999,** *121* (30), 7164-7165.
26. Pedroarena, J. R.; Nell, B. P.; Zakharov, L. N.; Tyler, D. R., Synthesis of Unsymmetrical Bis(phosphine) Oxides and Their Phosphines via Secondary Phosphine Oxide Precursors. *Journal of Inorganic and Organometallic Polymers and Materials* **2020,** *30* (1), 196-205.
